# Synthesis of 3-(5-amino-1*H*-1,2,4-triazol-3-yl)propanamides and their tautomerism†

**DOI:** 10.1039/c8ra04576c

**Published:** 2018-06-19

**Authors:** Felicia Phei Lin Lim, Lin Yuing Tan, Edward R. T. Tiekink, Anton V. Dolzhenko

**Affiliations:** School of Pharmacy, Monash University Malaysia Jalan Lagoon Selatan, Bandar Sunway Selangor Darul Ehsan 47500 Malaysia dolzhenkoav@gmail.com anton.dolzhenko@monash.edu +60-3-5514-6364 +60-3-5514-5867; Research Centre for Crystalline Materials, School of Science and Technology, Sunway University Bandar Sunway Selangor Darul Ehsan 47500 Malaysia; School of Pharmacy and Biomedical Sciences, Curtin Health Innovation Research Institute, Faculty of Health Sciences, Curtin University GPO Box U1987 Perth Western Australia 6845 Australia

## Abstract

Two complementary pathways for the preparation of *N*-substituted 3-(5-amino-1*H*-1,2,4-triazol-3-yl)propanamides (5) were proposed and successfully realized in the synthesis of 20 representative examples. These methods use the same types of starting materials *viz*. succinic anhydride, aminoguanidine hydrochloride, and a variety of amines. The choice of the pathway and sequence of the introduction of reagents to the reaction depended on the amine nucleophilicity. The first pathway started with the preparation of *N*-guanidinosuccinimide, which then reacted with amines under microwave irradiation to afford 5. The desired products were successfully obtained in the reaction with aliphatic amines (primary and secondary) *via* a nucleophilic opening of the succinimide ring and the subsequent recyclization of the 1,2,4-triazole ring. This approach however failed when less nucleophilic aromatic amines were used. Therefore, an alternative pathway, with the initial preparation of *N*-arylsuccinimides and their subsequent reaction with aminoguanidine hydrochloride under microwave irradiation, was applied. The annular prototropic tautomerism in the prepared 1,2,4-triazoles 5 was studied using NMR spectroscopy and X-ray crystallography.

## Abstract 2

Two complementary pathways for the preparation of N-substituted 3-(5-amino-1*H*-1,2,4-triazol-3-yl)propanamides were proposed and successfully realized in the synthesis of 20 representative examples.

## Graphical abstract

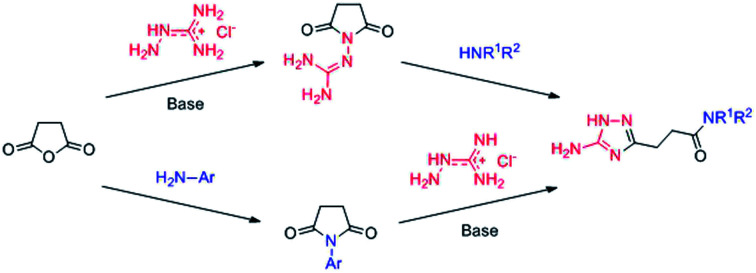

## Introduction

1,2,4-Triazoles attract considerable attention in organic and medicinal chemistry due to their wide spectrum of pharmacological activities.^1^ They have been reported to possess antiviral,^2^ antibacterial,^3^ antifungal,^4^ anticancer,^5^ and anticonvulsant^6^ properties. Among the 1,2,4-triazoles, 3(5)-amino-1,2,4-triazoles were recently reported to be potent inhibitors of kinases,^7^ lysine-specific demethylase 1,^8^ and acidic mammalian chitinase.^9^ Additionally, 3(5)-amino-1,2,4-triazoles are also known as important building blocks for the construction of bioactive triazole-fused heterocycles.^10^

Due to practical significance of 3(5)-amino-1,2,4-triazoles, there is an ongoing demand for new efficient methods of their synthesis. The most common synthetic methods for the preparation of 3(5)-amino-1,2,4-triazoles involve intramolecular cyclocondensation of amidoguanidines,^11^ thermal condensation of *N*-cyanoimidates with hydrazine,^12^ and 1,3-dipolar cycloaddition of hydrazonoyl derivatives and carbodiimides.^13^ However, the existing approaches suffer from several drawbacks, including multi-step preparation process, harsh reaction conditions, poor selectivity of reactions and tedious purification procedures.

Over the past decade, there has been a substantial increase in the application of microwave irradiation in organic synthesis. It is a valuable tool used for improving the outcome of reactions, often resulting in higher yield and product purity.^14^ Utilization of microwave irradiation for the synthesis of 1,2,4-triazoles has shown to provide practical and economical advantages.^11*e*,15^ Herein, we report the development of efficient microwave-assisted methods for the preparation of 3-(5-amino-1*H*-1,2,4-triazol-3-yl)propanamides (5).

Annular prototropic tautomerism is an interesting phenomenon often observed in compounds possessing a 1,2,4-triazole ring in their structure. The tautomeric preferences and factors affecting equilibrium between tautomers have been studied theoretically and experimentally, thermodynamically and kinetically due to their importance in determining chemical and biological properties of compounds.^16^ We applied NMR spectroscopy to explore tautomerism in the prepared compounds and report here our findings. X-ray crystallography was used to determine a position of the triazole ring hydrogen in the solid state.

## Results & discussion

### Synthesis of 3-(5-amino-1*H*-1,2,4-triazol-3-yl)propanamides (5) from *N*-guanidinosuccinimide (2)

**Scheme 1:**
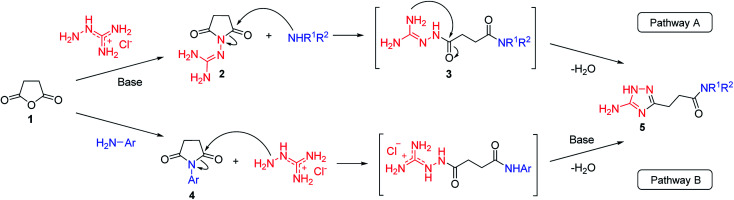
Two pathways to the synthesis of *N*-substituted 3-(5-amino-1*H*-1,2,4-triazol-3-yl)propanamides (5).

Our initial synthesis design involved the preparation of 3-(5-amino-1*H*-1,2,4-triazol-3-yl)propanamides (5) from *N*-guanidinosuccinimide (2) using nucleophilic ring opening of the latter with amines followed by the cyclocondensation of intermediates 3 and closure of the 1,2,4-triazole ring (Scheme 1, Pathway A). We also proposed that performing the process under high temperature using microwave irradiation would be efficient to have a tandem of these reactions happened in the one-pot fashion.

In the model reaction, *N*-guanidinosuccinimide (2), prepared from succinic anhydride (1) according to the reported method,^11*c*^ was treated with morpholine under microwave irradiation to give 3-(5-amino-1*H*-1,2,4-triazol-3-yl)propanamide 5a (Table 1). The optimization of conditions for the synthesis of 5a started with an attempt to perform the reaction of 2 with morpholine in ethanol under microwave irradiation at 180 °C for 25 min (Table 1, Entry 1). To our satisfaction, we successfully obtained the desired product 5a in high purity after simple filtration; however the yield was rather low (27%). The screening of solvents revealed that conducting the reaction in acetonitrile led to a better yield (Entry 4). Further optimising of the reaction conditions, we found that decreasing the reaction temperature to 170 °C led to yield improvements (Entry 5). Therefore, the satisfactory results were achieved when the synthesis of *N*-morpholino-substituted 3-(5-amino-1*H*-1,2,4-triazol-3-yl)propanamide (5a) was performed using reaction of 2 with morpholine in acetonitrile at 170 °C for 25 minutes.

**Table 1:** Optimization of conditions for the synthesis of *N*-morpholino-substituted 3-(5-amino-1*H*-1,2,4-triazol-3-yl)propanamide (5a)^a^

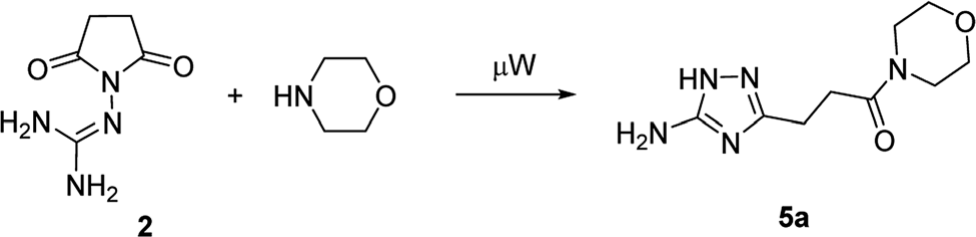				
Entry	Solvent	Temperature (°C)	Time (min)	Isolated yield (%)
1	EtOH	180	25	27
2	H_2_O	180	25	28
3	AcOEt	180	25	64
4	MeCN	180	25	75
5	MeCN	170	25	79
6	MeCN	160	25	65
7	MeCN	170	30	73
8	MeCN	170	20	73
9^b^	MeCN	170	25	68
10^c^	MeCN	170	25	66

a The reaction was performed using Discover SP CEM microwave synthesizer with 1 mmol of 2 and 2 mmol of morpholine in 1 mL of the specified solvent.

b 1 mmol of 2 and 3 mmol of morpholine were used for the reaction.

c 1 mmol of 2 and 1.5 mmol of morpholine were used for the reaction.

The optimized conditions for the preparation of 5a were successfully applied for the synthesis of 5b–i allowing the preparation of a diverse library of substituted amides of 3-(5-amino-1*H*-1,2,4-triazol-3-yl)propanoic acid (5a–i) in the 1 mmol scale (Table 2). Using these optimized conditions, we attempted to scale up the reaction from 1 mmol to 10 mmol. The synthesis of some products (5a, 5b, 5d, and 5i) was performed in the 10 mmol scale with similar results.

**Table 2:** Microwave-assisted synthesis of substituted amides of 3-(5-amino-1*H*-1,2,4-triazol-3-yl)propanoic acid (5a–j)^a^

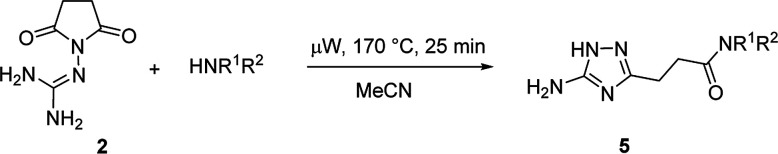
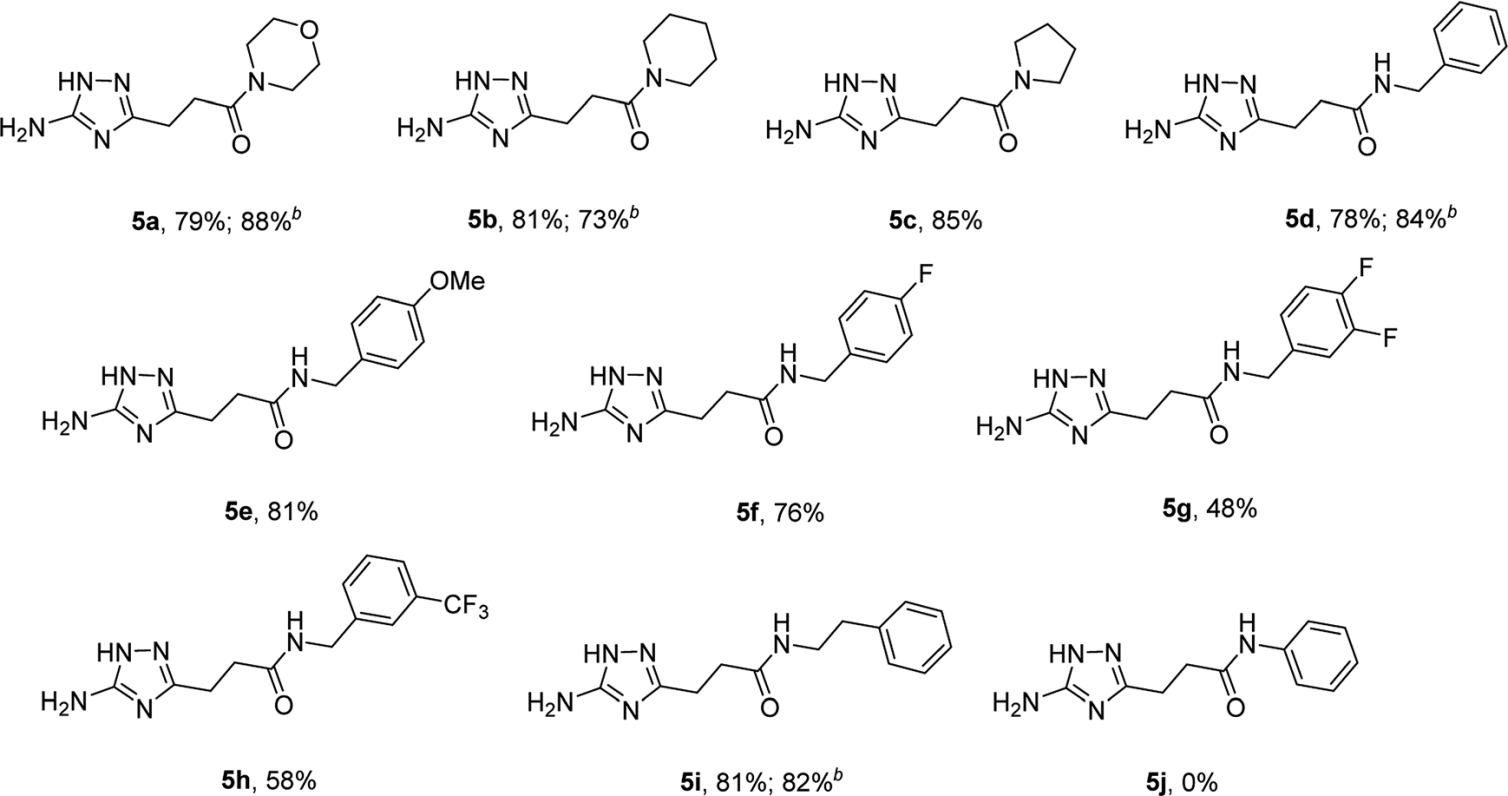

a The reactions were performed using a Discover SP CEM microwave synthesizer at 170 °C for 25 min with 1 mmol of *N*-guanidinosuccinimide (2) and 2 mmol of amine in 1 mL of acetonitrile.

b The reactions were scaled up to 10 mmol of *N*-guanidinosuccinimide (2) and 20 mmol of amine in 10 mL of acetonitrile.

However, when we attempted to further extend the reaction scope and involve aniline in the reaction with *N*-guanidinosuccinimide (2) under the optimized conditions, only starting material 2 was isolated. The analysis of the reaction mixture in the attempt to prepare 5j from 2 revealed the presence of aniline and unreacted 2 only. We propose that the nucleophilicity of aniline was not sufficient to initiate the ring opening of the cyclic imide and undergo the cascade of transformations.

### Synthesis of 3-(5-amino-1*H*-1,2,4-triazol-3-yl)propanamides (5) *via N*-arylsuccinimides (4)

To overcome this problem limiting the method scope, we designed an alternative pathway for the preparation of *N*-phenyl-substituted 3-(5-amino-1*H*-1,2,4-triazol-3-yl)propanamide (5j) *via* the reaction of *N*-phenylsuccinimide (4a) with aminoguanidine hydrochloride (Scheme 1, Pathway B). Aminoguanidine, being more nucleophilic, should be able to initiate the imide ring opening. Moreover, the guanidinium ion also might act as an acid catalyst increasing electrophilicity of the imide carbonyl group in this reaction. The neutralisation of the guanidinium ion on the intermediate in the second step supposed to facilitate the 1,2,4-triazole ring formation.

We attempted to carry out both steps in a one-pot fashion under microwave irradiation. First, *N*-phenylsuccinimide (4a) was heated with aminoguanidine hydrochloride in ethanol at 170 °C for 50 min. After cooling, an aqueous solution of potassium hydroxide was added to the reaction mixture and heating was continued at 180 °C for 15 min. We found that using non-aqueous solution of the same base dramatically increased the yield, while altering the solvent of the reaction had minimal effect on the outcome of the reaction (Table 3, Entries 2–5). Continuing optimization of the process using ethanol as the solvent, we observed that altering the reaction time before and after the addition of the base did not improve the outcome of the reaction (Entries 6–8). Unfortunately, further manipulations with the reaction temperature, time, type of base, or ratio of the reagents did not lead to any improvement in yields (*e.g.* Entries 9–11). These conditions were the most efficient for the preparation of 3-(5-amino-1*H*-1,2,4-triazol-3-yl)propananilide (5j), which was obtained using this one-pot process in 58% yield (Entry 3).

**Table 3:** Optimization of conditions for the synthesis of 3-(5-amino-1*H*-1,2,4-triazol-3-yl)propananilide (5j)^a^

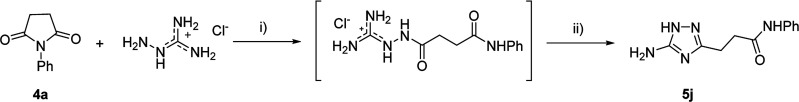			
Entry	Conditions		Isolated yield (%)
	(i)	(ii)	
1	170 °C, 50 min, EtOH	180 °C, 15 min, KOH in H_2_O	23
2	170 °C, 50 min, MeOH	180 °C, 15 min, KOH in MeOH	54
3	170 °C, 50 min, EtOH	180 °C, 15 min, KOH in EtOH	58
4	170 °C, 50 min, iPrOH	180 °C, 15 min, KOH in iPrOH	51
5	170 °C, 50 min, MeCN	180 °C, 15 min, KOH in EtOH	44
6	170 °C, 40 min, EtOH	180 °C, 15 min, KOH in EtOH	56
7	170 °C, 60 min, EtOH	180 °C, 15 min, KOH in EtOH	57
8	170 °C, 50 min, EtOH	180 °C, 20 min, KOH in EtOH	51
9	170 °C, 50 min, EtOH	170 °C, 30 min, KOH in EtOH	48
10	170 °C, 50 min, EtOH	180 °C, 15 min, NaOH in EtOH	55
11^b^	170 °C, 50 min, EtOH	180 °C, 15 min, KOH in EtOH	55

a The reactions were performed using a Discover SP CEM microwave synthesizer with 1 mmol of 4a and 1 mmol of aminoguanidine hydrochloride in 1 mL of the specified solvent in the first step and addition of 1.2 mmol of the base in the second step.

b 1.2 mmol of aminoguanidine hydrochloride and 1.4 mmol of KOH were used in the reaction.

**Scheme 2:**
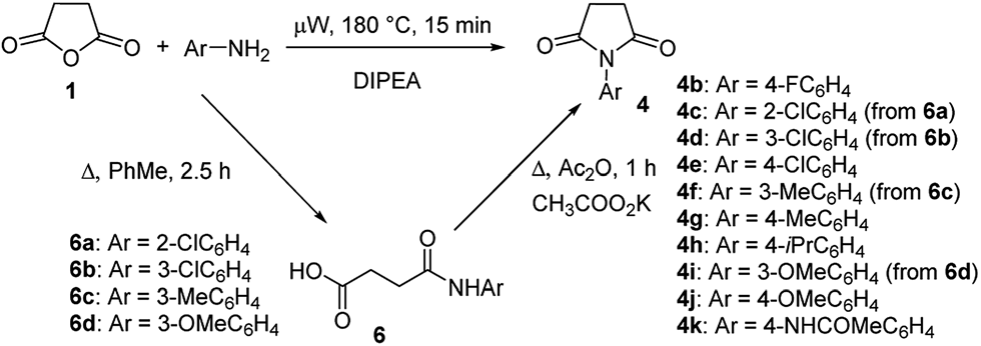
Synthesis of *N*-arylsuccinimides (4).

To explore the scope of this new method for the synthesis of other arylamides of 3-(5-amino-1*H*-1,2,4-triazol-3-yl)propanoic acid, we prepared a library of *N*-arylsuccinimides (4) from succinic anhydride (1) and anilines adopting the approach reported by Benjamin *et al.* (Scheme 2).^17^ However, this method failed when applied to *ortho*- and *meta*-substituted anilines and therefore imides 4c, 4d, 4f, and 4i were synthesized *via* intermediate *N*-arylsuccinamic acids (6).^18^ The *N*-arylsuccinimides (4) were then used as substrates in the reaction with aminoguanidine hydrochloride to prepare *N*-arylamides of 3-(5-amino-1*H*-1,2,4-triazol-3-yl)propanoic acid (5j–t) using the optimised conditions (Table 4). A wide range of substituents in various positions of the benzene ring of 4 were equally well tolerated in the reaction with aminoguanidine hydrochloride. In all cases, *N*-arylamides of 3-(5-amino-1*H*-1,2,4-triazol-3-yl)propanoic acid (5j–t) were isolated in high purity *via* simple filtration. The reaction was effectively performed in both 1 and 10 mmol scales with similar outcomes.

**Table 4:** Microwave-assisted synthesis of *N*-arylamides of 3-(5-amino-1*H*-1,2,4-triazol-3-yl)propanoic acid (5j–t)^a^

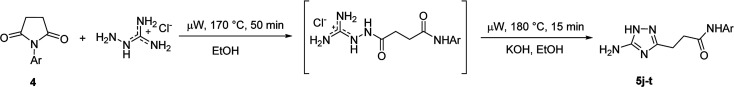				
Compound	Structure	Scale (mmol)	Yield (%)	Melting point (°C)
5j	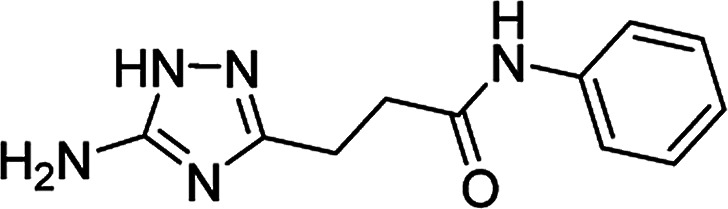	1	58	245–246^b^
		10	56	
5k	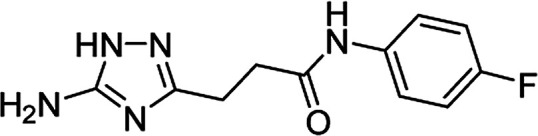	1	56	218–220^c^
		10	61	
5l	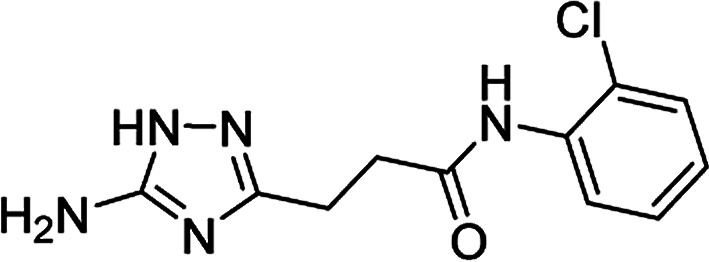	1	42	179–181^d^
		10	60	
5m	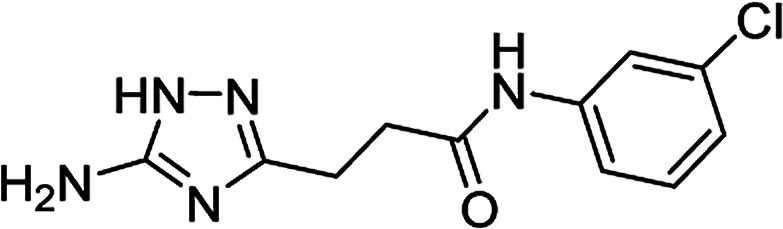	1	64	209–210^d^
		10	74	
5n	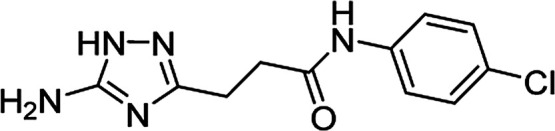	1	64	219–220^c^
		10	67	
5o	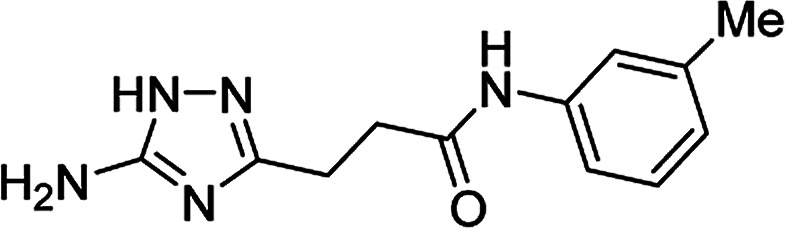	1	46	202–203^d^
		10	47	
5p	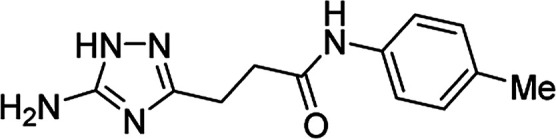	1	48	221^c^
		10	43	
5q	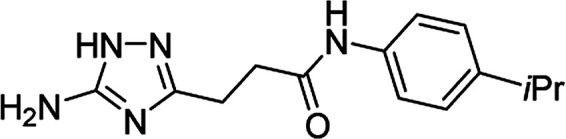	1	59	187–188^d^
		10	68	
5r	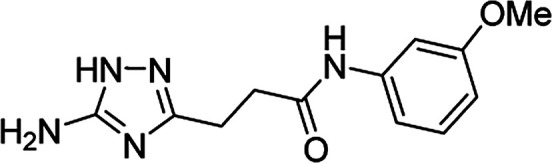	1	26	174–175^d^
		10	31	
5s	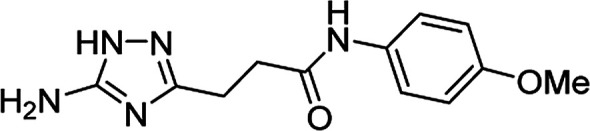	1	50	221–222^c^
		10	46	
5t	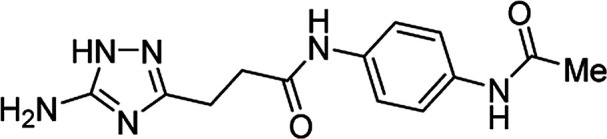	1	38	267–269^b^
		10	44	

a The reaction was performed using a Discover SP CEM microwave synthesizer at 170 °C for 50 min in EtOH followed by the addition of KOH in EtOH and heating at 180 °C for 15 min.

b Recrystallisation solvent: H_2_O.

c Recrystallisation solvent: MeOH.

d Recrystallisation solvent: MeCN.

### Annular prototropic tautomerisation of 3-(5-amino-1*H*-1,2,4-triazol-3-yl)propanamides (5) in solution

**Scheme 3:**
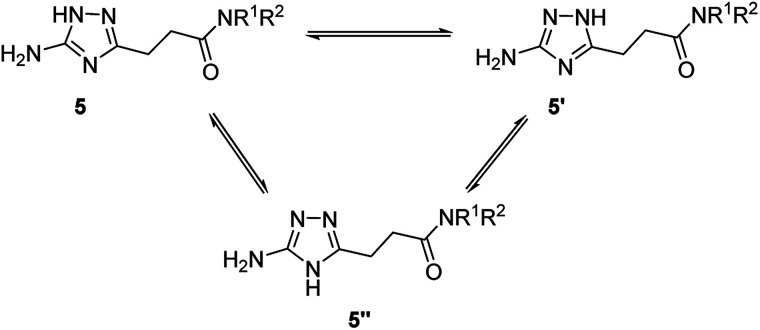
Theoretically possible annular tautomerism in 3-(5(3)-amino-1,2,4-triazol-3(5)-yl)propanamides.

Due to annular prototropic tautomerism, the prepared 5(3)-amino-1,2,4-triazoles may exist in three forms: 5-amino-1*H*-1,2,4-triazoles 5, 3-amino-1*H*-1,2,4-triazoles 5′, and 5-amino-4*H*-1,2,4-triazoles 5′′ (Scheme 3). We attempted to analyse tautomeric equilibria in the prepared triazoles using NMR spectroscopy.

In the ^13^C NMR spectra of the prepared compounds, the triazole ring signals appeared as two broad signals confirming its involvement in tautomerism. However, the tautomeric transformations were probably too fast to be detected by the ^13^C NMR spectroscopy under the experimental conditions and therefore tautomers were indistinguishable.

For majority of the *N*-alkyl-3-(5(3)-amino-1,2,4-triazol-3(5)-yl)propanamides (5a–5f and 5i), the separate signals of individual tautomers were not identifiable in their ^1^H NMR spectra. Nevertheless, ^1^H NMR spectra of some products gave two sets of signals for the primary amino group and the triazole proton. Using literature data,^11*e*,16^ these signals were attributed to tautomers 5 and 5′. The indicated signals were resolved in the spectra of compounds possessing at the amide group *N*-substituent with relatively higher electronegativity, *i.e.* anilides 5j–5t and two *N*-benzylamides with 3,4-difluoro- (5g) and 3-trifluoromethyl (5h) substituents. For these compounds (5g, 5h, 5j–5t), equilibrium constant (*K*_T_) and corresponding Gibbs free energy (Δ*G*_300_) values were estimated (Table 5). In the equilibria for these compounds, 5-amino-1*H*-1,2,4-triazoles 5, were found to be the predominant tautomers. Similarly to the earlier reported data,^11*e*,16*b*^ the higher the electron-withdrawing properties of a substituent at the carbon atom the triazole ring, the more equilibria were shifted towards 5-amino-1*H*-tautomers 5.

**Table 5:** Tautomeric composition of 3-(5(3)-amino-1*H*-1,2,4-triazol-3(5)-yl)propanamides^a^

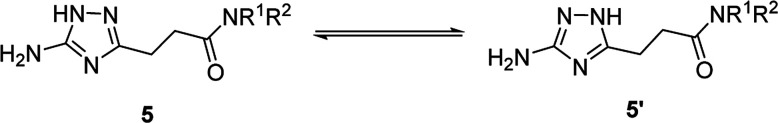						
Compounds	Signals of tautomers in ^1^H NMR spectra (ppm)				*K*_T_	−Δ*G*_300_ (kJ mol^−1^)
	NH_2_		NH (triazole)			
	5	5′	5	5′		
5g	5.76	5.07	11.58	12.30	3.0	2.7
5h	5.74	5.11	11.60	12.30	3.0	2.7
5j	5.77	5.04	11.57	12.32	3.2	2.9
5k	5.77	5.00	11.57	12.33	3.2	2.9
5l	5.81	5.00	11.60	12.34	3.3	3.0
5m	5.78	4.99	11.56	12.33	3.5	3.2
5n	5.78	4.98	11.56	12.32	3.3	3.0
5o	5.77	4.98	11.56	12.32	3.2	2.9
5p	5.77	5.03	11.56	12.31	3.0	2.7
5q	5.78	4.98	11.56	12.32	3.2	2.9
5r	5.76	5.07	11.58	12.30	3.3	3.0
5s	5.77	4.98	11.55	12.31	3.0	2.7
5t	5.77	4.97	11.55	12.32	3.2	2.9

a The NMR spectroscopy was performed at 27 °C (300 K) using DMSO-*d*_6_ as a solvent.

### X-ray crystallography

**Fig. 1:**
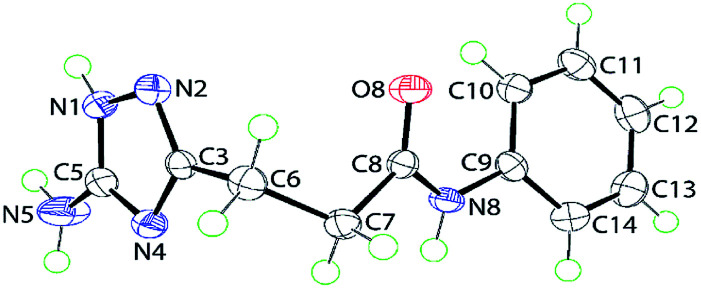
Molecular structure of 5j showing atom labelling scheme and 70% anisotropic displacement parameters.

The tautomerizable 1,2,4-triazoles with a primary amino on a carbon atom typically crystallize as 5-amino-forms with an annular hydrogen atom located at the side of the amino group.^19^ An example of two tautomers (5-amino- and 3-amino-forms) of 1*H*-1,2,4-triazoles appearing together in one crystal was also reported.^20^ To explore tautomeric preferences in the prepared compounds in solid state, the crystal and molecular structures of a representative compound 5j were determined by X-ray crystallography; the molecular structure is shown in Fig. 1. The key point of interest in the structure determination is the assignment of the tautomeric form for the triazole ring. The crystallographic analysis indicates the ring-H atom to be located on the N1 atom of the 5-amino-form (see Experimental). This assignment, *i.e.* 5-amino-1*H*-1,2,4-triazole, is confirmed in the distribution of bond lengths within the ring and in the nature of the supramolecular association in the crystal of 5j (see below). Thus, the C5–N1 bond length of 1.3359(15) Å is considerably longer than the C3–N2 bond of 1.3164(15) Å; the N1–N2 bond length is 1.3837(14) Å. Further, the C3–N4 and C5–N4 bond lengths vary systematically, *i.e.* 1.3681(15) Å is longer than 1.3351(15) Å. These observations are consistent with localisation of π-electron density in the C3–N2 and C5–N4 bonds. Overall, the molecule has the shape of the letter L as seen in the values of the dihedral angles formed between the central amide residue (r.m.s. deviation of the O8, N8, C7 and C8 atoms from the least-squares plane = 0.0084 Å) and the five- and six-membered rings of 79.54(4)° and 24.78(6)°, respectively, indicating, to a first approximation, a co-planar relationship between the amide and phenyl groups with the triazole ring lying perpendicular to this; the dihedral angle between the five- and six-membered rings is 86.65(4)°.

**Fig. 2:**
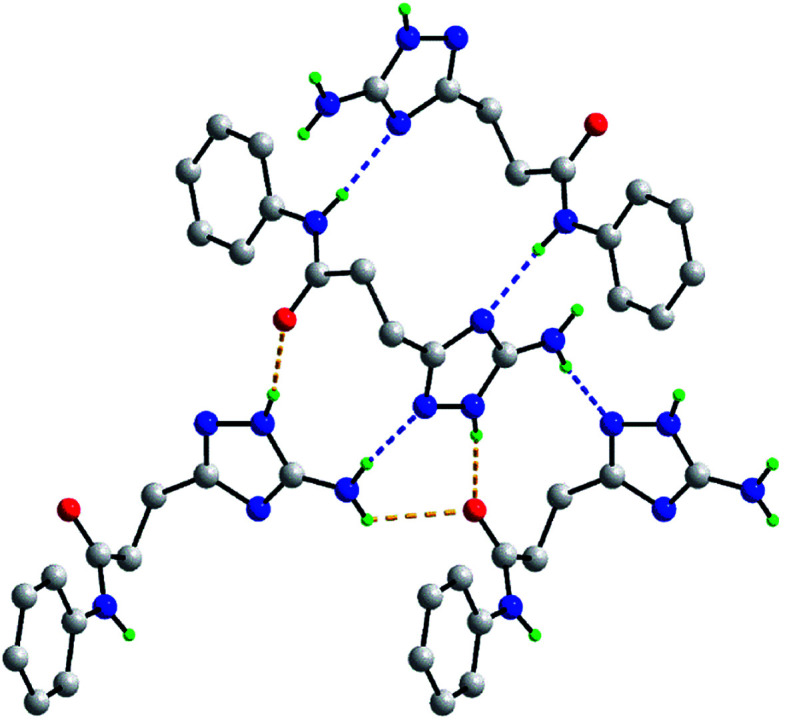
Hydrogen bonding between four molecules in the crystal of 5j. The N–H⋯O and N–H⋯N hydrogen bonding (see ESI Fig. S1†), are shown as orange and blue dashed lines, respectively. Non-participating hydrogen atoms have been omitted.

As mentioned above, the molecular packing in the crystal of 5j confirms the assignment of the tautomeric form of the five-membered ring. The crucial hydrogen bonding involving the triazole ring sees the formation of donor triazole-N1–H⋯O8 (carbonyl) and acceptor amine-N5–H⋯N2 (triazole) hydrogen bonds confirming protonation at the triazole-N1 atom at the amino group side. The second amine-N5–H atom forms a comparatively weaker hydrogen bond to the carbonyl-O8 atom to close a seven-membered {NH⋯O⋯HNH⋯N} supramolecular synthon; geometric details of the hydrogen bonding are given in the caption to ESI Fig. S1.† As shown in Fig. 2, three molecules are involved in the aforementioned hydrogen bonding scheme so that the seven-membered synthon is flanked on either side by 11-membered {NH⋯OC_4_N⋯HNC} synthons. Connections between the aforementioned aggregates are of the type amide-N8–H⋯N4 (triazole) which generate centrosymmetric, 14-membered {⋯NC_4_NH}_2_ synthons. The hydrogen scheme just described extends laterally to form a supramolecular layer parallel to (1 0 1), see ESI Fig. S1a.† The most obvious directional points of contact between layers to consolidate the three-dimensional molecular packing are of the type methylene-C7–H⋯π(C9–C14); a view of the unit cell contents is shown in ESI Fig. S1b.†

## Conclusion

In conclusion, we developed two complementing each other pathways for the synthesis of 3-(5-amino-1*H*-1,2,4-triazol-3-yl)propanamides (5) under microwave irradiation. Our methods were successfully applied for the preparation of a diverse library of twenty *N*-substituted 3-(5-amino-1*H*-1,2,4-triazol-3-yl)propanamides (5) in 1 and 10 mmol scales. Their tautomeric preferences were analysed using NMR spectroscopy and X-ray crystallography.

## Experimental section

### General information

Melting points (uncorrected) were determined on a Stuart™ SMP40 automatic melting point apparatus. ^1^H and ^13^C NMR spectra were recorded on a Bruker Fourier 300 spectrometer (300 MHz), using DMSO-*d*_6_ as a solvent and TMS as an internal reference. IR spectra were recorded on a Varian 640-IR FT-IR spectrometer using KBr mode. Microwave-assisted reactions were performed in closed vessel focused single mode using a CEM Discover SP microwave synthesizer (CEM, USA). The reaction temperatures were measured by an equipped IR sensor.

### General method for the microwave-assisted synthesis of 3-(5-amino-1*H*-1,2,4-triazol-3-yl)propanamides (5a-b, and 5d–i)

A mixture of *N*-guanidinosuccinimide (2) (156 mg, 1 mmol) and specified amine (2 mmol) in acetonitrile (1 mL) were irradiated in 10 mL seamless pressure vial using microwave system operating at maximal microwave power up to 300 W at 170 °C for 25 min. After cooling, the precipitated product was filtered, washed with acetonitrile and recrystalised from a suitable solvent. The reaction was also replicated in an increase scale of *N*-guanidinosuccinimide (2) (1.56 g, 10 mmol) and specified amine (20 mmol) in acetonitrile (10 mL).

#### 3-(5-Amino-1*H*-1,2,4-triazol-3-yl)-1-morpholinopropan-1-one (5a)

Yellowish solid, yield (1 mmol/10 mmol scale): 178 mg (79%)/1.97 g (88%), mp 166–167 °C (MeCN). ^1^H NMR (300 MHz, DMSO-*d*_6_): *δ* 2.65 (4H, br s, (CH_2_)_2_C=O), 3.41–3.44 (2H, m, (CH_2_)N), 3.53–3.54 (2H, m, (CH_2_)O), 5.58 (2H, br s, NH_2_), 11.79 (1H, br s, NH). ^13^C NMR (75 MHz, DMSO-*d*_6_): *δ* 23.1 (CH_2_CH_2_C=O), 30.1 (CH_2_CH_2_C=O), 41.4 (CH_2_N), 45.2 (CH_2_N), 65.98 ((CH_2_)_2_O), 158.2–158.6 (C-3 and C-5), 170.0 (C=O). IR (KBr) *ν* 3331 (N–H), 3192 (N–H), 1619 (C=O), 1542, 1476, 1255, 1115, 1064, 1013 cm^−1^. Anal. calcd for C_9_H_15_N_5_O_2_: C, 47.99; H, 6.71; N, 31.09. Found: C, 47.85; H, 6.77; N, 30.97.

#### 3-(5-Amino-1*H*-1,2,4-triazol-3-yl)-1-(piperidin-1-yl)propan-1-one (5b)

White solid, yield (1 mmol/10 mmol scale): 180 mg (81%)/1.62 g (73%), mp 165–167 °C (MeCN). ^1^H NMR (300 MHz, DMSO-*d*_6_): *δ* 1.42–1.60 (6H, m, (CH_2_)_3_), 2.62 (4H, s, (CH_2_)_2_C=O), 3.36–3.42 (4H, m, (CH_2_)_2_N), 5.53 (2H, br s, NH_2_), 11.69 (1H, br s, NH). ^13^C NMR (75 MHz, DMSO-*d*_6_): *δ* 23.2 (CH_2_CH_2_C=O), 24.0 (CH_2_), 25.2 (CH_2_), 25.9 (CH_2_), 30.4 (CH_2_CH_2_C=O), 41.9 (CH_2_N), 45.7 (CH_2_N), 158.5 (C-3 and C-5), 169.2 (C=O). IR (KBr) *ν* 3305 (N–H), 3138 (N–H), 1617 (C=O), 1479, 1405, 1287, 1135, 1098, 1067 cm^−1^. Anal. calcd for C_10_H_17_N_5_O: C, 53.79; H, 7.67; N, 31.37. Found: C, 53.67; H, 7.72; N, 31.24.

#### 3-(5-Amino-1*H*-1,2,4-triazol-3-yl)-*N*-(4-benzyl)propanamide (5d)

White solid, yield (1 mmol/10 mmol scale): 191 mg (78%)/2.05 g (84%), mp 208–210 °C (EtOH). ^1^H NMR (300 MHz, DMSO-*d*_6_): *δ* 2.45–2.49 (2H, m, CH_2_CH_2_C=O), 2.67 (2H, t, ^3^*J* = 7.7 Hz, CH_2_CH_2_C=O), 4.26 (2H, d, ^3^*J* = 5.9 Hz, CH_2_NH), 5.61 (2H, br s, NH_2_), 7.21–7.33 (5H, m, H-2′, H-3′, H-4′, H-5′and H-6′), 8.35 (1H, br t, ^3^*J* = 5.6 Hz, NHC=O), 11.67 (1H, br s, NH). ^13^C NMR (75 MHz, DMSO-*d*_6_): *δ* 23.6 (CH_2_CH_2_C=O), 33.4 (CH_2_CH_2_C=O), 41.9 (CH_2_NH), 126.5 (C-4′), 127.0 (C-2′ and C-6′), 128.1 (C-3′ and C-5′), 139.5 (C-1′), 158.3 (C-3 and C-5), 171.2 (C=O). IR (KBr) *ν* 3414 (N–H), 3324 (N–H), 3247 (N–H), 1638 (C=O), 1546, 1482, 1225, 1081, 1058, 1003 cm^−1^. Anal. calcd for C_12_H_15_N_5_O: C, 58.76; H, 6.16; N, 28.55. Found: C, 58.61; H, 6.30; N, 28.47.

#### 3-(5-Amino-1*H*-1,2,4-triazol-3-yl)-*N*-(4-methoxybenzyl)propanamide (5e)

White solid, yield: 224 mg (84%), mp 218–220 °C (EtOH). ^1^H NMR (300 MHz, DMSO-*d*_6_): *δ* 2.38–2.48 (2H, m, CH_2_CH_2_C=O), 2.65 (2H, t, ^3^*J* = 7.7 Hz, CH_2_CH_2_C=O), 3.72 (3H, s, OCH_3_), 4.19 (2H, d, ^3^*J* = 5.9 Hz, CH_2_NH), 5.62 (2H, br s, NH_2_), 6.87 (2H, d, ^3^*J* = 8.7 Hz, H-3′ and H-5′), 7.15 (2H, d, ^3^*J* = 8.7 Hz, H-2′ and H-6′), 8.27 (1H, br t, ^3^*J* = 5.7 Hz, NHC=O), 11.64 (1H, br s, NH). ^13^C NMR (75 MHz, DMSO-*d*_6_): *δ* 23.6 (CH_2_CH_2_C=O), 33.4 (CH_2_CH_2_C=O), 41.3 (CH_2_NH), 54.9 (OCH_3_), 113.6 (C-3′ and C-5′), 128.3 (C-2′ and C-6′), 131.4 (C-1′), 158.0 (C-4′), 159.5 (C-3 and C-5), 171.0 (C=O). IR (KBr) *ν* 3413 (N–H), 3324 (N–H), 3246 (N–H), 1637 (C=O), 1547, 1482, 1257, 1106, 1058, 1032 cm^−1^. Anal. calcd for C_13_H_17_N_5_O_2_: C, 56.71; H, 6.22; N, 25.44. Found: C, 56.58; H, 6.31; N, 25.32.

#### 3-(5-Amino-1*H*-1,2,4-triazol-3-yl)-*N*-(4-fluorobenzyl)propanamide (5f)

White solid, yield: 199 mg (76%), mp 218–219 °C (EtOH). ^1^H NMR (300 MHz, DMSO-*d*_6_): *δ* 2.41–2.51 (2H, m, CH_2_CH_2_C=O), 2.67 (2H, t, ^3^*J* = 7.6 Hz, CH_2_CH_2_C=O), 4.24 (2H, d, ^3^*J* = 5.9 Hz, CH_2_NH), 5.61 (2H, br s, NH_2_), 7.12 (2H, dd, ^3^*J*_HH_ = 8.9 Hz, ^3^*J*_HF_ = 8.9 Hz, H-3′ and H-5′), 7.25 (2H, dd, ^4^*J*_HF_ = 5.7 Hz, ^3^*J*_HH_ = 8.8 Hz, H-2′ and H-6′), 8.36 (1H, br t, ^3^*J* = 5.8 Hz, NHC=O), 11.69 (1H, br s, NH). ^13^C NMR (75 MHz, DMSO-*d*_6_): *δ* 23.6 (CH_2_CH_2_C=O), 33.4 (CH_2_CH_2_C=O), 41.2 (CH_2_NH), 114.8 (d, ^2^*J*_CF_ = 21.6 Hz, C-3′ and C-5′), 128.9 (d, ^3^*J*_CF_ = 8.20 Hz, C-2′ and C-6′), 135.7 (d, ^4^*J*_CF_ = 3.0 Hz, C-1′), 157.4 (C-3 and C-5), 161.0 (d, ^1^*J*_CF_ = 242.0 Hz, C-4′), 171.2 (C=O). IR (KBr) *ν* 3415 (N–H), 3325 (N–H), 3248 (N–H), 1639 (C=O), 1547, 1482, 1225, 1094, 1059, 1005 cm^−1^. Anal. calcd for C_12_H_14_FN_5_O: C, 54.75; H, 5.36; N, 26.60. Found: 54.68; H, 5.40; N, 26.54.

#### 3-(5-Amino-1*H*-1,2,4-triazol-3-yl)-*N*-(3,4-difluorobenzyl)propanamide (5g)

White solid, yield: 136 mg (48%), mp 173–175 °C (EtOH). ^1^H NMR (300 MHz, DMSO-*d*_6_): *δ* 2.47–2.68 (4H, m, CH_2_CH_2_C=O), 4.25 (2H, d, ^3^*J* = 5.9 Hz, CH_2_NH), 5.07–5.76 (2H, br s, NH_2_), 7.05–7.09 (1H, m, H-2′), 7.23–7.40 (2H, m, H-5′ and H-6′), 8.42 (1H, br t, ^3^*J* = 5.6 Hz, NHC=O), 11.58–12.30 (1H, br s, NH). ^13^C NMR (75 MHz, DMSO-*d*_6_): *δ* 24.0 (CH_2_CH_2_C=O), 33.3 (CH_2_CH_2_C=O), 41.0 (CH_2_NH), 115.9 (d, ^2^*J*_CF_ = 17.4 Hz, C-2′), 117.1 (d, ^2^*J*_CF_ = 17.0 Hz, C-5′), 123.6 (dd, ^4^*J*_CF_ = 3.1 Hz, ^3^*J*_CF_ = 7.0 Hz, C-6′), 137.5 (dd, ^4^*J*_CF_ = 3.8 Hz, ^3^*J*_CF_ = 5.5 Hz, C-1′), 148.2 (dd, ^2^*J*_CF_ = 12.5 Hz, ^1^*J*_CF_ = 243.8 Hz, C-4′), 149.1 (dd, ^2^*J*_CF_ = 12.7 Hz, ^1^*J*_CF_ = 245.1 Hz, C-3′), 156.7 and 159.8 (C-3 and C-5), 171.4 (C=O). IR (KBr) *ν* 3417 (N–H), 3322 (N–H), 3246 (N–H), 1636 (C=O), 1554, 1433, 1226, 1115, 1061, 1014 cm^−1^. Anal. calcd for C_12_H_13_F_2_N_5_O: C, 51.24; H, 4.66; N, 24.90. Found: C, 51.09; H, 4.72; N, 24.78.

#### 3-(5-Amino-1*H*-1,2,4-triazol-3-yl)-*N*-(3-trifluoromethylbenzyl)propanamide (5h)

White solid, yield: 181 mg (58%), mp 157–158 °C (EtOH). ^1^H NMR (300 MHz, DMSO-*d*_6_): *δ* 2.48–2.71 (4H, m, CH_2_CH_2_C=O), 4.36 (2H, d, ^3^*J* = 5.9 Hz, CH_2_NH), 5.11–5.74 (2H, br s, NH_2_), 7.54–7.59 (4H, m, H-2′, H-3′, H-5′ and H-6′), 8.49 (1H, br t, ^3^*J* = 5.5 Hz, NHC=O), 11.60–12.30 (1H, br s, NH). ^13^C NMR (75 MHz, DMSO-*d*_6_): *δ* 23.9 (CH_2_CH_2_C=O), 33.4 (CH_2_CH_2_C=O), 41.5 (CH_2_NH), 123.3 (q, ^3^*J*_CF_ = 3.7 Hz, C-4′), 123.5 (q, ^3^*J*_CF_ = 3.8 Hz, C-2′), 124.2 (q, ^1^*J*_CF_ = 272.2 Hz, CF_3_), 128.9 (q, ^2^*J*_CF_ = 31.4 Hz, C-3′), 129.3 (C-5′), 131.2 (C-6′), 141.1 (C-1′), 156.8 and 159.8 (C-3 and C-5), 171.5 (C=O). IR (KBr) *ν* 3410 (N–H), 3321 (N–H), 3232 (N–H), 1641 (C=O), 1553, 1480, 1260, 1167, 1115, 1071 cm^−1^. Anal. calcd for C_13_H_14_F_3_N_5_O: C, 49.84; H, 4.50; N, 22.36. Found: C, 49.79; H, 4.53; N, 22.29.

#### 3-(5-Amino-1*H*-1,2,4-triazol-3-yl)-*N*-(phenethyl)propanamide (5i)

White solid, yield (1 mmol/10 mmol scale): 211 mg (81%)/2.13 g (82%), mp 188–190 °C (EtOH). ^1^H NMR (300 MHz, DMSO-*d*_6_): *δ* 2.37 (2H, t, ^3^*J* = 7.9 Hz, CH_2_CH_2_C=O), 2.60 (2H, t, ^3^*J* = 7.8 Hz, CH_2_CH_2_C=O), 2.69 (2H, t, ^3^*J* = 7.4 Hz, CH_2_CH_2_NH), 3.25 (2H, q, ^3^*J* = 6.8 Hz, CH_2_CH_2_NH), 5.59 (2H, br s, NH_2_), 7.17–7.31 (5H, m, H-2′, H-3′, H-4′, H-5′and H-6′), 7.94 (1H, br t, ^3^*J* = 5.4 Hz, NHC=O), 11.68 (1H, br s, NH). ^13^C NMR (75 MHz, DMSO-*d*_6_): *δ* 23.6 (CH_2_CH_2_C=O), 33.4 (CH_2_CH_2_C=O), 35.1 (CH_2_CH_2_NH), 40.1 (CH_2_CH_2_NH), 125.9 (C-4′), 128.2 (C-2′ and C-6′), 128.5 (C-3′ and C-5′), 139.4 (C-1′), 158.5 (C-3 and C-5), 171.0 (C=O). IR (KBr) *ν* 3417 (N–H), 3325 (N–H), 3241 (N–H), 1640 (C=O), 1553, 1479, 1227, 1085, 1063, 1018 cm^−1^. Anal. calcd for C_14_H_17_N_5_O: C, 60.21; H, 6.61; N, 27.01. Found: C, 60.08; H, 6.70; N, 26.88.

### Microwave-assisted synthesis of 3-(5-amino-1*H*-1,2,4-triazol-3-yl)-1-(pyrrolidin-1-yl)propan-1-one (5c)

A mixture of *N*-guanidinosuccinimide (2) (156 mg, 1 mmol) and pyrrolidine (165 μL, 2 mmol) in acetonitrile (1 mL) were irradiated in 10 mL seamless pressure vial using microwave system operating at maximal microwave power up to 300 W at 170 °C for 25 min. After cooling, the solvent was evaporated under vacuum and the residue was triturated with ethyl acetate. The precipitate formed, was filtered, washed with ethyl acetate and recrystalised from ethanol to give desired product 5c. White solid; yield: 178 mg (85%); mp 189–191 °C (EtOH); ^1^H NMR (300 MHz, DMSO-*d*_6_): *δ* 1.75 (2H, m, ^3^*J* = 6.6 Hz, CH_2_), 1.86 (2H, m, ^3^*J* = 6.7 Hz, CH_2_), 2.53–2.64 (4H, m, (CH_2_)_2_C=O), 3.27 (2H, t, ^3^*J* = 6.8 Hz, CH_2_N), 3.39 (2H, t, ^3^*J* = 6.7 Hz, CH_2_N), 5.62 (2H, br s, NH_2_), 11.59 (1H, br s, NH); ^13^C NMR (75 MHz, DMSO-*d*_6_): *δ* 22.8 (CH_2_CH_2_C=O), 23.9 (CH_2_), 25.5 (CH_2_), 31.8 (CH_2_CH_2_C=O), 45.2 (CH_2_N), 45.7 (CH_2_N), 159.4 (C-3 and C-5), 169.4 (C=O). IR (KBr) *ν* 3358 (N–H), 3204 (N–H), 1622 (C=O), 1568, 1467, 1397, 1341, 1228, 1062 cm^−1^. Anal. calcd for C_9_H_15_N_5_O: C, 51.66; H, 7.23; N, 33.47. Found: C, 51.58; H, 7.25; N, 33.42.

### General method for the microwave-assisted synthesis of 3-(5-amino-1*H*-1,2,4-triazol-3-yl)propanamides (5j–t)

A mixture of the corresponding *N*-arylsuccnimide (4a–k) (1 mmol) and aminoguanidine hydrochloride (111 mg, 1 mmol) in ethanol (1 mL) was irradiated in the 10 mL seamless pressure vial using microwave system operating at maximal microwave power up to 300 W at 170 °C for 50 min. After cooling to the ambient temperature, 480 μL of KOH solution in ethanol (2.5 M) was added to the vial and the reaction mixture was irradiated again at 180 °C for 15 min. After cooling to the ambient temperature, the reaction mixture was diluted with 15 mL of water. The precipitated solid was filtered, washed with cold water and recrystalised from a suitable solvent to give desired products 5j–t. The reaction was also replicated in an increase scale of *N*-arylsuccnimide (4a–k) (10 mmol) and aminoguanidine hydrochloride (1.11 g, 10 mmol) in ethanol (10 mL).

#### 3-(5-Amino-1*H*-1,2,4-triazol-3-yl)-*N*-(phenyl)propanamide (5j)

Pale brownish solid, yield (1 mmol/10 mmol scale): 135 mg (58%)/1.30 g (56%), mp 245–246 °C (H_2_O). ^1^H NMR (300 MHz, DMSO-*d*_6_): *δ* 2.65–2.71 (4H, m, (CH_2_)_2_C=O), 5.04–5.77 (2H, br s, NH_2_), 7.01 (1H, t, ^3^*J* = 7.4 Hz, H-4′), 7.28 (2H, t, ^3^*J* = 7.9 Hz, H-3′ and H-5′), 7.59 (2H, d, ^3^*J* = 7.6 Hz, H-2′ and H-6′), 9.95 (1H, s, NHC=O), 11.57–12.32 (1H, br s, NH). ^13^C NMR (75 MHz, DMSO-*d*_6_): *δ* 23.8 (CH_2_CH_2_C=O), 34.3 (CH_2_CH_2_C=O), 118.9 (C-2′ and C-6′), 122.8 (C-4′), 128.5 (C-3′ and C-5′), 139.3 (C-1′), 156.7–159.8 (C-3 and C-5), 170.3 (C=O). IR (KBr) *ν* 3464 (N–H), 3298 (N–H), 3164 (N–H), 1649 (C=O), 1596, 1498, 1317, 1258, 1166, 1095 cm^−1^. Anal. calcd for C_11_H_13_N_5_O: C, 57.13; H, 5.67; N, 30.28. Found: C, 57.05; H, 5.82; N, 30.21.

#### 3-(5-Amino-1*H*-1,2,4-triazol-3-yl)-*N*-(4-fluorophenyl)propanamide (5k)

White solid, yield (1 mmol/10 mmol scale): 139 mg (56%)/1.51 g (61%), mp 218–220 °C (MeOH). ^1^H NMR (300 MHz, DMSO-*d*_6_): *δ* 2.64–2.70 (4H, m, (CH_2_)_2_C=O), 5.00–5.77 (2H, br s, NH_2_), 7.12 (2H, dd, ^3^*J*_HF_ = 8.9 Hz, ^3^*J*_HH_ = 8.9 Hz, H-3′and H-5′), 7.61 (2H, dd, ^3^*J*_HF_ = 5.1 Hz, ^3^*J*_HH_ = 9.1 Hz, H-2′ and H-6′), 10.00 (1H, s, NHC=O), 11.57–12.33 (1H, br s, NH). ^13^C NMR (75 MHz, DMSO-*d*_6_): *δ* 23.7 (CH_2_CH_2_C=O), 34.2 (CH_2_CH_2_C=O), 115.1 (d, ^2^*J*_CF_ = 21.6 Hz, C-2′ and C-6′), 120.6 (d, ^3^*J*_CF_ = 7.5 Hz, C-2′ and C-6′), 135.7 (d, ^4^*J*_CF_ = 2.2 Hz, C-1′), 157.7 (d, ^1^*J*_CF_ = 239.2 Hz, C-1′), 156.7 and 159.8 (C-3 and C-5), 170.2 (C=O). IR (KBr) *ν* 3417 (N–H), 3325 (N–H), 3256 (N–H), 1660 (C=O), 1624, 1546, 1404, 1223, 1098, 1068 cm^−1^. Anal. calcd for C_11_H_12_FN_5_O: C, 53.01; H, 4.85; N, 28.10. Found: C, 52.88; H, 4.96; N, 27.92.

#### 3-(5-Amino-1*H*-1,2,4-triazol-3-yl)-*N*-(2-chlorophenyl)propanamide (5l)

Yellowish solid, yield (1 mmol/10 mmol scale): 112 mg (42%)/1.59 g (60%), mp 179–181 °C (MeCN). ^1^H NMR (300 MHz, DMSO-*d*_6_): *δ* 2.71 (4H, m, (CH_2_)_2_C=O), 5.00–5.81 (2H, br s, NH_2_), 7.16 (1H, dt, ^4^*J* = 1.4 Hz, ^3^*J* = 7.7 Hz, H-4′), 7.31 (1H, dt, ^4^*J* = 1.4 Hz, ^3^*J* = 7.7 Hz, H-5′), 7.47 (1H, dd, ^4^*J* = 1.4 Hz, ^4^*J* = 8.0 Hz, H-6′), 7.74 (1H, d, ^3^*J* = 7.7 Hz, H-3′), 9.52 (1H, s, NHC=O), 11.60–12.34 (1H, br s, NH). ^13^C NMR (75 MHz, DMSO-*d*_6_): *δ* 24.0 (CH_2_CH_2_C=O), 34.0 (CH_2_CH_2_C=O), 125.9 (C-4′ and C-6′), 126.1 (C-5′), 127.2 (C-3′), 129.3 (C-2′), 135.0 (C-1′), 156.7 and 159.7 (C-3 and C-5), 170.9 (C=O). IR (KBr) *ν* 3409 (N–H), 3337 (N–H), 3223 (N–H), 1653 (C=O), 1606, 1539, 1450, 1290, 1101, 1077 cm^−1^. Anal. calcd for C_11_H_12_ClN_5_O: C, 49.73; H, 4.55; N, 26.36. Found: C, 49.67; H, 4.66; N, 26.22.

#### 3-(5-Amino-1*H*-1,2,4-triazol-3-yl)-*N*-(3-chlorophenyl)propanamide (5m)

White solid, yield (1 mmol/10 mmol scale): 169 mg (64%)/1.97 g (74%), mp 209–210 °C (MeCN). ^1^H NMR (300 MHz, DMSO-*d*_6_): *δ* 2.68 (4H, m, (CH_2_)_2_C=O), 4.99–5.78 (2H, br s, NH_2_), 7.07 (1H, ddd, ^4^*J* = 1.0 Hz, ^4^*J* = 2.1 Hz, ^3^*J* = 7.9 Hz, H-4′), 7.31 (1H, t, ^3^*J* = 8.1 Hz, H-5′), 7.43 (1H, ddd, ^4^*J* = 1.0 Hz, ^4^*J* = 1.9 Hz, ^3^*J* = 8.3 Hz, H-6′), 7.82 (1H, dd, ^4^*J* = 2.0 Hz, ^4^*J* = 2.0 Hz, H-3′), 10.15 (1H, s, NHC=O), 11.56–12.33 (1H, br s, NH). ^13^C NMR (75 MHz, DMSO-*d*_6_): *δ* 23.6 (CH_2_CH_2_C=O), 34.3 (CH_2_CH_2_C=O), 117.2 (C-6′), 118.3 (C-2′), 122.5 (C-4′), 130.2 (C-5′), 132.9 (C-3′), 140.7 (C-1′), 156.8 and 159.9 (C-3 and C-5), 170.8 (C=O). IR (KBr) *ν* 3417 (N–H), 3323 (N–H), 3243 (N–H), 1659 (C=O), 1625, 1597, 1481, 1286, 1097, 1068 cm^−1^. Anal. calcd for C_11_H_12_ClN_5_O: C, 49.73; H, 4.55; N, 26.36. Found: C, 49.62; H, 4.63; N, 26.28.

#### 3-(5-Amino-1*H*-1,2,4-triazol-3-yl)-*N*-(4-chlorophenyl)propanamide (5n)

White solid, yield (1 mmol/10 mmol scale): 171 mg (64%)/1.77 g (67%), mp 219–220 °C (MeOH). ^1^H NMR (300 MHz, DMSO-*d*_6_): *δ* 2.67 (4H, m, (CH_2_)_2_C=O), 4.98–5.78 (2H, br s, NH_2_), 7.33 (2H, d, ^3^*J* = 8.9 Hz, H-3′ and H-5′), 7.62 (2H, d, ^3^*J* = 8.9 Hz, H-2′ and H-6′), 10.10 (1H, s, NHC=O), 11.56–12.32 (1H, br s, NH). ^13^C NMR (75 MHz, DMSO-*d*_6_): *δ* 23.7 (CH_2_CH_2_C=O), 34.3 (CH_2_CH_2_C=O), 120.4 (C-2′ and C-6′), 126.3 (C-4′), 128.4 (C-3′ and C-5′), 138.2 (C-1′), 156.8 and 159.8 (C-3 and C-5), 170.6 (C=O). IR (KBr) *ν* 3416 (N–H), 3323 (N–H), 3244 (N–H), 1656 (C=O), 1625, 1542, 1480, 1240, 1098, 1068 cm^−1^. Anal. calcd for C_11_H_12_ClN_5_O: C, 49.73; H, 4.55; N, 26.36. Found: C, 49.66; H, 4.61; N, 26.27.

#### 3-(5-Amino-1*H*-1,2,4-triazol-3-yl)-*N*-(3-methylphenyl)propanamide (5o)

Yellowish solid, yield (1 mmol/10 mmol scale): 113 mg (46%)/1.16 g (47%), mp 202–203 °C (MeCN). ^1^H NMR (300 MHz, DMSO-*d*_6_): *δ* 2.26 (3H, s, CH_3_), 2.65 (4H, m, (CH_2_)_2_C=O), 4.98–5.77 (2H, br s, NH_2_), 6.83 (1H, d, ^3^*J* = 7.5 Hz, H-4′), 7.15 (1H, t, ^3^*J* = 7.8 Hz, H-5′), 7.36 (1H, d, ^3^*J* = 8.1 Hz, H-6′), 7.44 (1H, s, H-2′), 9.86 (1H, s, NHC=O), 11.56–12.32 (1H, br s, NH). ^13^C NMR (75 MHz, DMSO-*d*_6_): *δ* 21.1 (CH_3_), 23.8 (CH_2_CH_2_C=O), 34.3 (CH_2_CH_2_C=O), 116.1 (C-6′), 119.4 (C-2′), 123.5 (C-4′), 128.4 (C-5′), 137.7 (C-3′), 138.2 (C-1′), 156.7 and 159.8 (C-3 and C-5), 170.3 (C=O). IR (KBr) *ν* 3417 (N–H), 3323 (N–H), 3250 (N–H), 1660 (C=O), 1623, 1547, 1480, 1210, 1096, 1067 cm^−1^. Anal. calcd for C_12_H_15_N_5_O: C, 58.76; H, 6.16; N, 28.55. Found: C, 58.69; H, 6.22; N, 28.48.

#### 3-(5-Amino-1*H*-1,2,4-triazol-3-yl)-*N*-(4-methylphenyl)propanamide (5p)

White solid, yield (1 mmol/10 mmol scale): 117 mg (48%)/1.05 g (43%), mp 221 °C (MeOH). ^1^H NMR (300 MHz, DMSO-*d*_6_): *δ* 2.24 (3H, s, CH_3_), 2.63–2.70 (4H, m, (CH_2_)_2_C=O), 5.03–5.77 (2H, br s, NH_2_), 7.08 (2H, d, ^3^*J* = 8.2 Hz, H-3′ and H-5′), 7.47 (2H, d, ^3^*J* = 8.4 Hz, H-2′ and H-6′), 9.86 (1H, s, NHC=O), 11.56–12.31 (1H, br s, NH). ^13^C NMR (75 MHz, DMSO-*d*_6_): *δ* 20.3 (CH_3_), 23.9 (CH_2_CH_2_C=O), 34.2 (CH_2_CH_2_C=O), 118.9 (C-2′ and C-6′), 128.9 (C-3′ and C-5′), 131.6 (C-4′), 136.8 (C-1′), 156.6 and 159.8 (C-3 and C-5), 170.1 (C=O). IR (KBr) *ν* 3306 (N–H), 3192 (N–H), 3130 (N–H), 1663 (C=O), 1608, 1553, 1406, 1236, 1110, 1060 cm^−1^. Anal. calcd for C_12_H_15_N_5_O: C, 58.76; H, 6.16; N, 28.55. Found: C, 58.68; H, 6.20; N, 28.48.

#### 3-(5-Amino-1*H*-1,2,4-triazol-3-yl)-*N*-(4-isopropylphenyl)propanamide (5q)

White solid, yield (1 mmol/10 mmol scale): 162 mg (59%)/1.86 g (68%), mp 187–188 °C (MeCN). ^1^H NMR (300 MHz, DMSO-*d*_6_): *δ* 1.17 (6H, d, ^3^*J* = 6.9 Hz, (CH_3_)_2_CH), 2.64–2.67 (4H, m, (CH_2_)_2_C=O), 2.82 (1H, m, ^3^*J* = 6.9 Hz, (CH_3_)_2_CH), 4.98–5.78 (2H, br s, NH_2_), 7.14 (2H, d, ^3^*J* = 8.5 Hz, H-3′ and H-5′), 7.49 (2H, d, ^3^*J* = 8.5 Hz, H-2′ and H-6′), 9.86 (1H, s, NHC=O), 11.56–12.32 (1H, br s, NH). ^13^C NMR (75 MHz, DMSO-*d*_6_): *δ* 23.9 ((CH_3_)_2_CH), 23.9 (CH_2_CH_2_C=O), 32.7 ((CH_3_)_2_CH), 34.4 (CH_2_CH_2_C=O), 119.0 (C-2′ and C-6′), 126.2 (C-3′ and C-5′), 137.0 (C-1′), 142.8 (C-4′), 156.6 and 159.8 (C-3 and C-5), 170.2 (C=O). IR (KBr) *ν* 3429 (N–H), 3306 (N–H), 3243 (N–H), 1651 (C=O), 1594, 1532, 1414, 1251, 1093, 1065 cm^−1^. Anal. calcd for C_14_H_19_N_5_O: C, 61.52; H, 7.01; N, 25.62. Found: C, 61.40; H, 7.08; N, 25.54.

#### 3-(5-Amino-1*H*-1,2,4-triazol-3-yl)-*N*-(3-methoxyphenyl)propanamide (5r)

Brownish solid, yield (1 mmol/10 mmol scale): 67 mg (26%)/812 mg (31%), mp 174–175 °C (MeCN). ^1^H NMR (300 MHz, DMSO-*d*_6_): *δ* 2.65–2.70 (4H, m, (CH_2_)_2_C=O), 3.34 (3H, s, OCH_3_), 5.07–5.76 (2H, br s, NH_2_), 6.60 (1H, ddd, ^4^*J* = 1.1 Hz, ^4^*J* = 2.5 Hz, ^3^*J* = 8.0 Hz, H-6′), 7.11 (1H, ddd, ^4^*J* = 1.5 Hz, ^4^*J* = 1.5 Hz, ^3^*J* = 8.3 Hz, H-4′), 7.18 (1H, t, ^3^*J* = 8.0 Hz, H-5′), 7.32 (1H, dd, ^4^*J* = 2.0 Hz, ^4^*J* = 2.0 Hz, H-2′), 9.94 (1H, s, NHC=O), 11.58–12.30 (1H, br s, NH). ^13^C NMR (75 MHz, DMSO-*d*_6_): *δ* 23.6 (CH_2_CH_2_C=O), 34.3 (CH_2_CH_2_C=O), 54.8 (OCH_3_), 104.7 (C-2′), 108.3 (C-4′), 111.2 (C-6′), 129.3 (C-5′), 140.4 (C-1′), 156.8 and 159.4 (C-3 and C-5), 159.4 (C-3′), 170.4 (C=O). IR (KBr) *ν* 3438 (N–H), 3304 (N–H), 3223 (N–H), 1670 (C=O), 1612, 1548, 1478, 1211, 1074, 1045 cm^−1^. Anal. calcd for C_12_H_15_N_5_O_2_: C, 55.16; H, 5.79; N, 26.80. Found: C, 55.04; H, 5.86; N, 26.68.

#### 3-(5-Amino-1*H*-1,2,4-triazol-3-yl)-*N*-(4-methoxyphenyl)propanamide (5s)

White solid, yield (1 mmol/10 mmol scale): 131 mg (50%)/1.19 g (46%), mp 221–222 °C (MeOH). ^1^H NMR (300 MHz, DMSO-*d*_6_): *δ* 2.62–2.67 (4H, m, (CH_2_)_2_C=O), 3.71 (3H, s, OCH_3_), 4.98–5.77 (2H, br s, NH_2_), 6.85 (2H, d, ^3^*J* = 9.1 Hz, H-3′ and H-5′), 7.49 (2H, d, ^3^*J* = 9.0 Hz, H-2′ and H-6′), 9.80 (1H, s, NHC=O), 11.55–12.31 (1H, br s, NH). ^13^C NMR (75 MHz, DMSO-*d*_6_): *δ* 23.9 (CH_2_CH_2_C=O), 33.7 (CH_2_CH_2_C=O), 55.0 (OCH_3_), 113.7 (C-3′ and C-5′), 120.4 (C-2′ and C-6′),132.5 (C-1′), 154.9 (C-4′), 156.6 and 159.8 (C-3 and C-5), 169.8 (C=O). IR (KBr) *ν* 3416 (N–H), 3295 (N–H), 3135 (N–H), 1652 (C=O), 1548, 1515, 1407, 1249, 1067, 1029 cm^−1^. Anal. calcd for C_12_H_15_N_5_O_2_: C, 55.16; H, 5.79; N, 26.80. Found: C, 55.05; H, 5.82; N, 26.73.

#### 3-(5-Amino-1*H*-1,2,4-triazol-3-yl)-*N*-(4-acetamidophenyl)propanamide (5t)

Light grey solid, yield (1 mmol/10 mmol scale): 110 mg (38%)/1.28 g (44%), mp 267–269 °C (H_2_O). ^1^H NMR (300 MHz, DMSO-*d*_6_): *δ* 2.01 (3H, s, CH_3_C=O), 2.63–2.68 (4H, m, (CH_2_)_2_C=O), 4.97–5.77 (2H, br s, NH_2_), 7.44–7.51 (4H, m, H-2′, H-3′, H-5′, H-6′), 9.82 (1H, s, NHC=O), 9.88 (1H, s, NHC=O), 11.55–12.32 (1H, br s, NH). ^13^C NMR (75 MHz, DMSO-*d*_6_): *δ* 23.8 (CH_2_CH_2_C=O), 33.7 (CH_2_CH_2_C=O), 119.2 (C-2′, C-3′, C-5′ and C-6′), 134.5 (C-4′), 134.5 (C-1′), 156.7 and 159.8 (C-3 and C-5), 167.8 (C=OCH_3_), 170.0 (C=O). IR (KBr) *ν* 3463 (N–H), 3306 (N–H), 3117 (N–H), 3030 (N–H), 1662 (C=O), 1617, 1533, 1408, 1277, 1120, 1058 cm^−1^. Anal. calcd for C_13_H_16_N_6_O_2_: C, 54.16; H, 5.59; N, 29.15. Found: C, 54.02; H, 5.64; N, 29.00.

### X-Ray crystallographic analysis

Intensity data for 5j were measured for a colourless crystal (0.09 × 0.13 × 0.24 mm) at 100 K on an Rigaku/Oxford Diffraction XtaLAB Synergy diffractometer (Dualflex, AtlasS2) fitted with CuKα radiation (*λ* = 1.54178 Å) so that *θ*_max_ = 67.1°. Data reduction and Gaussian absorption corrections were by standard methods.^21^ The structure was solved by direct-methods^22^ and refined on *F*^2^ (ref. 23) with anisotropic displacement parameters, C-bound H atoms included in the riding model approximation and N-bound H atoms refined with N–H = 0.88–0.91 ± 0.01 Å. A weighting scheme of the form *w* = 1/[*σ*^2^(*F*_o_^2^) + (0.043*P*)^2^ + 0.479*P*] where *P* = (*F*_o_^2^ + 2*F*_c_^2^)/3) was introduced. The molecular structure diagram showing 70% probability displacement ellipsoids was generated by ORTEP for Windows^24^ and the packing diagrams with DIAMOND.^25^ Additional data analysis was made with PLATON.^26^ In order to confirm the location of the acidic-H atoms, an unrestrained refinement was performed which yielded N–H bond lengths in the range 0.84(2) to 0.898(18) Å, thereby, conforming the original assignment.

#### Crystal data for 3-(5-amino-1*H*-1,2,4-triazol-3-yl)-*N*-(phenyl)propanamide (5j)

*M* = 231.26, monoclinic, *P*2_1_/*n*, *a* = 8.58760(10), *b* = 9.09790(10), *c* = 14.20640(10) Å, *β* = 100.8930(10)°, *V* = 1089.934(19) Å^3^, *Z* = 4, *D*_x_ = 1.409 g cm^−3^, *F*(000) = 488, *μ* = 0.795 mm^−1^, no. reflns meas. = 25 310, no. unique reflns = 1939 (*R*_int_ = 0.028), no. reflns with *I* ≥ 2*σ*(*I*) = 1882, no. parameters = 166, *R* (obs. data) = 0.032, w*R*_2_ (all data) = 0.081. CCDC deposition number: 1844791.

## Conflicts of interest

There are no conflicts to declare.

## Supplementary Material

RA-008-C8RA04576C-s001

RA-008-C8RA04576C-s002
