# Supplementary material for: Synthesis of 3-(5-amino-1H-1,2,4-triazol-3-yl)propanamides and their tautomerism
Source: RSC Adv. 2018 Jun 19;8(40):22351–60. doi: 10.1039/c8ra04576c (PMC9081160; doi:10.1039/c8ra04576c)
Supplement: RA-008-C8RA04576C-s001 [file RA-008-C8RA04576C-s001.pdf]

Electronic Supplementary Material (ESI) for RSC Advances.
This journal is © The Royal Society of Chemistry 2018

Synthesis of 3-(5-amino-1*H*-1,2,4-triazol-3-yl)propanamides and their tautomerism

(Electronic Supplementary Information)

Felicia Phei Lin Lim,[a] Lin Yuing Tan,[a] Edward R. T. Tiekink,[b] Anton V. Dolzhenko[a,c]*

[a] School of Pharmacy, Monash University Malaysia, Jalan Lagoon Selatan, Bandar Sunway, Selangor Darul Ehsan 47500, Malaysia, anton.dolzhenko@monash.edu

[b] Research Centre for Crystalline Materials, School of Science and Technology, Sunway University, Bandar Sunway, Selangor Darul Ehsan 47500, Malaysia

[c] School of Pharmacy and Biomedical Sciences, Curtin Health Innovation Research Institute, Faculty of Health Sciences, Curtin University, GPO Box U1987 Perth, Western Australia 6845, Australia, anton.dolzhenko@curtin.edu.au

Table of contents

Synthesis of starting materials (Experimental) S3

^{1}H and ^{13}C NMR spectra of succinamic acids **6** S9

^{1}H and ^{13}C NMR spectra of succimimides **2** and **4** S18

^{1}H and ^{13}C NMR spectra of 3-(5-amino-1*H*-1,2,4-triazol-3-yl)propanamides **5** S43

X-ray crystallography: packing and interactions in the crystals of **5j** S85

Synthesis of starting materials (Experimental)

General

Melting points (uncorrected) were determined on a Stuart™ SMP40 automatic melting point apparatus. ^{1}H and ^{13}C NMR spectra were recorded on a Bruker Fourier 300 spectrometer (300 MHz), using DMSO-d_6 as a solvent and TMS as an internal reference. IR spectra were recorded on a Varian 640-IR FT-IR spectrometer using KBr mode. Microwave-assisted reactions were performed in closed vessel focused single mode using a CEM Discover SP microwave synthesizer (CEM, USA). The reaction temperature was measured by an equipped IR sensor.

Synthesis of *N*-guanidinosuccinimide (2)

A mixture of aminoguanidine hydrochloride (6.6 g, 60 mmol) and succinic anhydride (6.6 g, 67 mmol) was heated in an oil bath until all solids melted. Reaction mixture was continuously stirred with a glass rod until the molten mixture solidified. Reaction mixture was heated for another 30 min. Solidified reaction mixture was then cooled to room temperature, followed by the addition of 10 mL of water and 27 mL of ethanol. The resultant mixture was refrigerated and the deposited solid was filtered and washed with ethanol. The crude solid was added to aqueous solution of sodium bicarbonate (3.5 M, 7 mL) and stirred for 20 min at room temperature. The product was filtered and washed with cold water and recrystallised from acetonitrile.

White solid; yield: 3.2 g (88%); mp > 350 °C (MeCN), lit.[1] > 300 °C.

^{1}H NMR (300 MHz, DMSO-d_6): δ 2.50 (4H, s, CH_2CH_2), 5.26 (2H, br s, NH_2), 5.60 (2H, br s, NH_2).

^{13}C NMR (75 MHz, DMSO-d_6): δ 26.7 (CH_2CH_2), 159.8 ($N{=}C(NH_2)_2$), 175.4 (2 x C=O).

***N*-arylsuccinimides 4b, 4e, 4g, 4j, and 4k); General Procedure**

A mixture of substituted aniline (2 mmol), succinic anhydride (300 mg, 3 mmol) and *N*,*N*-diisopropylethylamine (70 µL, 0.4 mmol) in tetrahydrofuran (1 mL) was irradiated in 10 mL seamless pressure vial using microwave system operating at maximal microwave power up to 300 W at 180 °C for 15 min. After cooling, the product was filtered and washed with tetrahydrofuran. Analytical sample was recrystallised from a suitable solvent.

***N*-(4-fluorophenyl)succinimide (4b)**

White solid; yield: 217 mg (56%); mp 172-173 °C (MeOH), lit.[2] 175-177 °C.

^{1}H NMR (300 MHz, DMSO-d_6): δ 2.77 (4H, s, CH_2CH_2), 7.31-7.33 (4H, m, H-2', H-3', H-5' and H-6').

^{13}C NMR (75 MHz, DMSO-d_6): δ 28.4 (CH_2CH_2), 115.6 (d, $^{2}J_{CF}$ = 22.9 Hz, C-3' and C-5'), 128.9 (d, $^{4}J_{CF}$ = 3.0 Hz, C-1'), 129.2 (d, $^{3}J_{CF}$ = 8.9 Hz, C-2' and C-6'), 161.3 (d, $^{1}J_{CF}$ = 245.0 Hz, C-1'), 176.8 (2 x C=O).

N-(4-chlorophenyl)succinimide (4e)

White solid; yield: 210 mg (50%); mp 163-165 °C (MeOH), lit.[3] 170 °C.

^{1}H NMR (300 MHz, DMSO-d_6): δ 2.78 (4H, s, CH_2CH_2), 7.31 (2H, d, ^3J = 8.8 Hz, H-2' and H-6'), 7.56 (2H, d, ^3J = 8.9 Hz, H-3' and H-5').

^{13}C NMR (75 MHz, DMSO-d_6): δ 28.4 (CH_2CH_2), 128.7 (C-2' and C-6'), 128.8 (C-3' and C-5'), 131.5 (C-1'), 132.5 (C-4'), 176.6 (2 x C=O).

N-(4-methylphenyl)succinimide (4g)

White solid; yield: 127 mg (34%); mp 151-152 °C (THF), lit.[3] 154-155 °C.

^{1}H NMR (300 MHz, DMSO-d_6): δ 2.34 (3H, s, CH_3), 2.76 (4H, s, CH_2CH_2), 7.12 (2H, d, ^3J = 8.4 Hz, H-2' and H-6'), 7.28 (2H, d, ^3J = 8.3 Hz, H-3' and H-5').

^{13}C NMR (75 MHz, DMSO-d_6): δ 20.6 (CH_3), 28.3 (CH_2CH_2), 126.8 (C-2' and C-6'), 129.2 (C-3' and C-5'), 130.0 (C-1'), 137.5 (C-4'), 176.9 (2 x C=O).

N-(4-methoxyphenyl)succinimide (4j)

Purple solid; yield: 328 mg (80%); mp 163-165 °C (MeOH), lit.[4] 165-167 °C.

^{1}H NMR (300 MHz, DMSO-d_6): δ 2.75 (4H, s, CH_2CH_2), 3.77 (3H, s, OCH_3), 7.02 (2H, d, ^3J = 9.1 Hz, H-2' and H-6'), 7.16 (2H, d, ^3J = 9.1 Hz, H-3' and H-5').

^{13}C NMR (75 MHz, DMSO-d_6): δ 28.3 (CH_2CH_2), 55.2 (OCH_3), 114.0 (C-3' and C-5'), 125.2 (C-1'), 128.2 (C-2' and C-6'), 158.7 (C-4'), 177.0 (2 x C=O).

N-(4-(N'-acetamido)phenyl)succinimide (4k)

Brown solid; yield: 446 mg (96%); mp 254-255 °C (MeOH).

^{1}H NMR (300 MHz, DMSO-d_6): δ 2.06 (3H, s, CH_3), 2.76 (4H, s, CH_2CH_2), 7.17 (2H, d, ^3J = 8.4 Hz, H-2' and H-6'), 7.66 (2H, d, ^3J = 8.6 Hz, H-3' and H-5'), 10.07 (1H, br s, NH).

^{13}C NMR (75 MHz, DMSO-d_6): δ 23.9 (CH_3), 28.3 (CH_2CH_2), 119.0 (C-3' and C-5'), 127.3 (C-1', C-2' and C-6'), 138.9 (C-4'), 168.4 (C=O), 176.9 (2 x C=O).

Synthesis of *N*-(4-isopropylphenyl)succinimide (4h)

A mixture of 4-isopropylyaniline (1.37 g, 10 mmol), succinic anhydride (1.50 g, 15 mmol) and N,N-diisopropylethylamine (348 µL, 2 mmol) in tetrahydrofuran (10 mL) was irradiated in 30 mL seamless pressure vial using microwave system operating at maximal microwave power up to 300 W at 180 °C for 15 min. After cooling, the solvent was evaporated under vacuum. The resultant residue was mixed with aqueous solution of sodium bicarbonate (1.2 M, 10 mL) and stirred for 10 min at room temperature. The precipitate was filtered and washed with cold water. Analytical sample was recrystallised from aqueous methanol.

White solid; yield: 1.5 g (70%); mp 127-129 °C (MeOH/H_2O).

^{1}H NMR (300 MHz, DMSO-d_6): δ 1.22 (6H, d, ^3J = 6.9 Hz, CH_3CH_3), 2.77 (4H, s, CH_2CH_2), 2.93 (1H, m, ^3J = 6.9 Hz, CH), 7.16 (2H, d, ^3J = 8.4 Hz, H-2' and H-6'), 7.34 (2H, d, ^3J = 8.3 Hz, H-3' and H-5').

^{13}C NMR (75 MHz, DMSO-d_6): δ 23.7 (CH_3CH_3), 28.3 (CH_2CH_2), 33.1 (CH), 126.6 (C-3' and C-5'), 126.9 (C-2' and C-6'), 130.3 (C-1'), 148.3 (C-4'), 176.9 (2 x C=O).

***N*-Arylsuccinamic acids 6; General Procedure**

A mixture of arylamine (20 mmol) and succinic anhydride (2.40 g, 24 mmol) were heated under reflux in 25 mL of toluene for 2.5 h. After cooling, the precipitate was filtered and washed with toluene and hexane. Analytical sample was recrystallised from toluene.

***N*-(2-chlorophenyl)succinamic acid (6a)**

White solid; yield: 4.49 g (99%); mp 145-146 °C (PhMe), lit.[5] 145-148 °C.

^{1}H NMR (300 MHz, DMSO-d_6): δ 2.51-2.53 (2H, m, CH_2CONH), 2.61-2.65 (2H, br t, CH_2COOH), 7.17 (1H, dt, ^4J = 1.8 Hz, ^3J = 7.7 Hz, H-4'), 7.31 (1H, dt, ^4J = 1.4 Hz, ^3J = 7.7 Hz, H-5'), 7.47 (1H, dd, ^4J = 1.5 Hz, ^3J = 8.0 Hz, H-6'), 7.71 (1H, dd, ^4J = 1.4 Hz, ^3J = 8.1 Hz, H-3').

^{13}C NMR (75 MHz, DMSO-d_6): δ 28.9 (CH_2), 30.5 (CH_2), 125.9-126.1 (C-2', C-4' and C-6'), 127.2 (C-5'), 129.3 (C-3'), 134.9 (C-1'), 170.4 (NHCO), 173.6 (COOH).

***N*-(3-chlorophenyl)succinamic acid (6b)**

White powder; yield: 4.05 g (89%); mp 111-113 °C (PhMe).

^{1}H NMR (300 MHz, DMSO-d_6): δ 2.54-2.51 (4H, m, CH_2CH_2), 7.07 (1H, ddd, ^4J = 1.0 Hz, ^4J = 2.1 Hz, ^3J = 7.9 Hz, H-4'), 7.31 (1H, t, ^3J = 8.1 Hz, H-5'), 7.42 (1H, ddd, $^4J'$= 1.1 Hz, ^4J = 1.9 Hz, ^3J = 8.2 Hz, H-6'), 7.80 (1H, dd, ^4J = 2.0 Hz, ^4J = 2.0 Hz, H-2').

^{13}C NMR (75 MHz, DMSO-d_6): δ 28.6 (CH_2), 31.0 (CH_2), 117.1 (C-6'), 118.3 (C-2'), 122.5 (C-4'), 130.3 (C-5'), 132.9 (C-3'), 140.6 (C-1'), 170.4 (NHCO), 173.7 (COOH).

N-(3-methylphenyl)succinamic acid (6c)

White solid; yield: 4.14 g (99%); mp 134-136 °C (PhMe), lit.[5] 136-140 °C.

^{1}H NMR (300 MHz, DMSO-d_6): δ 2.26 (3H, s, CH_3), 2.49-2.57 (4H, m, CH_2CH_2), 6.83 (1H, d, ^3J = 7.5 Hz, H-4’), 7.15 (1H, t, ^3J = 7.8 Hz, H-5’), 7.35 (1H, d, ^3J = 7.4 Hz, H-6’), 7.43 (1H, s, H-2’).

^{13}C NMR (75 MHz, DMSO-d_6): δ 21.1 (CH_3), 28.7 (CH_2), 30.9 (CH_2), 116.0 (C-6’), 119.4 (C-2’), 123.5 (C-4’), 128.4 (C-5’), 137.7 (C-3’), 139.1 (C-1’), 169.9 (NHCO), 173.7 (COOH).

N-(3-methoxyphenyl)succinamic acid (6d)

White solid; yield: 3.48 g (78%); mp 106-107 °C (PhMe).

^{1}H NMR (300 MHz, DMSO-d_6): δ 2.48-2.58 (4H, m, CH_2CH_2), 3.72 (3H, s, OCH_3), 6.60 (1H, ddd, ^4J = 1.0 Hz, ^4J = 2.5 Hz, ^3J = 8.1 Hz, H-4’), 7.10 (1H, ddd, ^4J = 1.1 Hz, ^4J = 1.6 Hz, ^3J = 8.1 Hz, H-6’), 7.18 (1H, t, ^3J = 8.0 Hz, H-5’), 7.31 (1H, dd, ^4J = 2.1 Hz, ^4J = 2.1 Hz, H-2’).

^{13}C NMR (75 MHz, DMSO-d_6): δ 28.7 (CH_2), 31.0 (CH_2), 104.6 (C-2’), 108.3 (C-4’), 111.1 (C-6’), 129.3 (C-5’), 140.4 (C-1’), 159.4 (C-3’), 170.0 (NHCO), 173.7 (COOH).

N-Arylsuccinimides 4c, 4d, 4f, and 4i; General Procedure

A mixture of *N*-arylsuccinamic acid (**6**) (20 mmol) and potassium acetate (5.89 g, 60 mmol) in acetic anhydride (66.70 mL) was heated under reflux for 1 h. After cooling, the solvent was evaporated under vacuum. To the resultant reaction mixture was added aqueous sodium carbonate (0.8 M, 50 mL), stirred for 10 min at room temperature and extracted using dichloromethane (3 x 20 ml). The organic layer was collected and dried over magnesium sulphate overnight and evaporated under vacuum. The crude product was washed with diethyl ether and filtered. Analytical sample was recrystallised from a suitable solvent.

N-(2-chlorophenyl)succinimide (4c)

Light brown solid; yield: 3.52 g (84%); mp 110-112 °C (Et_2O).

^{1}H NMR (300 MHz, DMSO-d_6): δ 2.78 (4H, s, CH_2CH_2), 7.24-7.27 (2H, m, H-2’ and H-6’), 7.38-7.43 (1H, m, H-4’), 7.45-7.51 (2H, m, H-3’ and H-5’).

^{13}C NMR (75 MHz, DMSO-d_6): δ 28.5 (CH_2CH_2), 128.0 (C-1’), 129.7 (C-5’), 130.5 (C-3’ and C-6’), 130.7 (C-4’), 131.3 (C-2’), 176.0 (2 x C=O).

N-(3-chlorophenyl)succinimide (4d)

White solid; yield: 3.73 g (89%); mp 117-119 °C (H_2O), lit.[2] 107-109 °C.

^{1}H NMR (300 MHz, DMSO-d_6): δ 2.78 (4H, s, CH_2CH_2), 7.27 (1H, ddd, ^4J = 1.8 Hz, ^4J = 1.8 Hz, ^3J = 7.2 Hz, H-6'), 7.39-7.40 (1H, m, H-2'), 7.46-7.50 (2H, m, H-4' and H-5').

^{13}C NMR (75 MHz, DMSO-d_6): δ 28.4 (CH_2CH_2), 125.8 (C-6'), 126.9 (C-2'), 128.0 (C-4'), 130.3 (C-5'), 132.8 (C-3'), 134.0 (C-1'), 176.5 (2 x C=O).

N-(3-methylphenyl)succinimide (4f)

Brown solid; yield: 3.46 g (92%); mp 112-114 °C (H_2O), lit.[4] 103-105 °C.

^{1}H NMR (300 MHz, DMSO-d_6): δ 2.33 (3H, s, CH_3), 2.77 (4H, s, CH_2CH_2), 7.03-7.06 (2H, m, H-2' and H-6'), 7.22 (1H, d, ^3J = 7.6 Hz, H-4'), 7.36 (1H, t, ^3J = 7.7 Hz, H-5').

^{13}C NMR (75 MHz, DMSO-d_6): δ 20.7 (CH_3), 28.4 (CH_2CH_2), 124.1 (C-6'), 127.4 (C-2'), 128.5 (C-5'), 128.7 (C-4'), 132.6 (C-1'), 138.1 (C-3'), 176.8 (2 x C=O).

N-(3-methoxyphenyl)succinimide (4i)

Light brown solid; yield: 2.91 g (71%); mp 80-82 °C (Et_2O).

^{1}H NMR (300 MHz, DMSO-d_6): δ 2.77 (4H, s, CH_2CH_2), 3.76 (3H, s, OCH_3), 6.81-6.84 (2H, m, H-2' and H-6'), 7.00 (1H, ddd, ^4J = 1.0 Hz, ^4J = 2.5 Hz, ^3J = 8.4 Hz,' H-4'), 7.39 (1H, t, ^3J = 8.3 Hz, H-5')

^{13}C NMR (75 MHz, DMSO-d_6): δ 28.4 (CH_2CH_2), 55.2 (OCH_3), 112.9 (C-2'), 113.6 (C-4'), 119.3 (C-6'), 129.5 (C-5'), 133.8 (C-1'), 159.3 (C-3'), 176.7 (2 x C=O).

References

1. Chernyshev, V. M.; Chernysheva, A. V.; Starikova, Z. A. *Heterocycles* **2010,** *81*, 2291.
2. Garad, D. N.; Tanpure, S. D.; Mhaske, S. B. *Beilstein J. Org. Chem.* **2015,** *11,* 1008.
3. Le, Z.-G.; Chen, Z.-C.; Hu, Y.; Zheng, Q.-G. *Synthesis* **2004,** *7,* 995.
4. Kar, A.; Argade, N. P. *Synthesis* **2002,** *2*, 221.
5. Habash, M.; Taha, M. O. *Bioorg. Med. Chem.* **2011,** *19*, 4746.

^{1}H and ^{13}C NMR spectra of succinamic acids 6

N-(2-chlorophenyl)succinamic acid (6a)

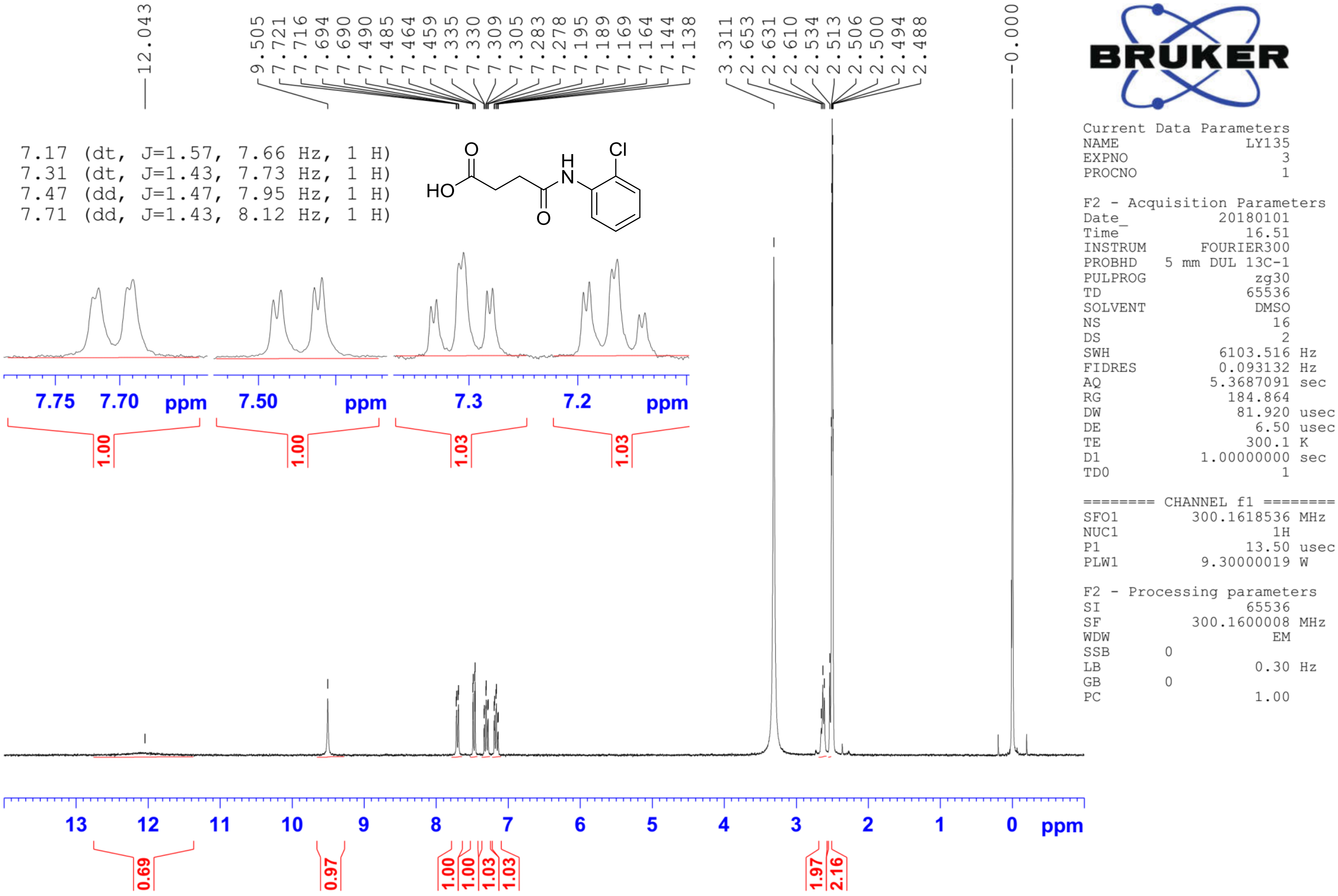

N-(2-chlorophenyl)succinamic acid (6a)

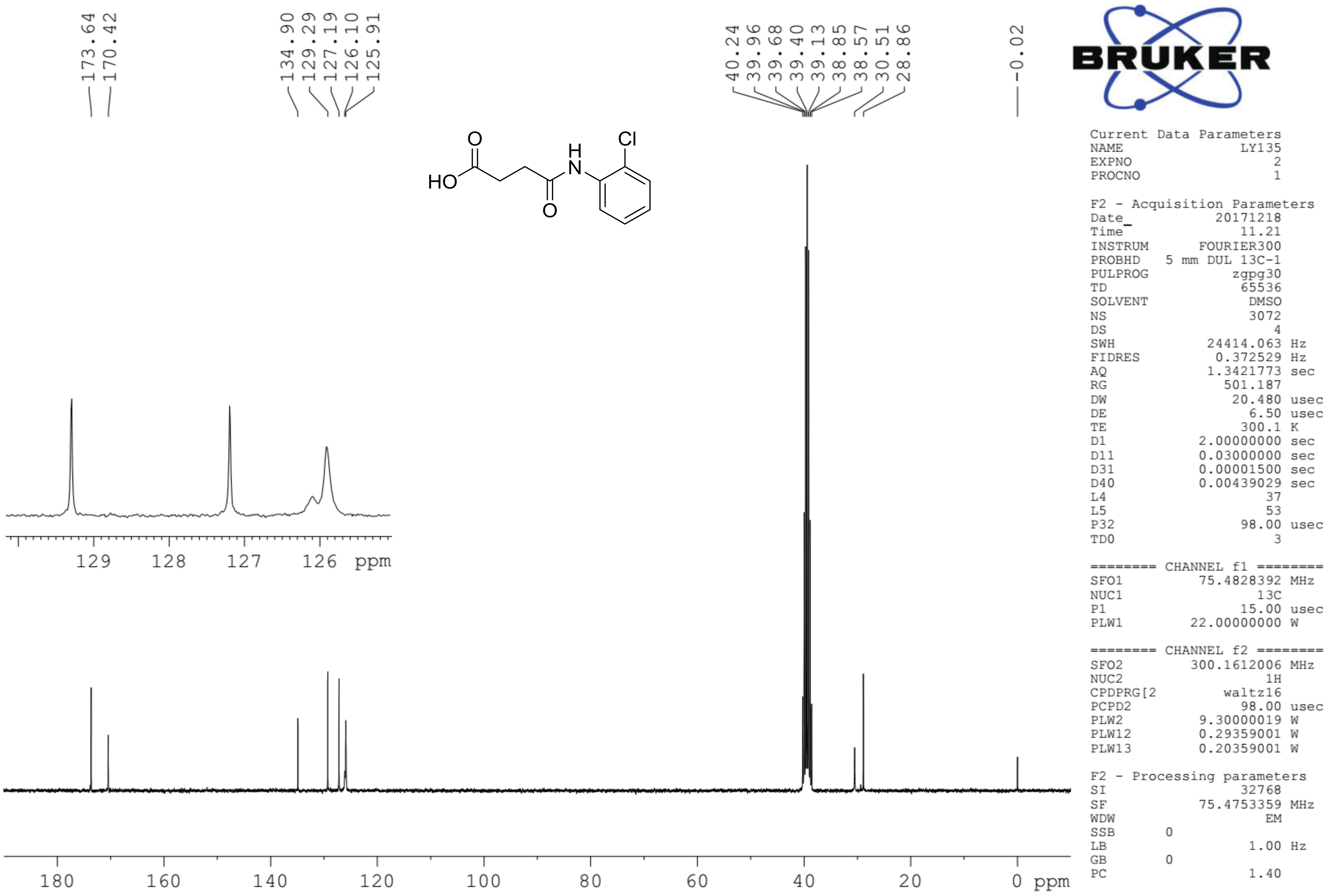

N-(3-chlorophenyl)succinamic acid (6b)

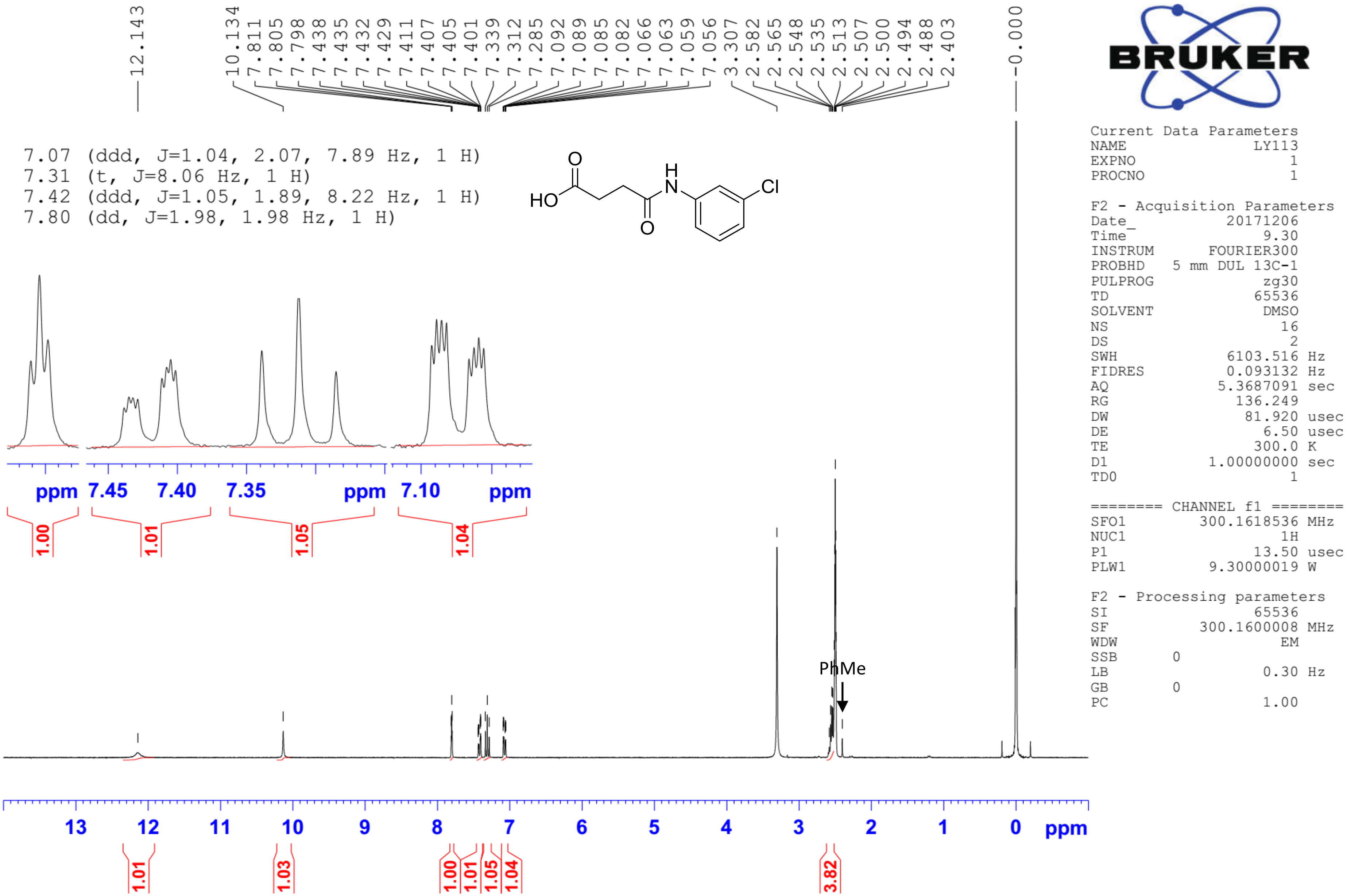

N-(3-chlorophenyl)succinamic acid (6b)

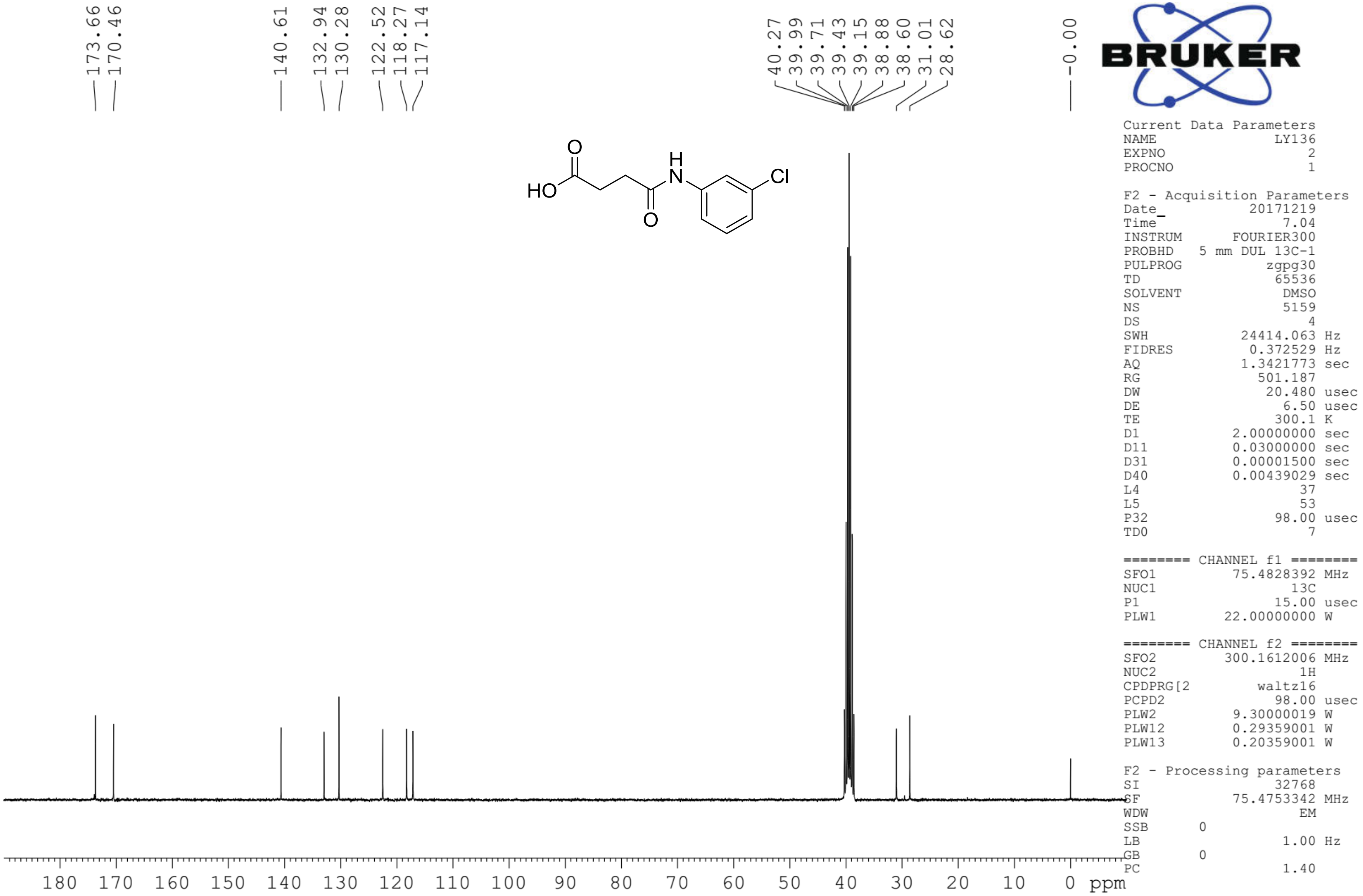

N-(3-methylphenyl)succinamic acid (6c)

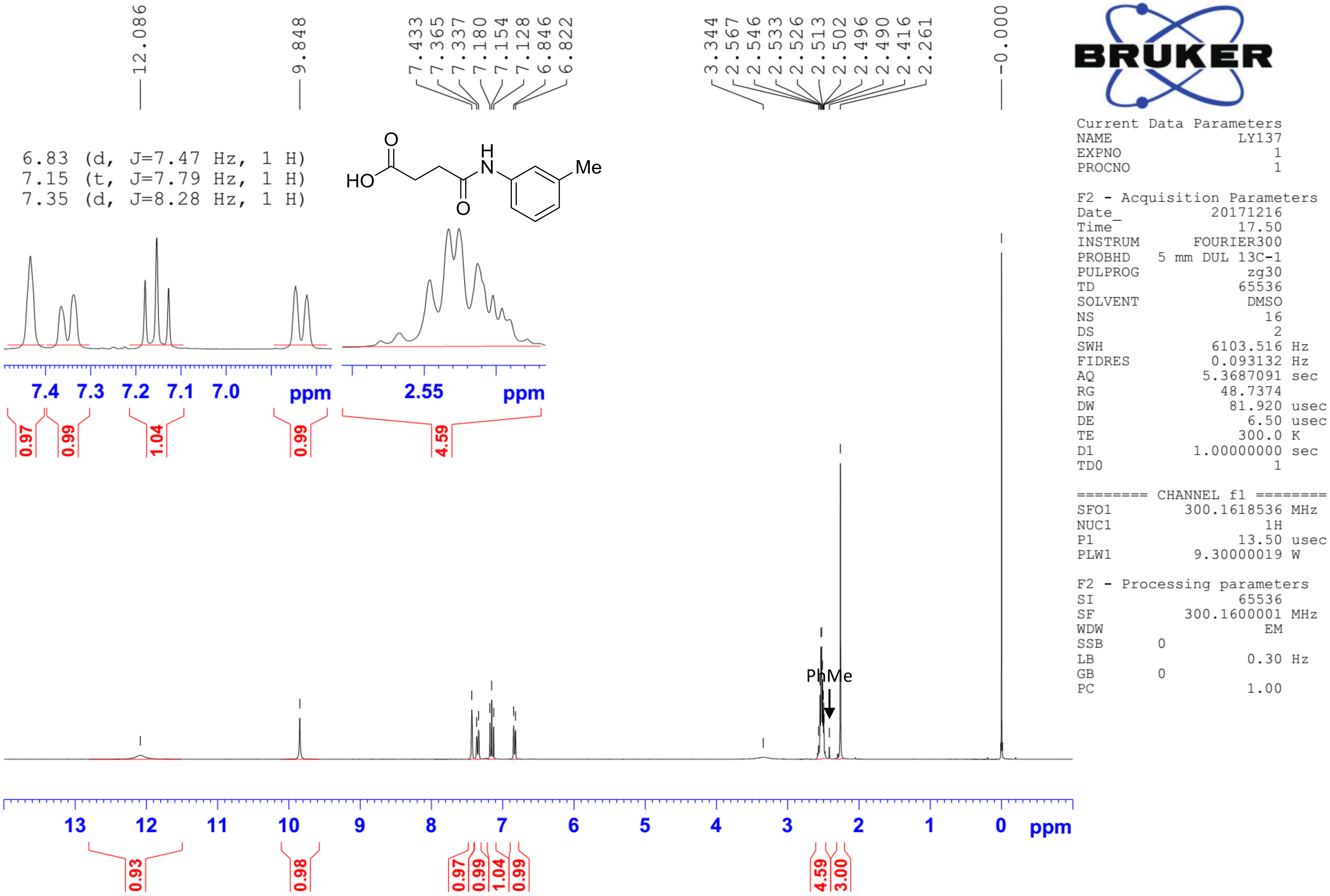

***N*-(3-methylphenyl)succinamic acid (6c)**

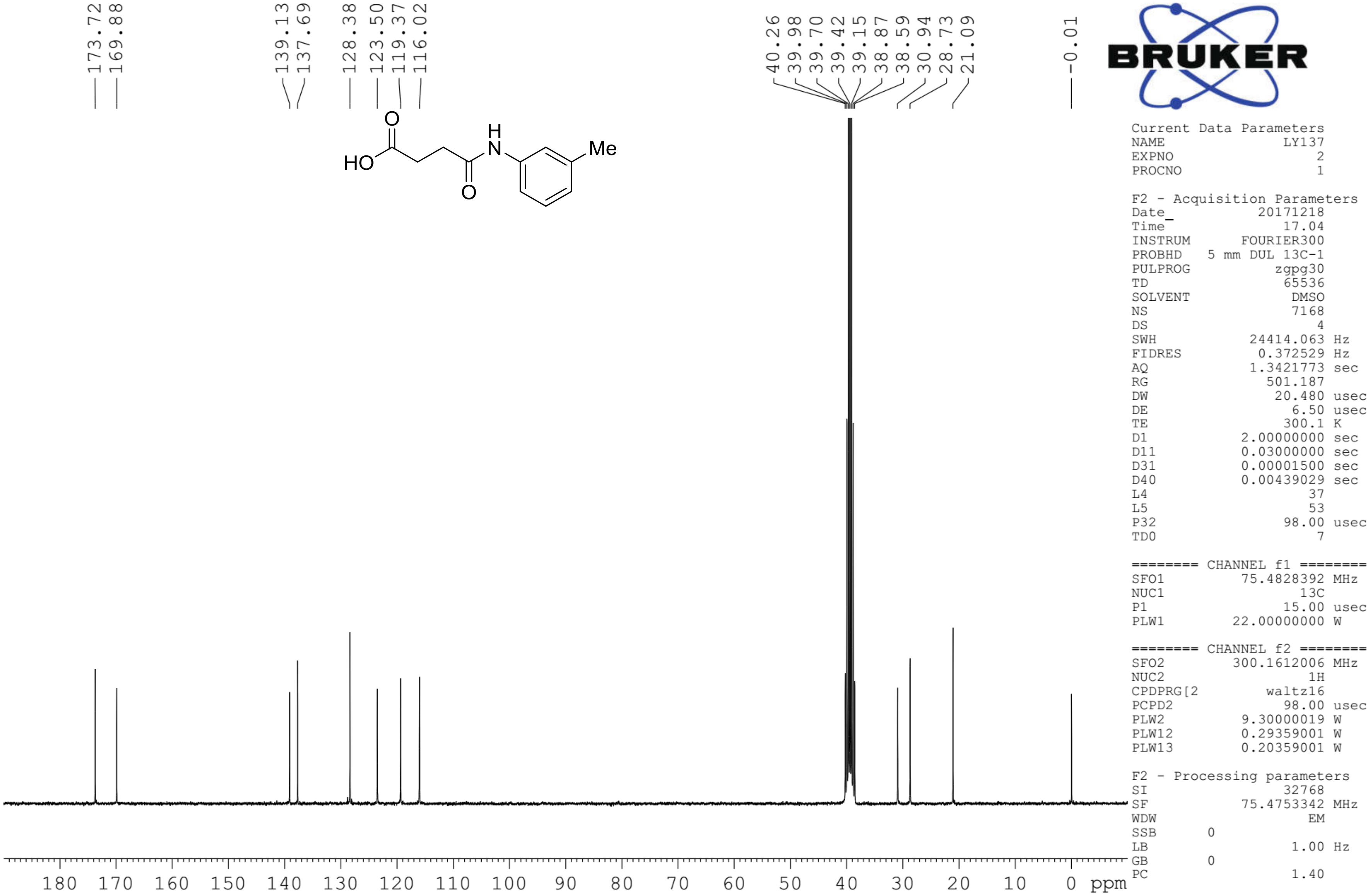

N-(3-methoxyphenyl)succinamic acid (6d)

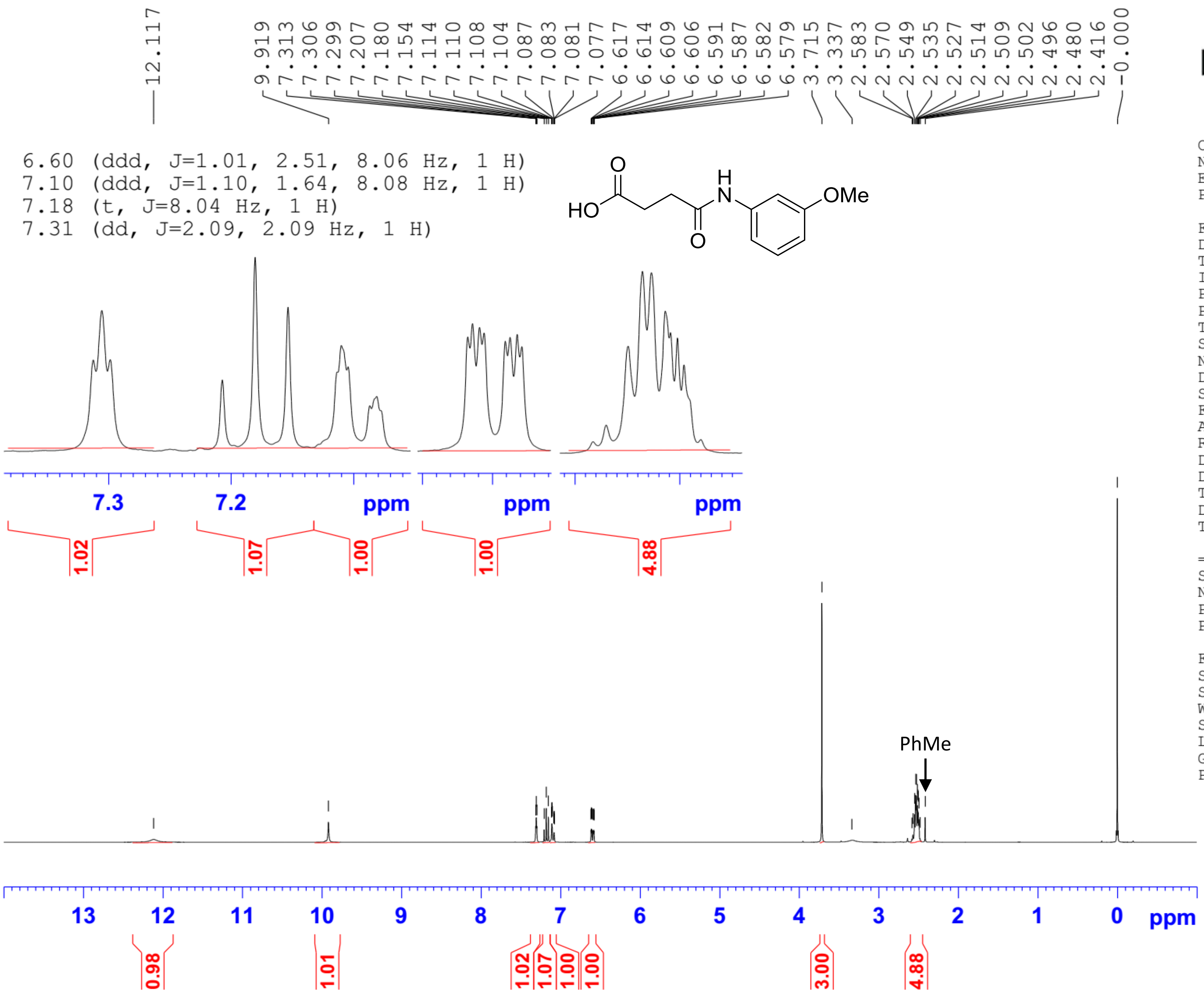

Current Data Parameters	
NAME	LY138
EXPNO	1
PROCNO	1
F2 - Acquisition Parameters	
Date_	20171216
Time	17.54
INSTRUM	FOURIER300
PROBHD	5 mm DUL 13C-1
PULPROG	zg30
TD	65536
SOLVENT	DMSO
NS	16
DS	2
SWH	6103.516 Hz
FIDRES	0.093132 Hz
AQ	5.3687091 sec
RG	76.8407
DW	81.920 usec
DE	6.50 usec
TE	300.0 K
D1	1.00000000 sec
TD0	1
======== CHANNEL f1 ========	
SFO1	300.1618536 MHz
NUC1	1H
P1	13.50 usec
PLW1	9.30000019 W
F2 - Processing parameters	
SI	65536
SF	300.1600001 MHz
WDW	EM
SSB	0
LB	0.30 Hz
GB	0
PC	1.00

N-(3-methoxyphenyl)succinamic acid (6d)

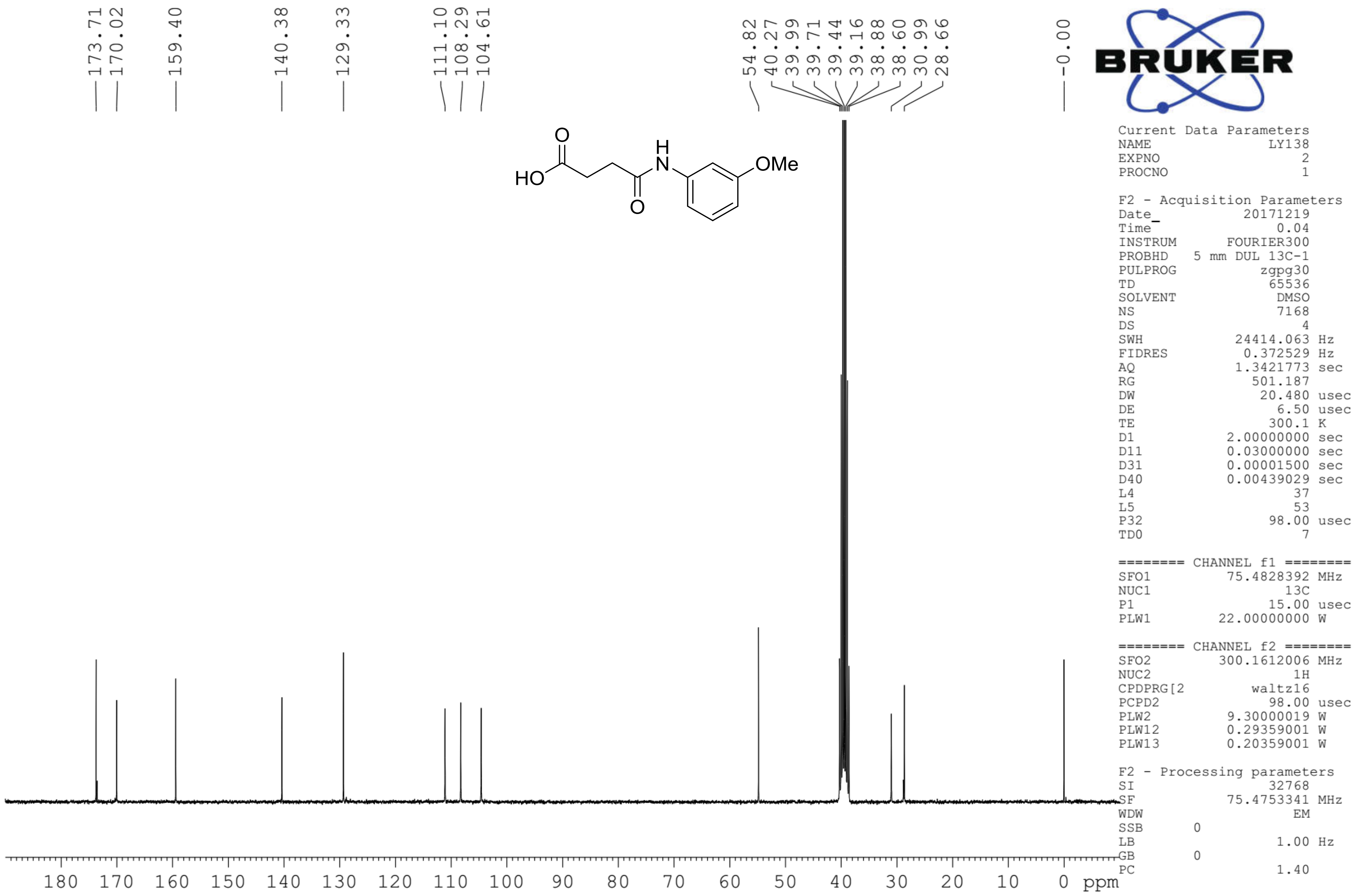

^{1}H and ^{13}C NMR spectra of succimimides 2 and 4

N-guanidinosuccinimide (2)

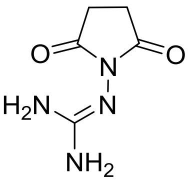

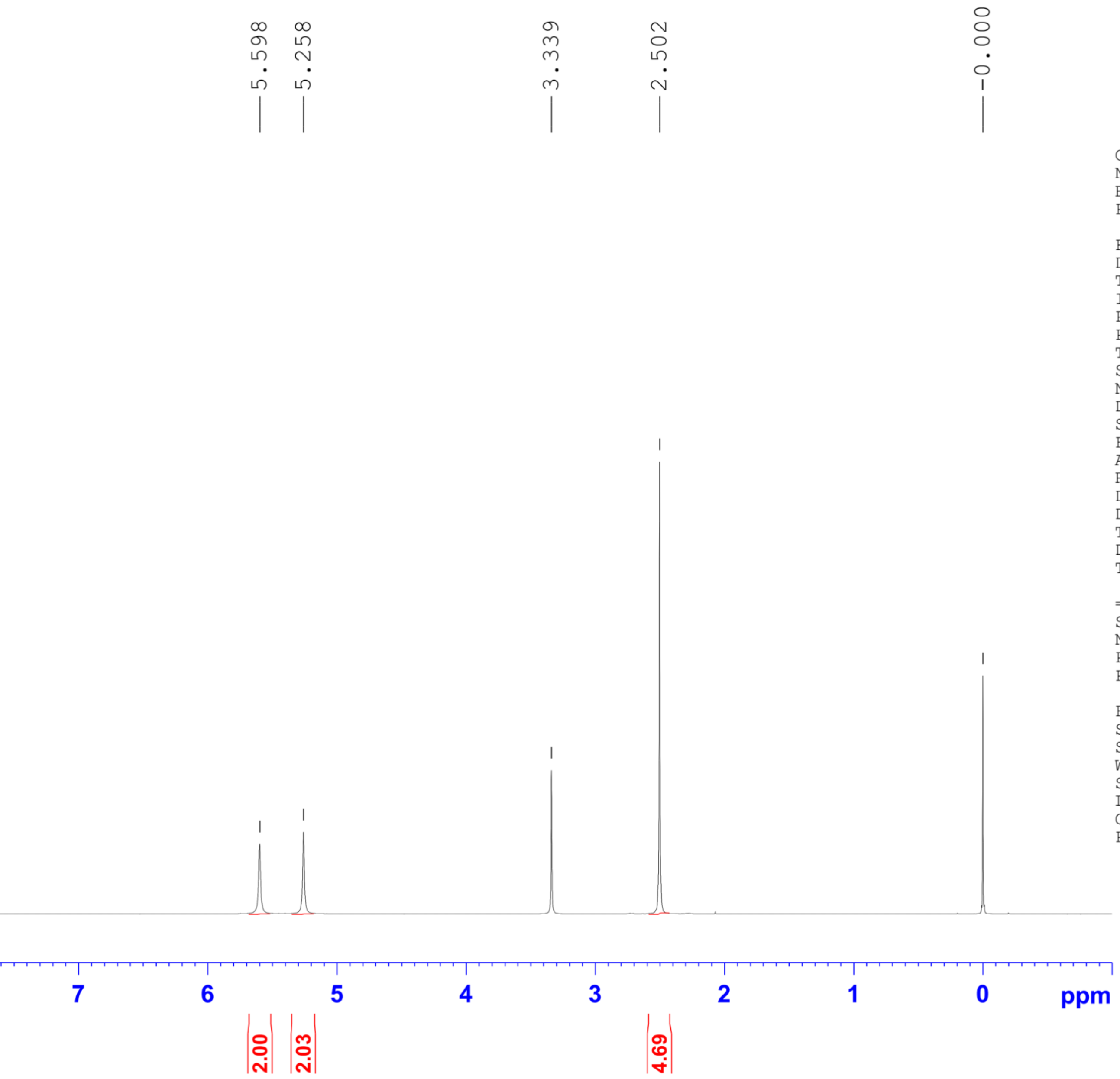

Current Data Parameters
NAME LY143
EXPNO 1
PROCNO 1

F2 - Acquisition Parameters
Date_ 20171226
Time 15.45
INSTRUM FOURIER300
PROBHD 5 mm DUL 13C-1
PULPROG zg30
TD 65536
SOLVENT DMSO
NS 16
DS 2
SWH 6103.516 Hz
FIDRES 0.093132 Hz
AQ 5.3687091 sec
RG 48.7008
DW 81.920 usec
DE 6.50 usec
TE 300.0 K
D1 1.00000000 sec
TD0 1

======== CHANNEL f1 ========
SFO1 300.1618536 MHz
NUC1 1H
P1 13.50 usec
PLW1 9.30000019 W

F2 - Processing parameters
SI 65536
SF 300.1600001 MHz
WDW EM
SSB 0
LB 0.30 Hz
GB 0
PC 1.00

***N*-guanidinosuccinimide (2)**

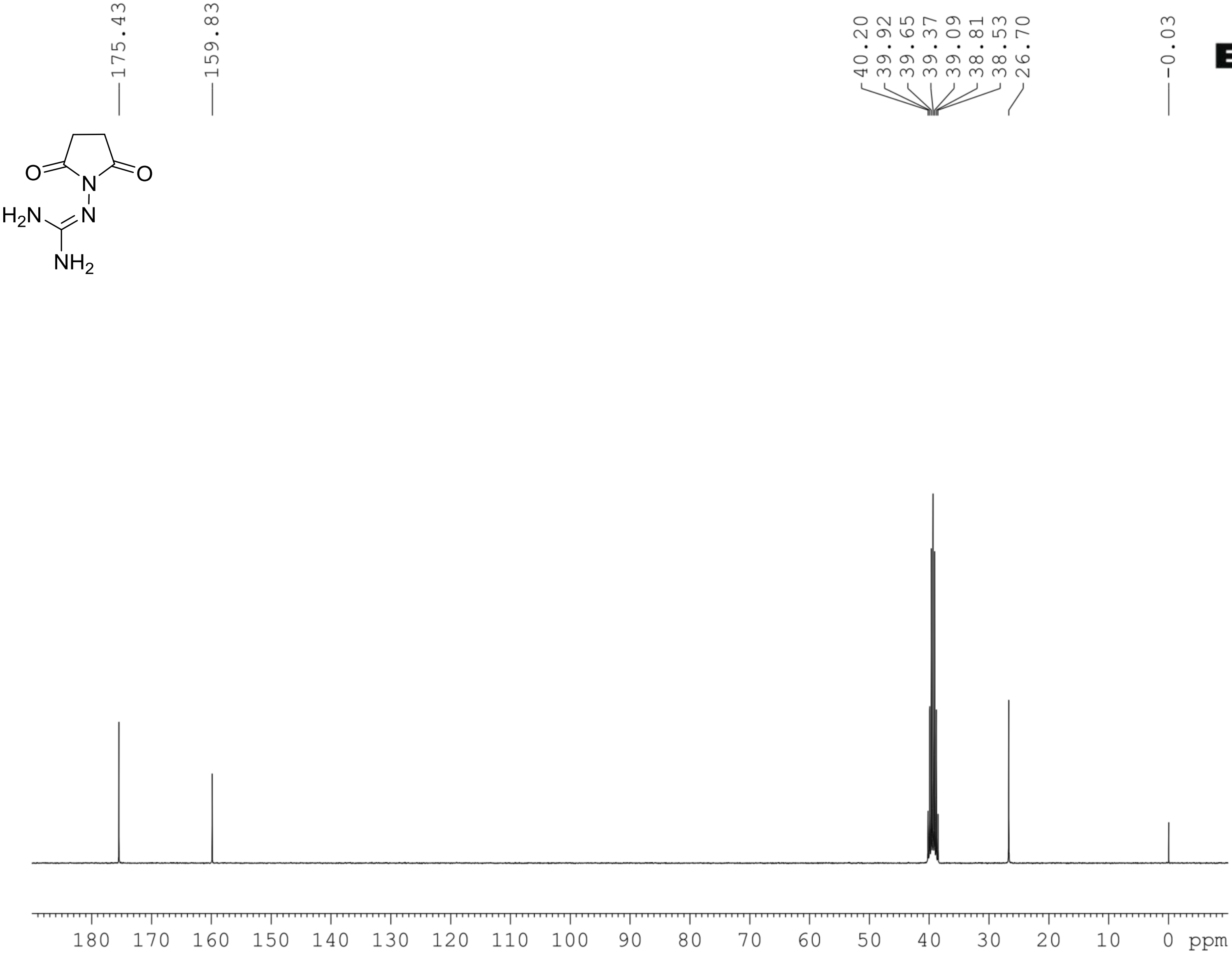

BRUKER

Current Data Parameters

NAME	LY143
EXPNO	2
PROCNO	1

F2 - Acquisition Parameters

Date_	20171230
Time	21.23
INSTRUM	FOURIER300
PROBHD	5 mm DUL 13C-1
PULPROG	zgpg30
TD	65536
SOLVENT	DMSO
NS	8192
DS	4
SWH	24414.063 Hz
FIDRES	0.372529 Hz
AQ	1.3421773 sec
RG	501.187
DW	20.480 usec
DE	6.50 usec
TE	300.1 K
D1	2.00000000 sec
D11	0.03000000 sec
D31	0.00001500 sec
D40	0.00439029 sec
L4	37
L5	53
P32	98.00 usec
TD0	8

======== CHANNEL f1 ========

SFO1	75.4828392 MHz
NUC1	13C
P1	15.00 usec
PLW1	22.00000000 W

======== CHANNEL f2 ========

SFO2	300.1612006 MHz
NUC2	1H
CPDPRG[2	waltz16
PCPD2	98.00 usec
PLW2	9.30000019 W
PLW12	0.29359001 W
PLW13	0.20359001 W

F2 - Processing parameters

SI	32768
SF	75.4753376 MHz
WDW	EM
SSB	0
LB	1.00 Hz
GB	0
PC	1.40

N-phenylsuccinimide (4a)

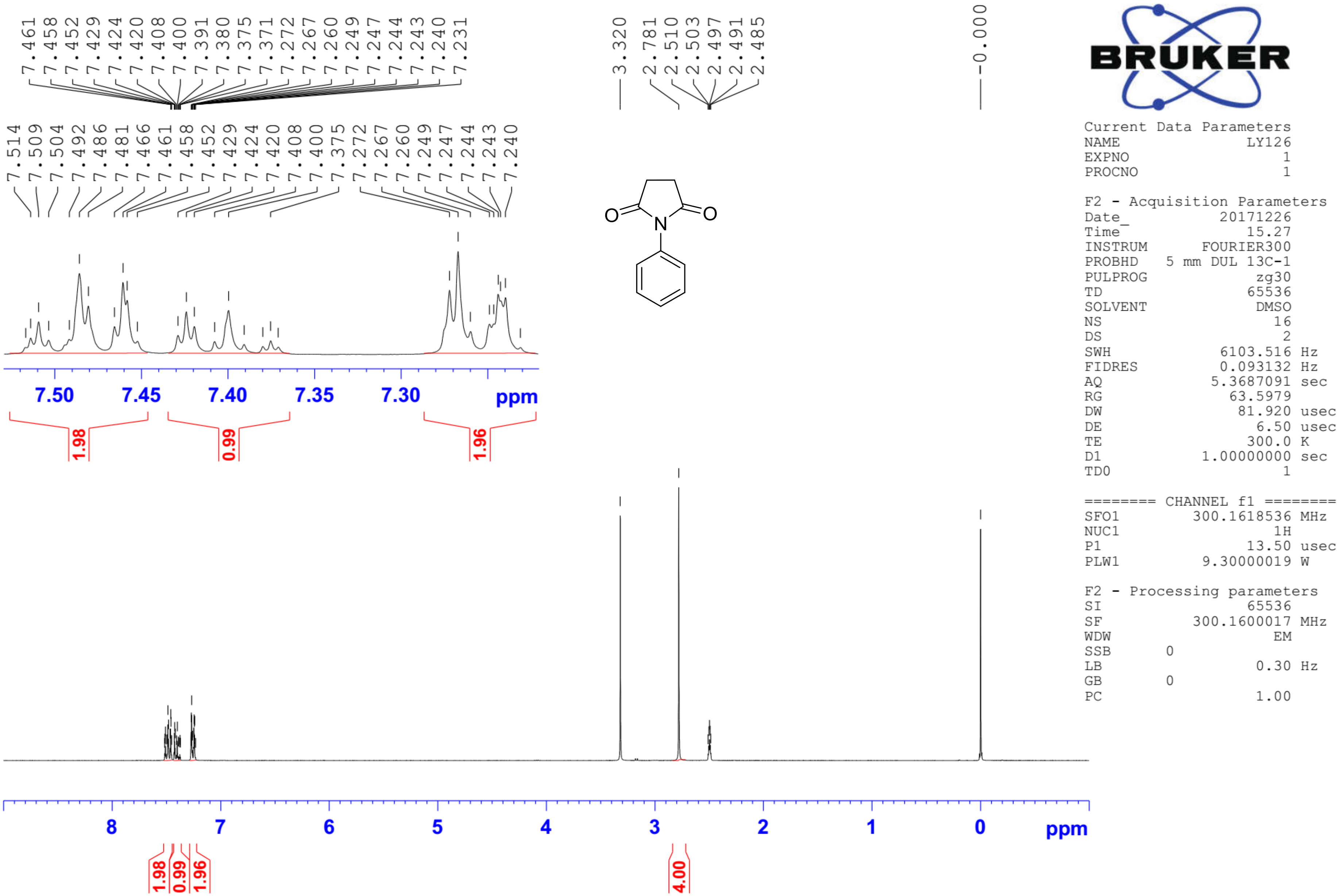

***N*-phenylsuccinimide (4a)**

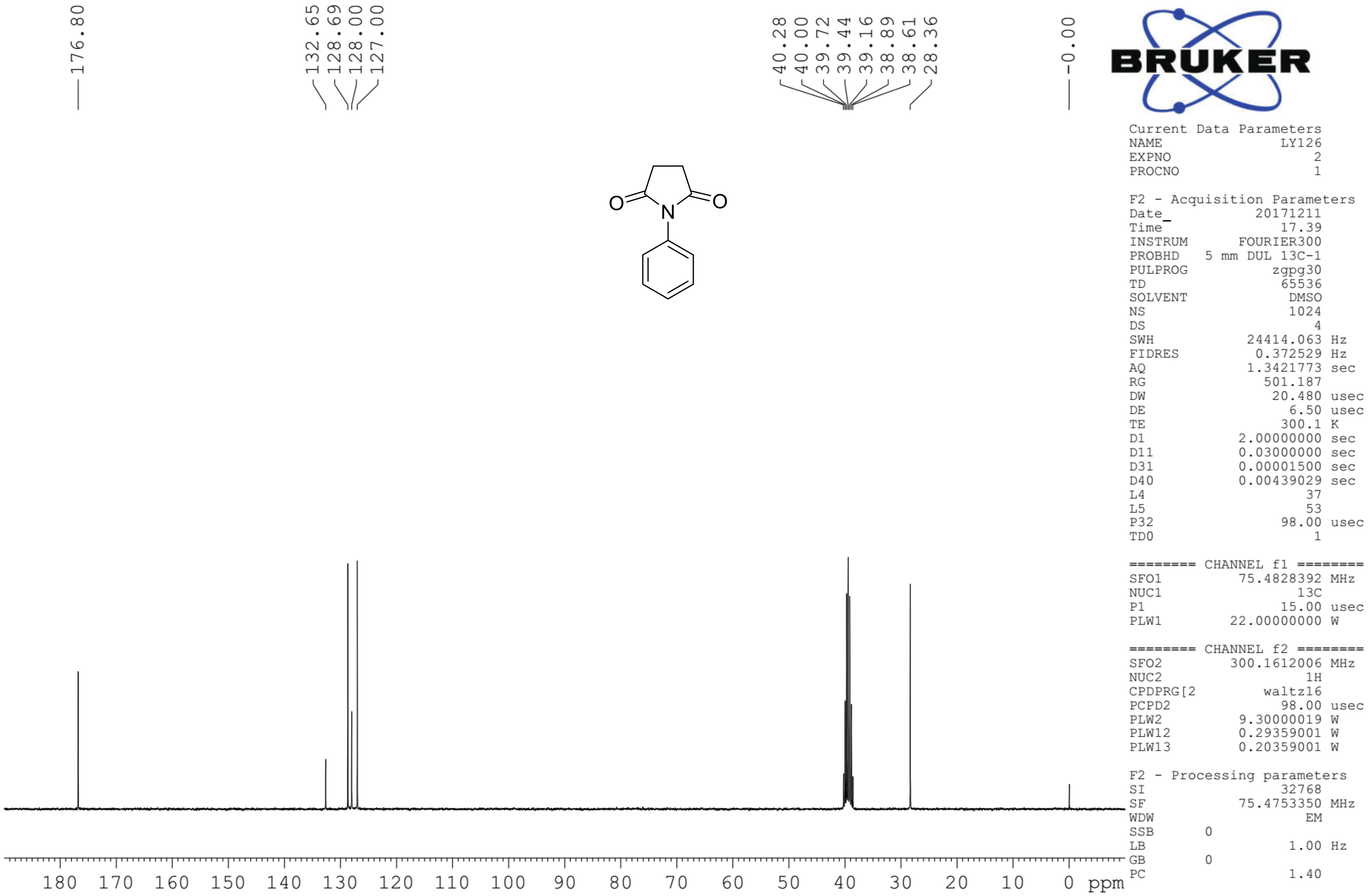

N-(4-fluorophenyl)succinimide (4b)

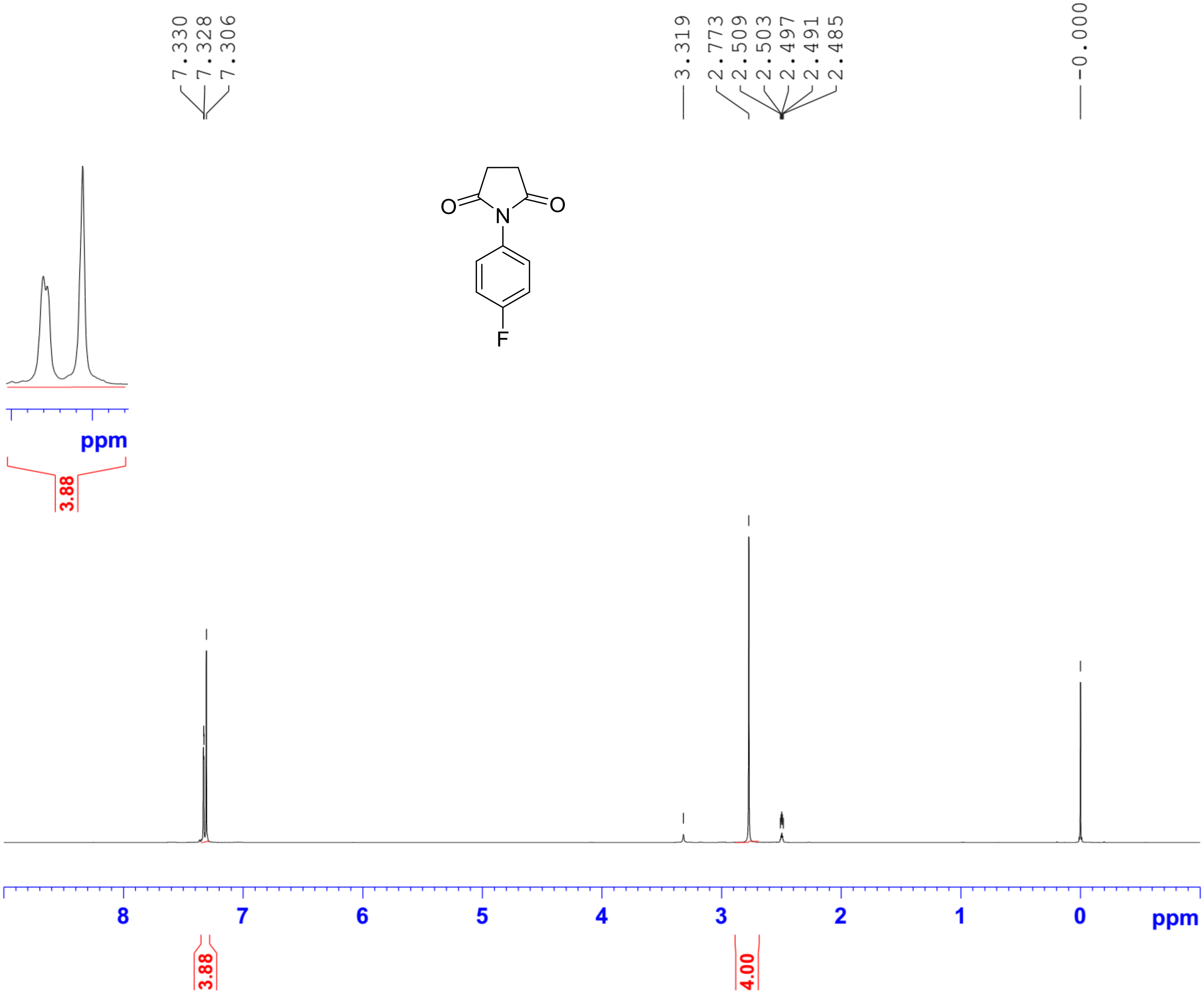


```
Current Data Parameters
NAME              LY129
EXPNO                 1
PROCNO                1

F2 - Acquisition Parameters
Date_          20171212
Time              17.53
INSTRUM      FOURIER300
PROBHD   5 mm DUL 13C-1
PULPROG            zg30
TD                65536
SOLVENT            DMSO
NS                   16
DS                    2
SWH            6103.516 Hz
FIDRES         0.093132 Hz
AQ            5.3687091 sec
RG               31.623
DW               81.920 usec
DE                 6.50 usec
TE                300.0 K
D1           1.00000000 sec
TD0                   1

======== CHANNEL f1 ========
SFO1        300.1618536 MHz
NUC1                 1H
P1                13.50 usec
PLW1         9.30000019 W

F2 - Processing parameters
SI                65536
SF          300.1600016 MHz
WDW                  EM
SSB      0
LB                 0.30 Hz
GB       0
PC                 1.00
```

N-(4-fluorophenyl)succinimide (4b)

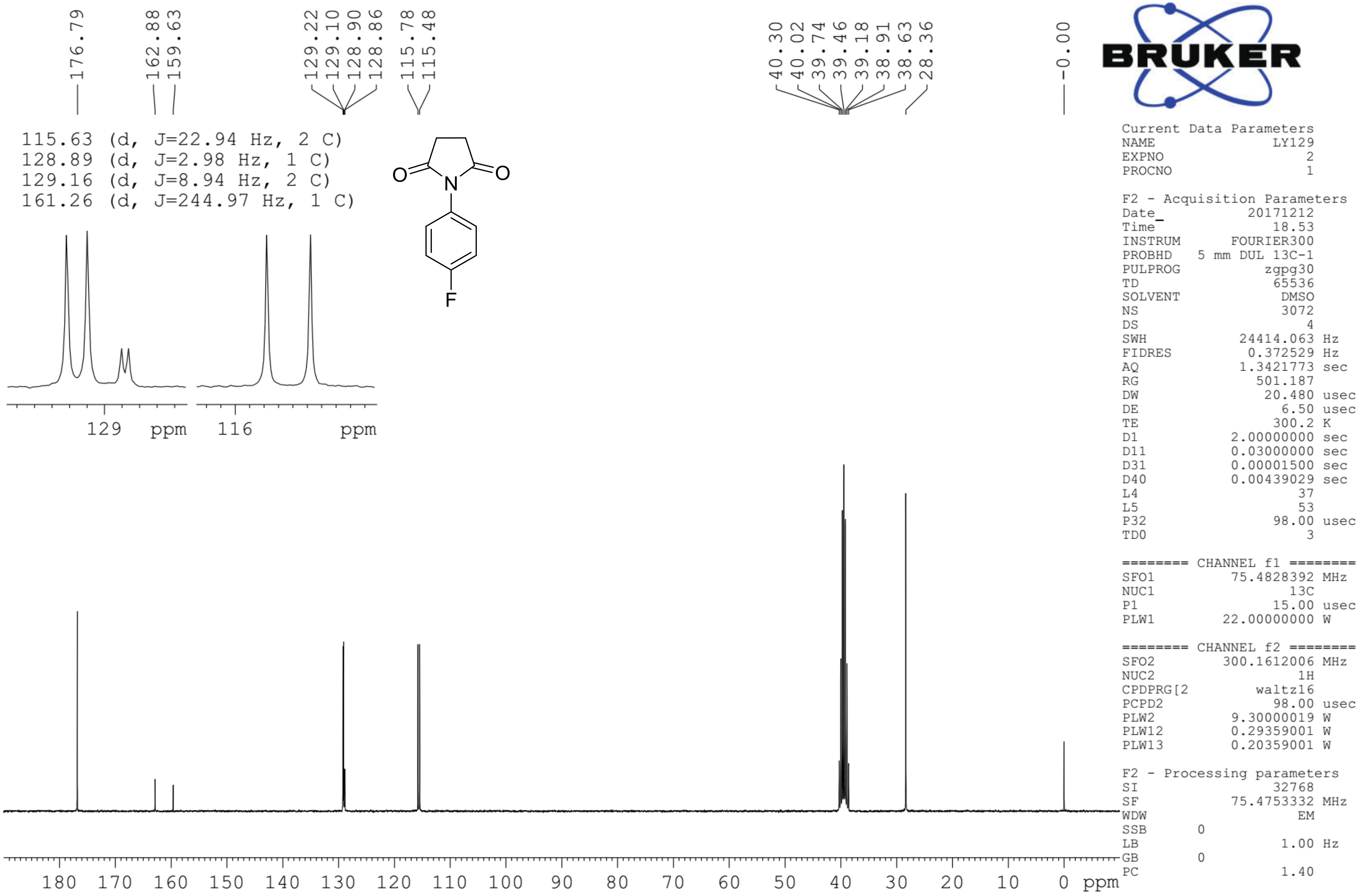

N-(2-chlorophenyl)succinimide (4c)

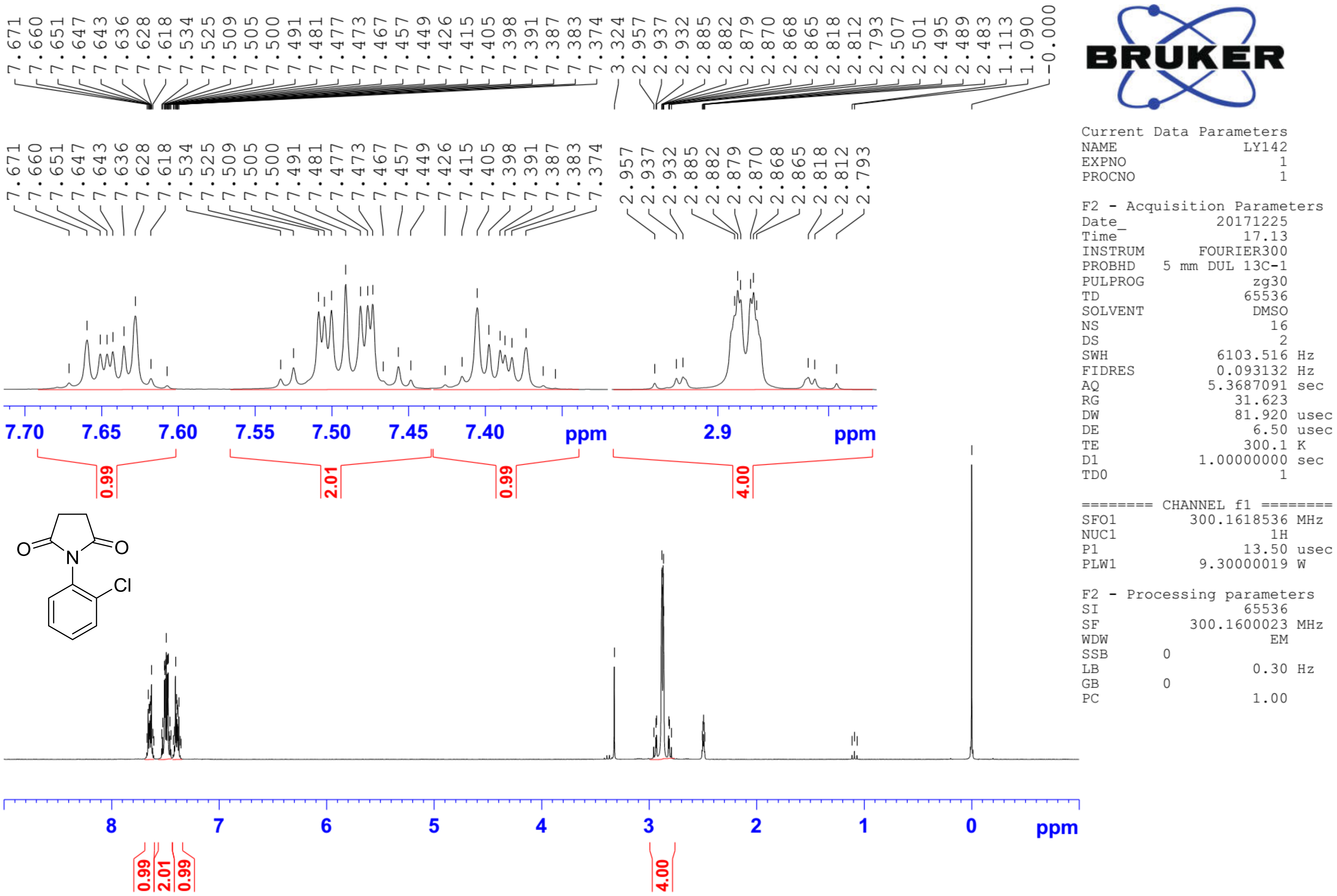

N-(2-chlorophenyl)succinimide (4c)

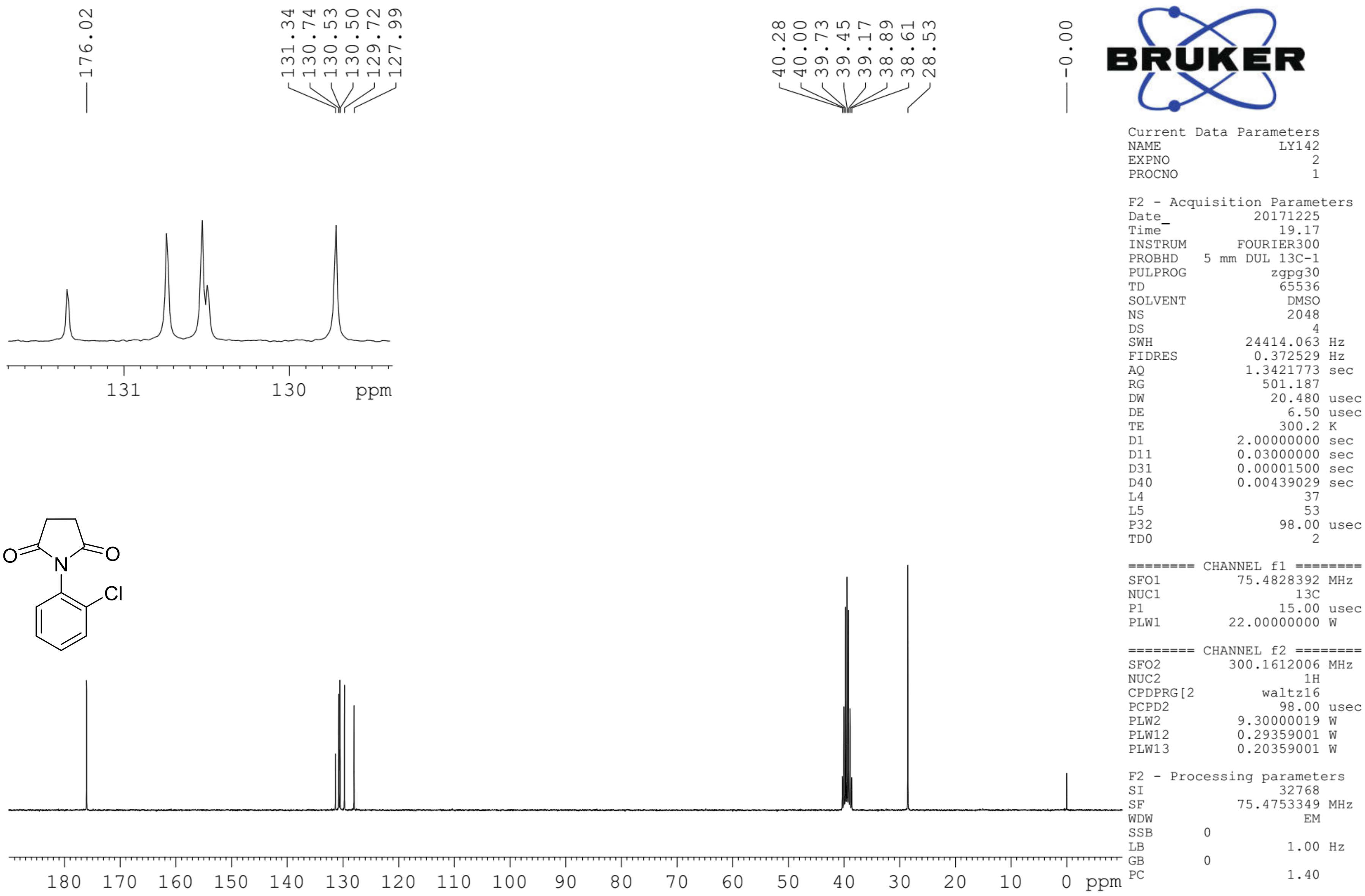

N-(3-chlorophenyl)succinimide (4d)

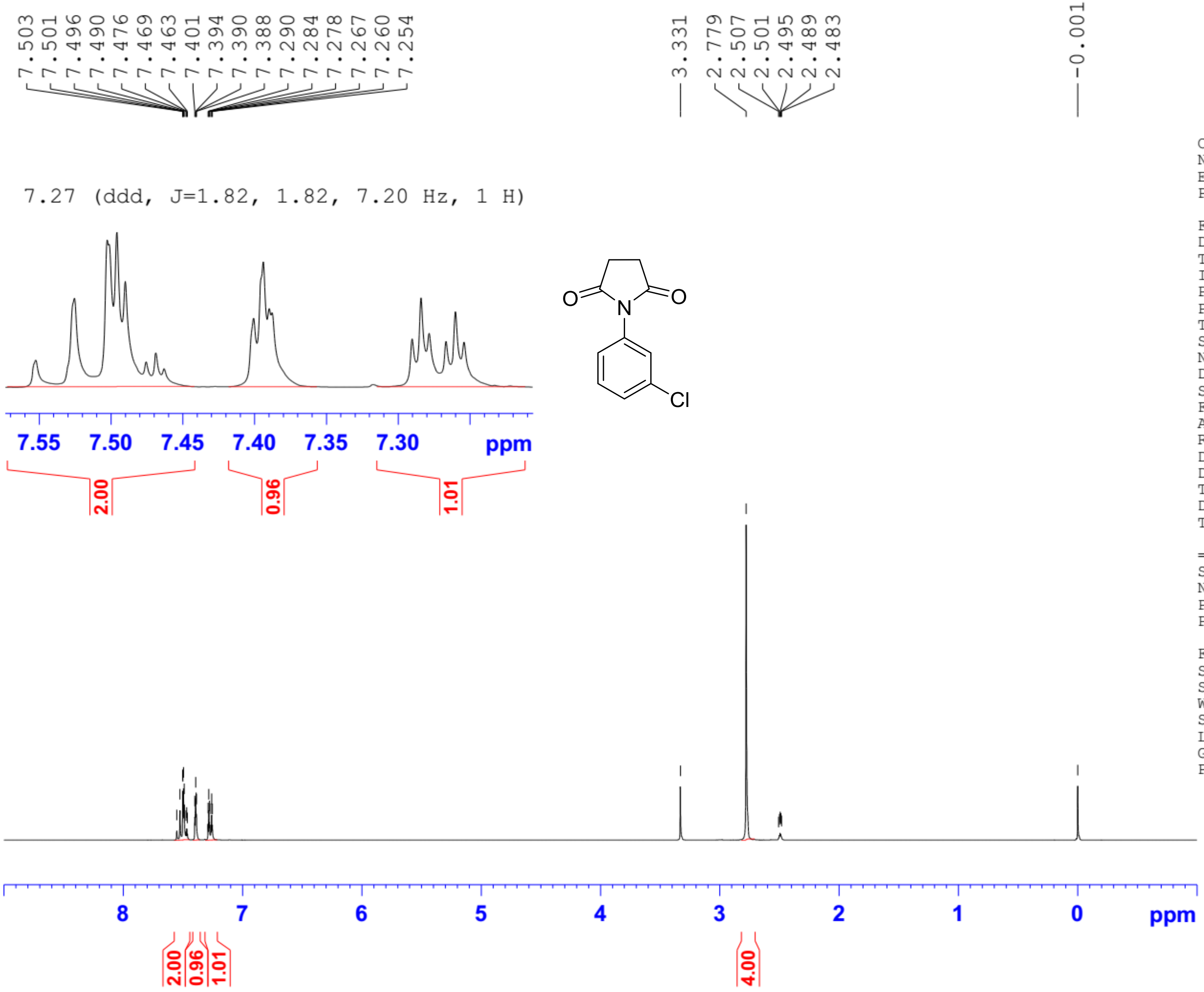

Current Data Parameters
NAME LY133
EXPNO 3
PROCNO 1

F2 - Acquisition Parameters
Date_ 20171216
Time 17.18
INSTRUM FOURIER300
PROBHD 5 mm DUL 13C-1
PULPROG zg30
TD 65536
SOLVENT DMSO
NS 16
DS 2
SWH 6103.516 Hz
FIDRES 0.093132 Hz
AQ 5.3687091 sec
RG 31.623
DW 81.920 usec
DE 6.50 usec
TE 299.9 K
D1 1.00000000 sec
TD0 1

======== CHANNEL f1 ========
SFO1 300.1618536 MHz
NUC1 1H
P1 13.50 usec
PLW1 9.30000019 W

F2 - Processing parameters
SI 65536
SF 300.1600026 MHz
WDW EM
SSB 0
LB 0.30 Hz
GB 0
PC 1.00

N-(3-chlorophenyl)succinimide (4d)

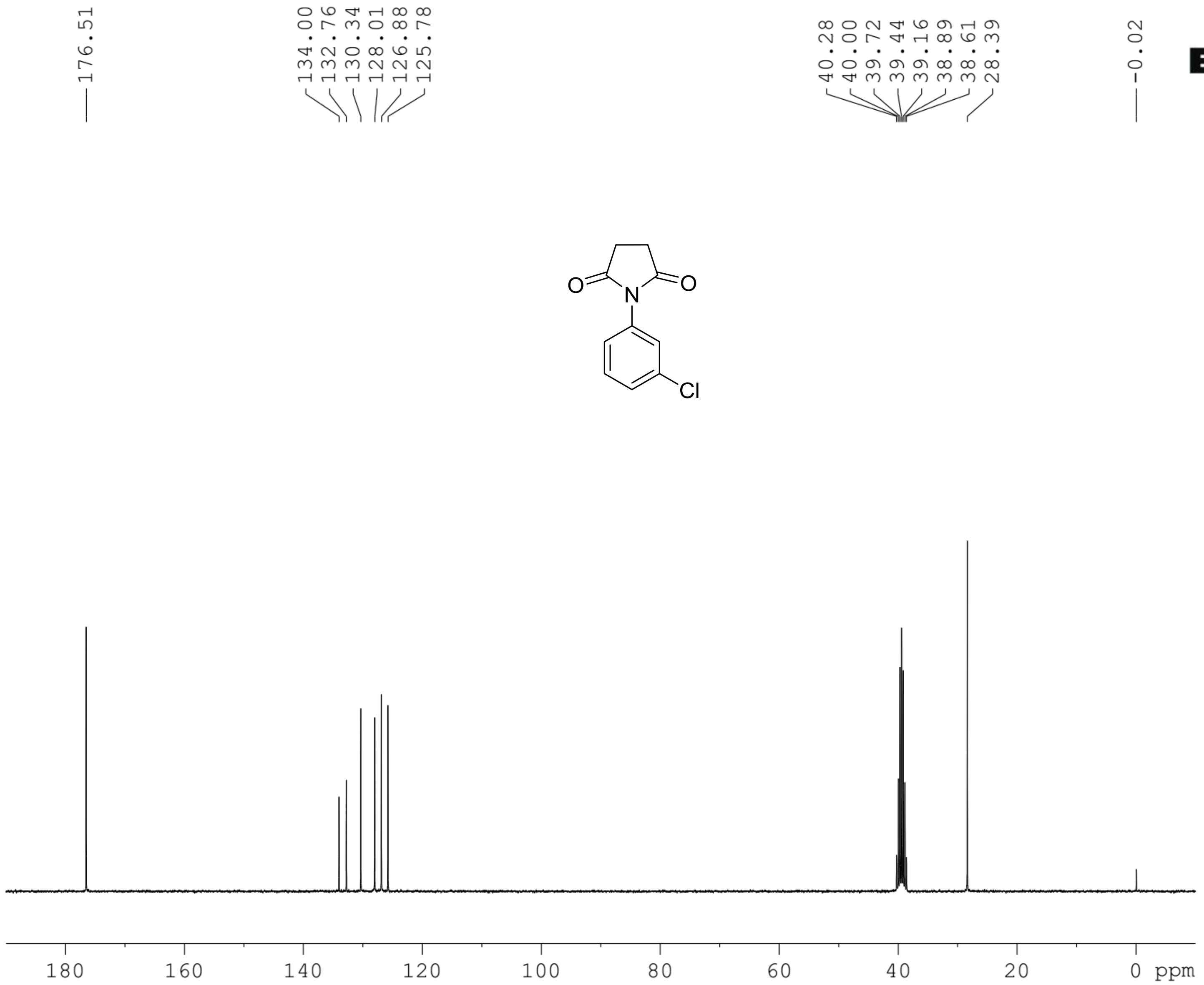

BRUKER

```
Current Data Parameters
NAME                LY133
EXPNO                   2
PROCNO                  1

F2 - Acquisition Parameters
Date_            20171215
Time                 7.39
INSTRUM        FOURIER300
PROBHD   5 mm DUL 13C-1
PULPROG            zgpg30
TD                  65536
SOLVENT              DMSO
NS                   1024
DS                      4
SWH             24414.063 Hz
FIDRES           0.372529 Hz
AQ              1.3421773 sec
RG                501.187
DW                 20.480 usec
DE                   6.50 usec
TE                  300.1 K
D1             2.00000000 sec
D11            0.03000000 sec
D31            0.00001500 sec
D40            0.00439029 sec
L4                     37
L5                     53
P32                 98.00 usec
TD0                     1

======== CHANNEL f1 ========
SFO1           75.4828392 MHz
NUC1                  13C
P1                  15.00 usec
PLW1          22.00000000 W

======== CHANNEL f2 ========
SFO2          300.1612006 MHz
NUC2                   1H
CPDPRG[2          waltz16
PCPD2               98.00 usec
PLW2           9.30000019 W
PLW12          0.29359001 W
PLW13          0.20359001 W

F2 - Processing parameters
SI                  32768
SF             75.4753350 MHz
WDW                    EM
SSB      0
LB                   1.00 Hz
GB       0
PC                   1.40
```

N-(4-chlorophenyl)succinimide (4e)

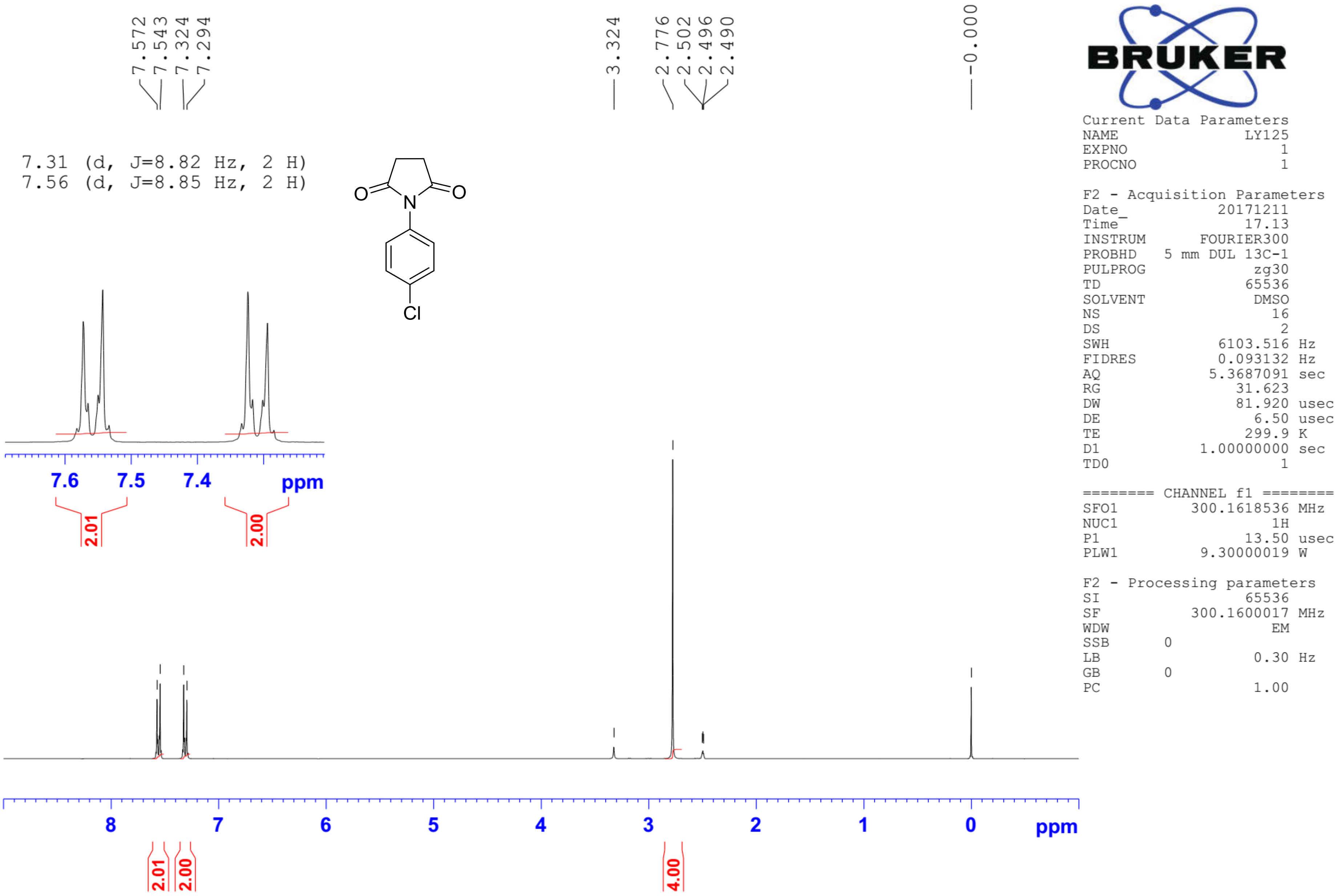

***N*-(4-chlorophenyl)succinimide (4e)**

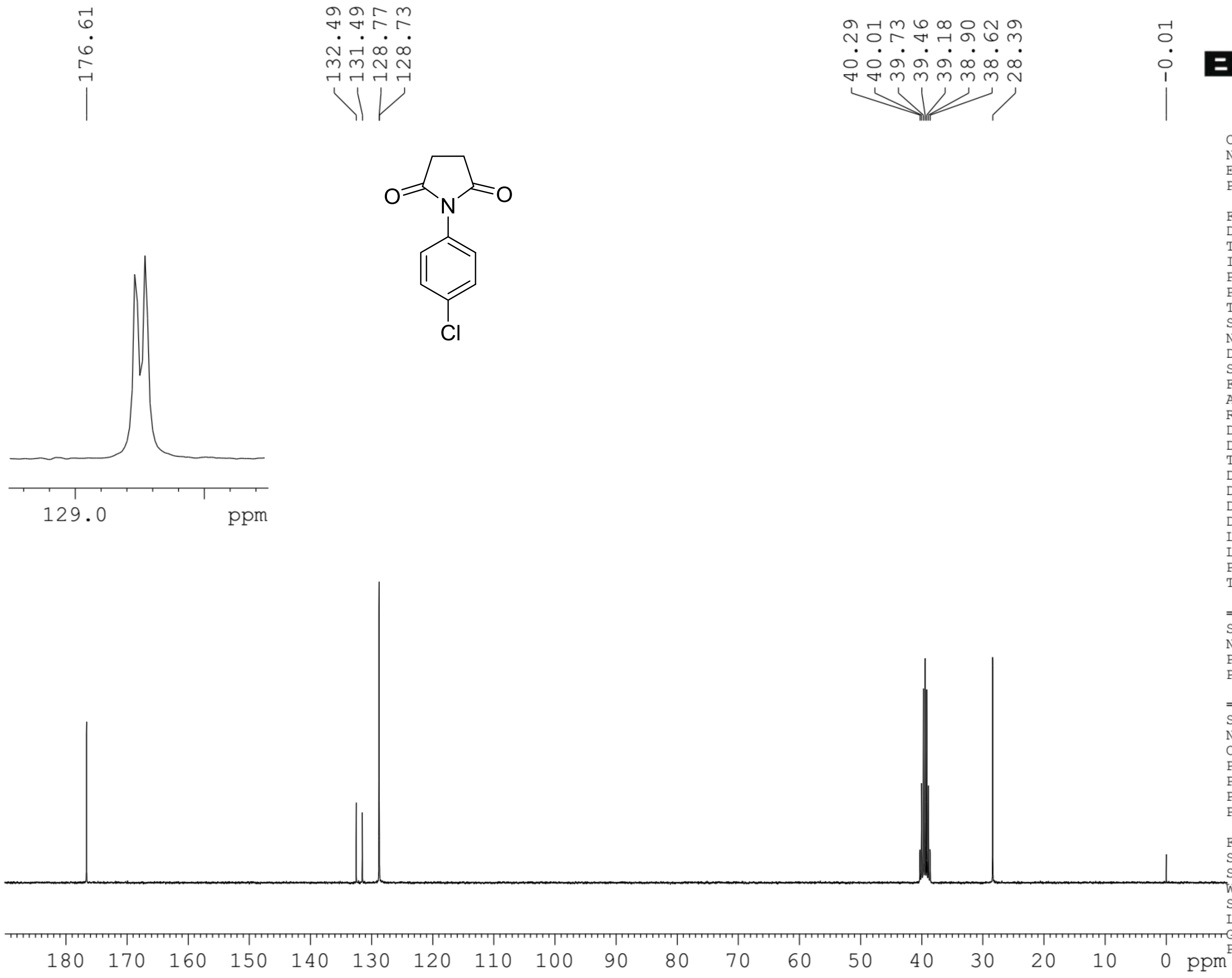


```
Current Data Parameters
NAME              LY125
EXPNO                 2
PROCNO                1

F2 - Acquisition Parameters
Date_          20171212
Time               9.17
INSTRUM      FOURIER300
PROBHD   5 mm DUL 13C-1
PULPROG         zgpg30
TD                65536
SOLVENT            DMSO
NS                 1024
DS                    4
SWH           24414.063 Hz
FIDRES         0.372529 Hz
AQ            1.3421773 sec
RG              501.187
DW               20.480 usec
DE                 6.50 usec
TE                300.1 K
D1           2.00000000 sec
D11          0.03000000 sec
D31          0.00001500 sec
D40          0.00439029 sec
L4                   37
L5                   53
P32               98.00 usec
TD0                   1

======== CHANNEL f1 ========
SFO1        75.4828392 MHz
NUC1               13C
P1               15.00 usec
PLW1       22.00000000 W

======== CHANNEL f2 ========
SFO2       300.1612006 MHz
NUC2                1H
CPDPRG[2       waltz16
PCPD2            98.00 usec
PLW2        9.30000019 W
PLW12       0.29359001 W
PLW13       0.20359001 W

F2 - Processing parameters
SI               32768
SF          75.4753342 MHz
WDW                 EM
SSB      0
LB                1.00 Hz
GB       0
PC                1.40
```

N-(3-methylphenyl)succinimide (4f)

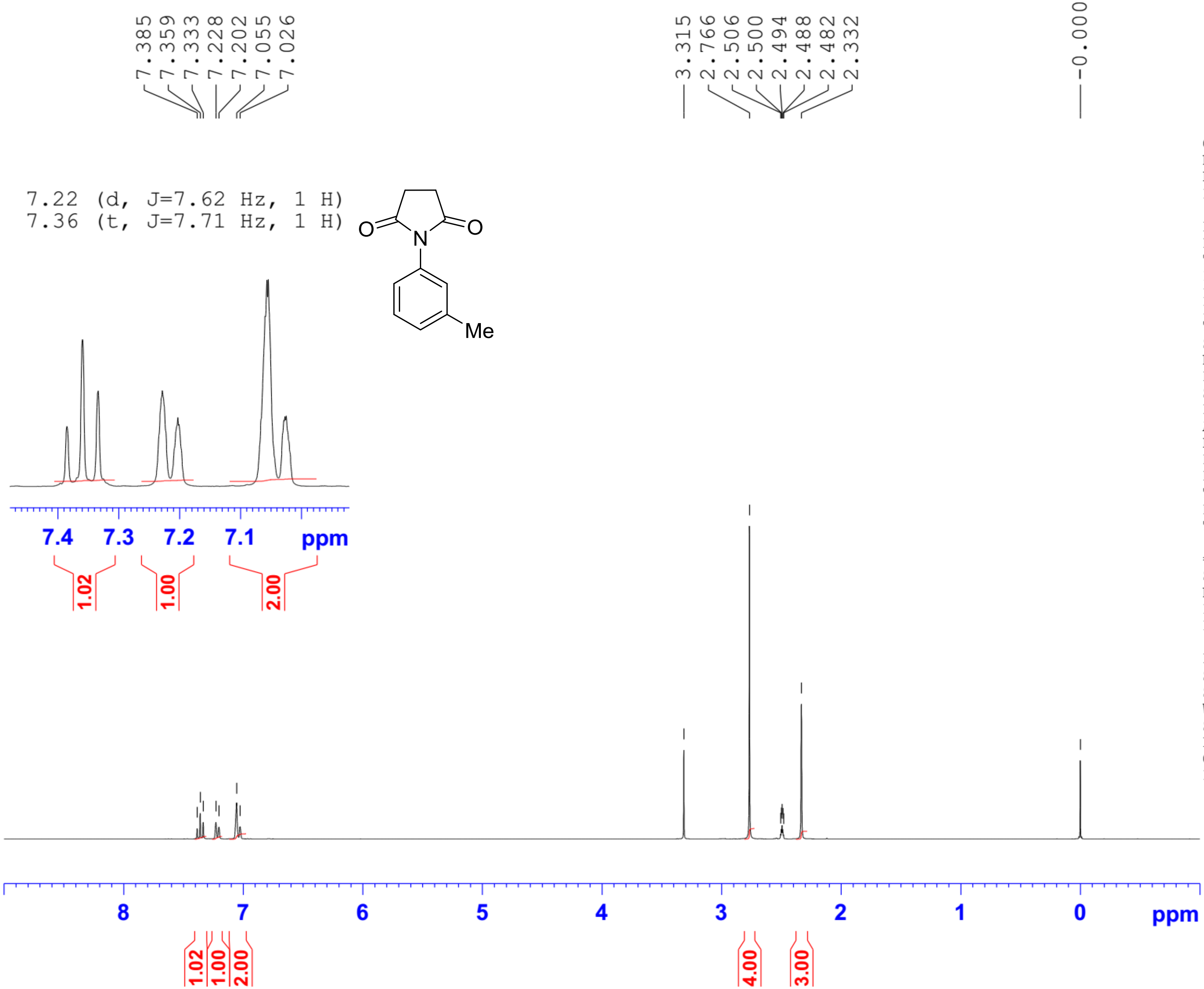


```
Current Data Parameters
NAME              LY139
EXPNO                 1
PROCNO                1

F2 - Acquisition Parameters
Date_          20171219
Time              13.20
INSTRUM      FOURIER300
PROBHD   5 mm DUL 13C-1
PULPROG            zg30
TD                65536
SOLVENT            DMSO
NS                   16
DS                    2
SWH            6103.516 Hz
FIDRES         0.093132 Hz
AQ            5.3687091 sec
RG               31.623
DW               81.920 usec
DE                 6.50 usec
TE                300.0 K
D1           1.00000000 sec
TD0                   1

======== CHANNEL f1 ========
SFO1        300.1618536 MHz
NUC1                 1H
P1                13.50 usec
PLW1         9.30000019 W

F2 - Processing parameters
SI                65536
SF          300.1600026 MHz
WDW                  EM
SSB       0
LB                 0.30 Hz
GB        0
PC                 1.00
```

***N*-(3-methylphenyl)succinimide (4f)**

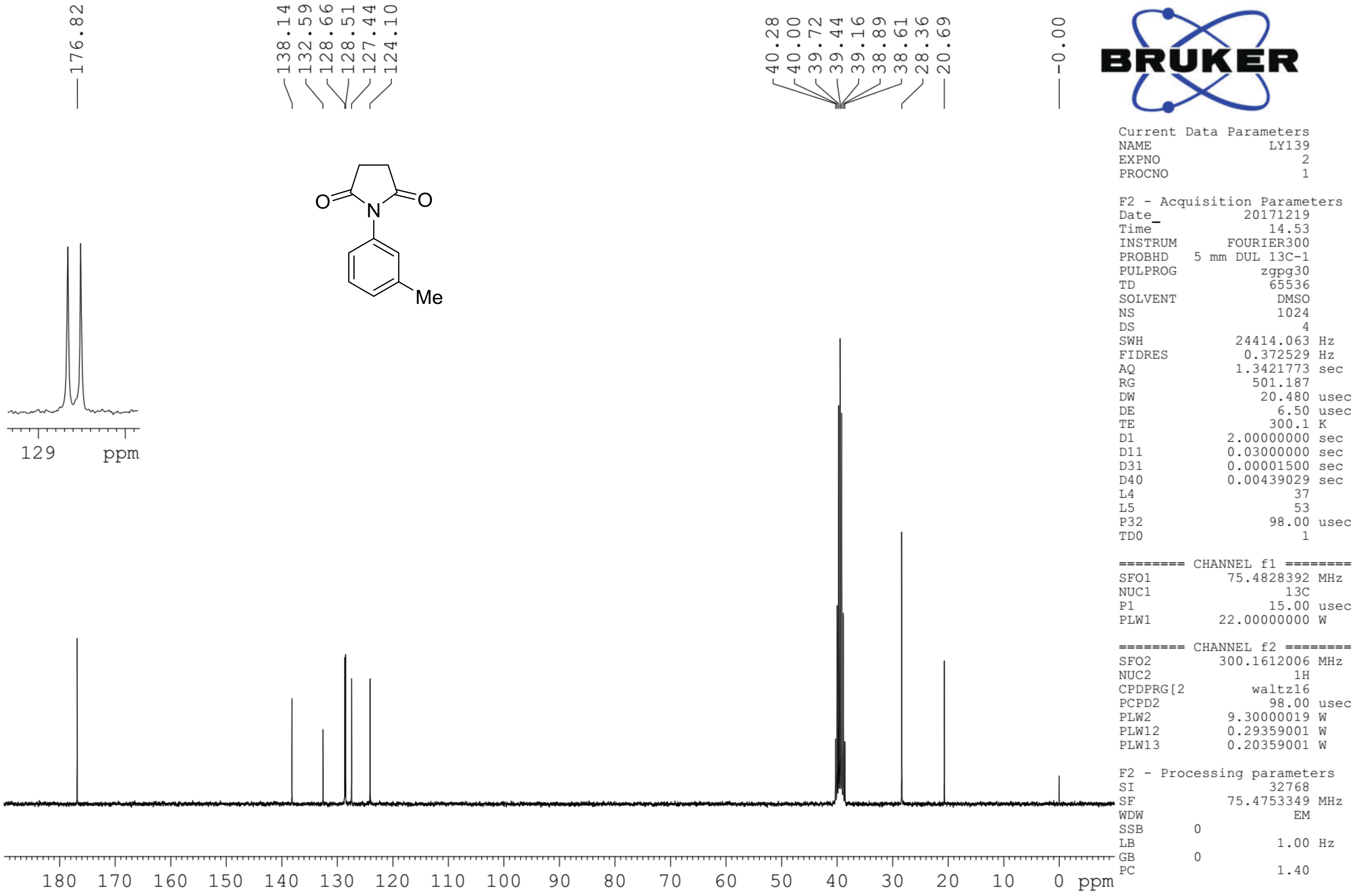

N-(4-methylphenyl)succinimide (4g)

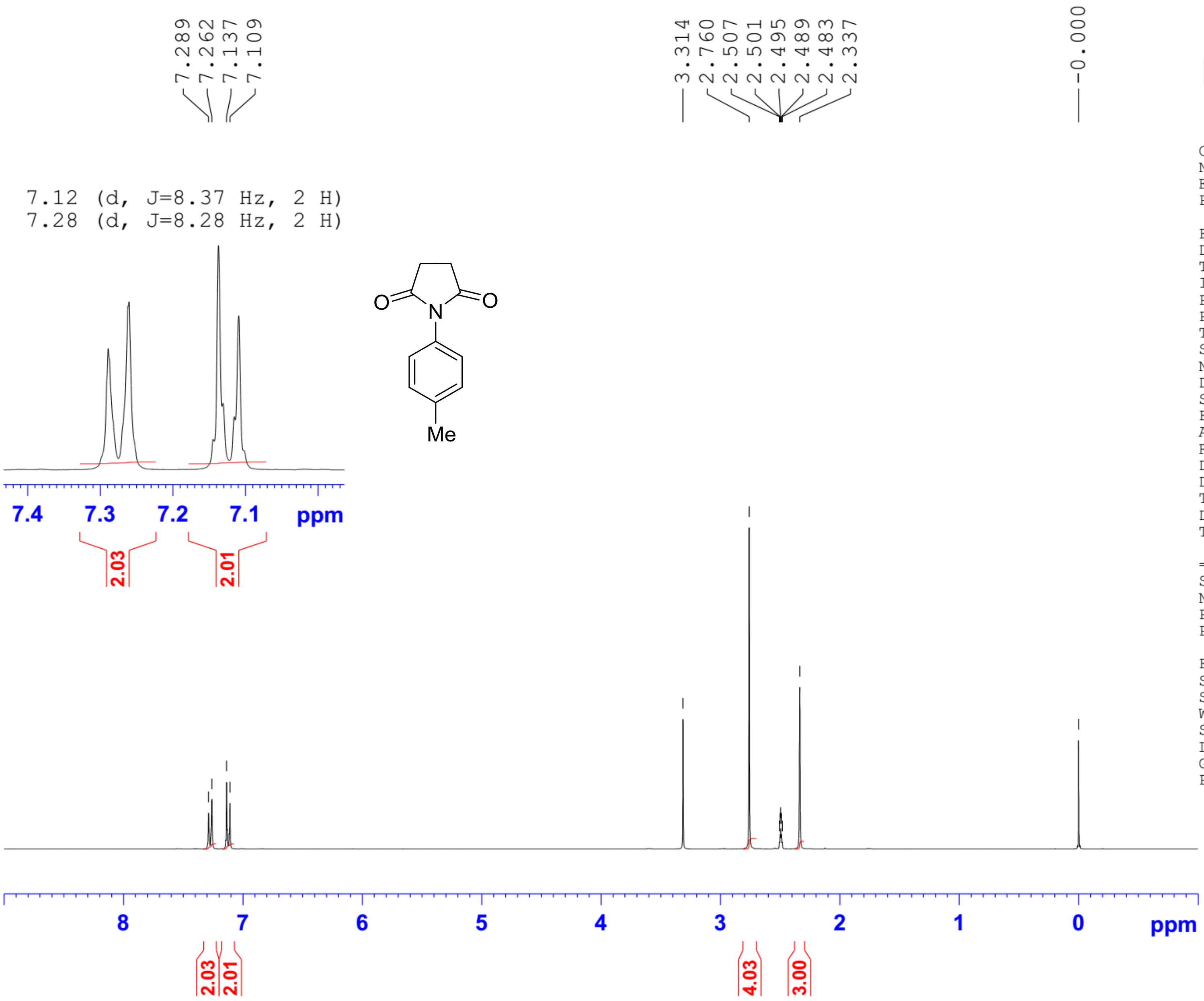

Current Data Parameters
NAME LY124
EXPNO 1
PROCNO 1

F2 - Acquisition Parameters
Date_ 20171216
Time 17.36
INSTRUM FOURIER300
PROBHD 5 mm DUL 13C-1
PULPROG zg30
TD 65536
SOLVENT DMSO
NS 16
DS 2
SWH 6103.516 Hz
FIDRES 0.093132 Hz
AQ 5.3687091 sec
RG 53.7088
DW 81.920 usec
DE 6.50 usec
TE 300.0 K
D1 1.00000000 sec
TD0 1

======== CHANNEL f1 ========
SFO1 300.1618536 MHz
NUC1 1H
P1 13.50 usec
PLW1 9.30000019 W

F2 - Processing parameters
SI 65536
SF 300.1600023 MHz
WDW EM
SSB 0
LB 0.30 Hz
GB 0
PC 1.00

N-(4-methylphenyl)succinimide (4g)

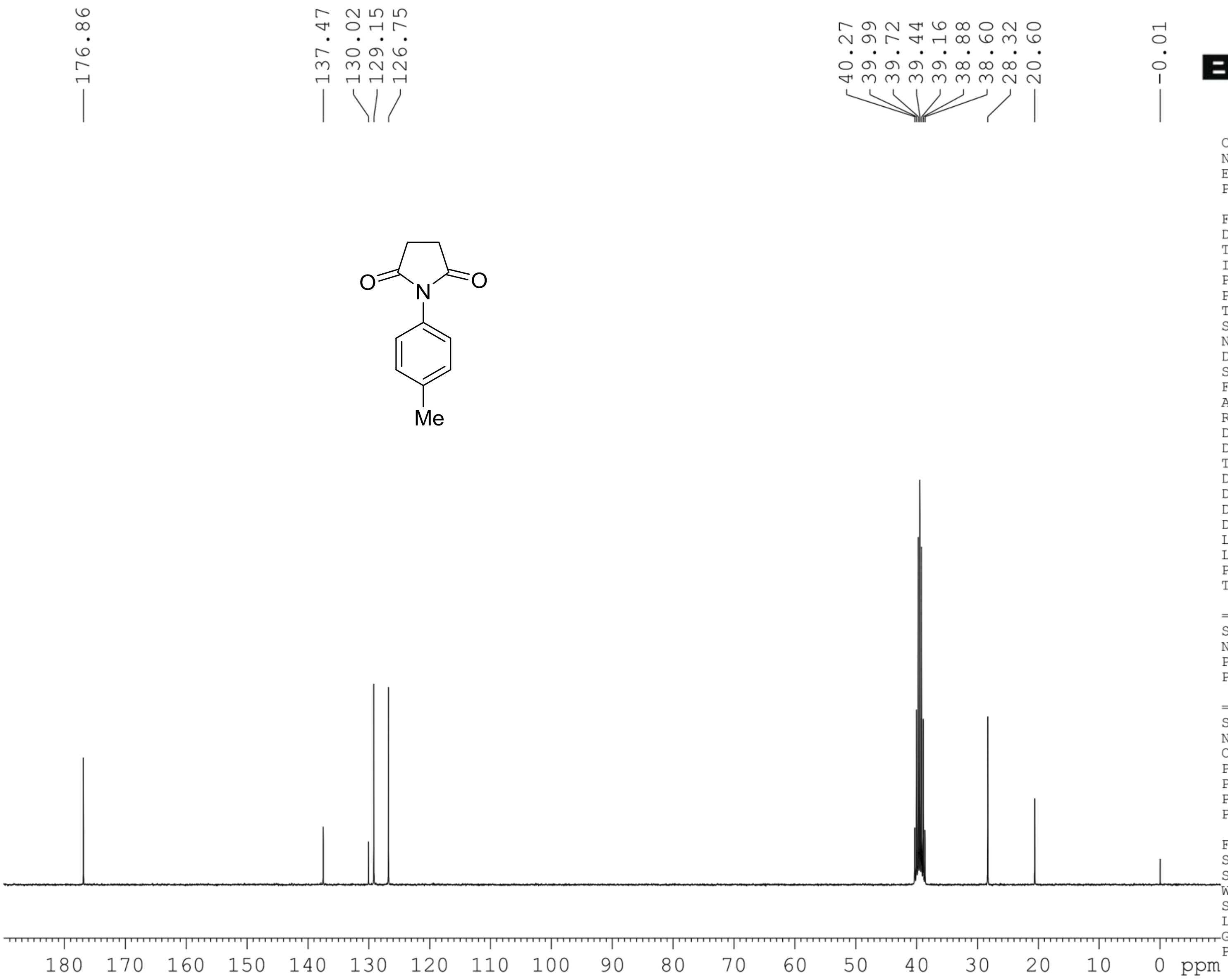

BRUKER

Current Data Parameters
NAME LY124
EXPNO 2
PROCNO 1

F2 - Acquisition Parameters
Date_ 20171216
Time 18.14
INSTRUM FOURIER300
PROBHD 5 mm DUL 13C-1
PULPROG zgpg30
TD 65536
SOLVENT DMSO
NS 4096
DS 4
SWH 24414.063 Hz
FIDRES 0.372529 Hz
AQ 1.3421773 sec
RG 501.187
DW 20.480 usec
DE 6.50 usec
TE 300.1 K
D1 2.00000000 sec
D11 0.03000000 sec
D31 0.00001500 sec
D40 0.00439029 sec
L4 37
L5 53
P32 98.00 usec
TD0 4

======== CHANNEL f1 ========
SFO1 75.4828392 MHz
NUC1 13C
P1 15.00 usec
PLW1 22.00000000 W

======== CHANNEL f2 ========
SFO2 300.1612006 MHz
NUC2 1H
CPDPRG[2 waltz16
PCPD2 98.00 usec
PLW2 9.30000019 W
PLW12 0.29359001 W
PLW13 0.20359001 W

F2 - Processing parameters
SI 32768
SF 75.4753352 MHz
WDW EM
SSB 0
LB 1.00 Hz
GB 0
PC 1.40

N-(4-isopropylphenyl)succinimide (4h)

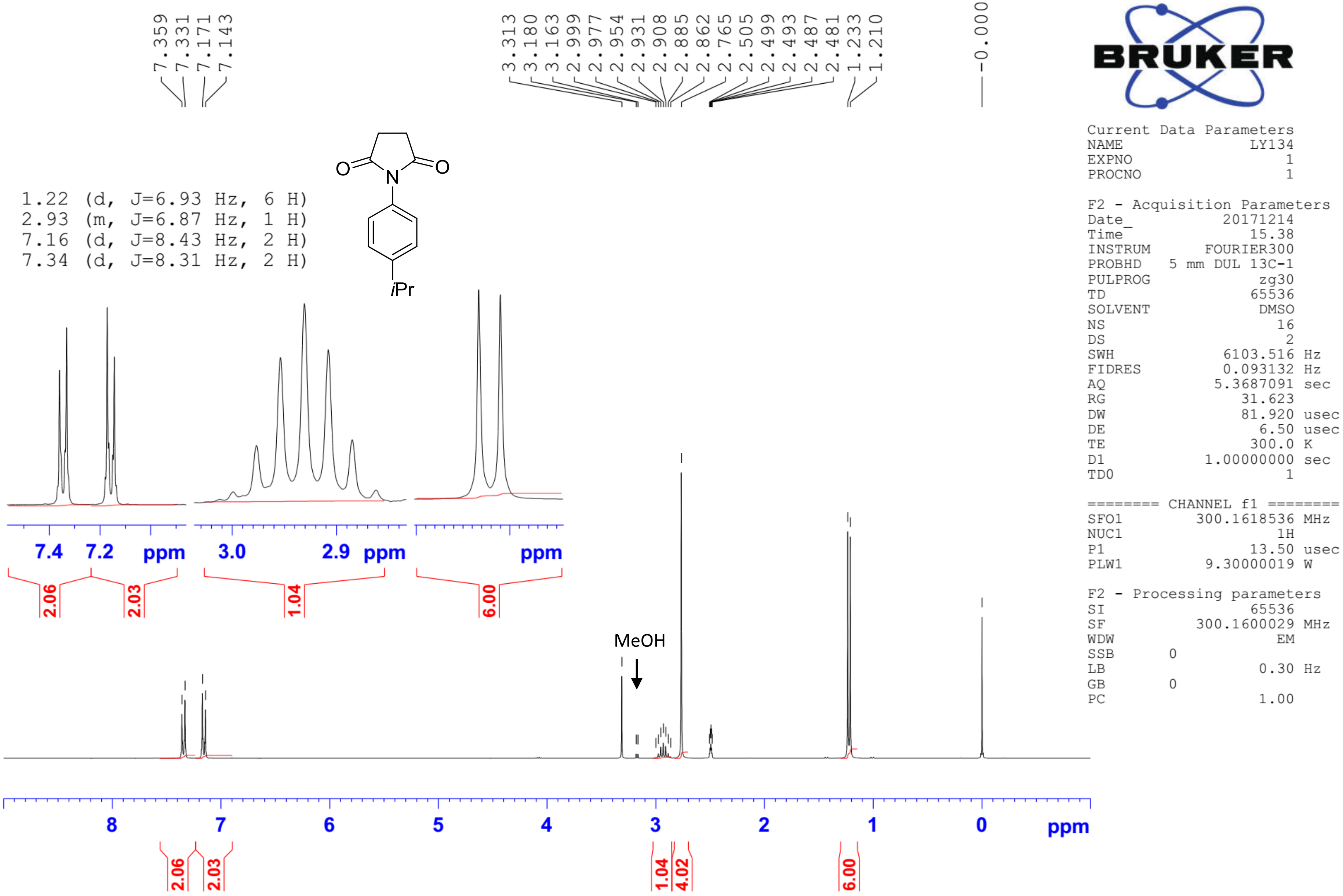

***N*-(4-isopropylphenyl)succinimide (4h)**

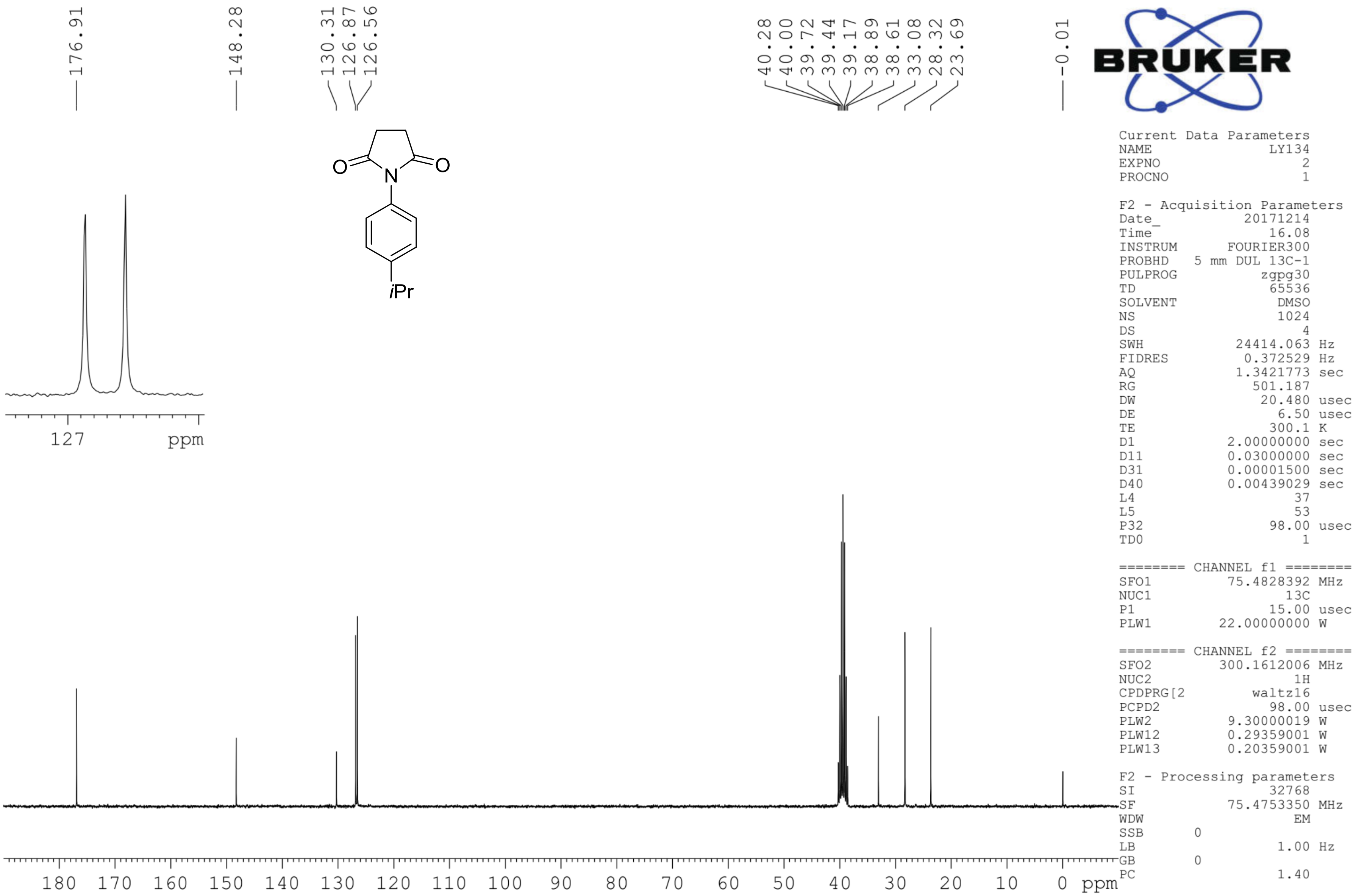

N-(3-methoxyphenyl)succinimide (4i)

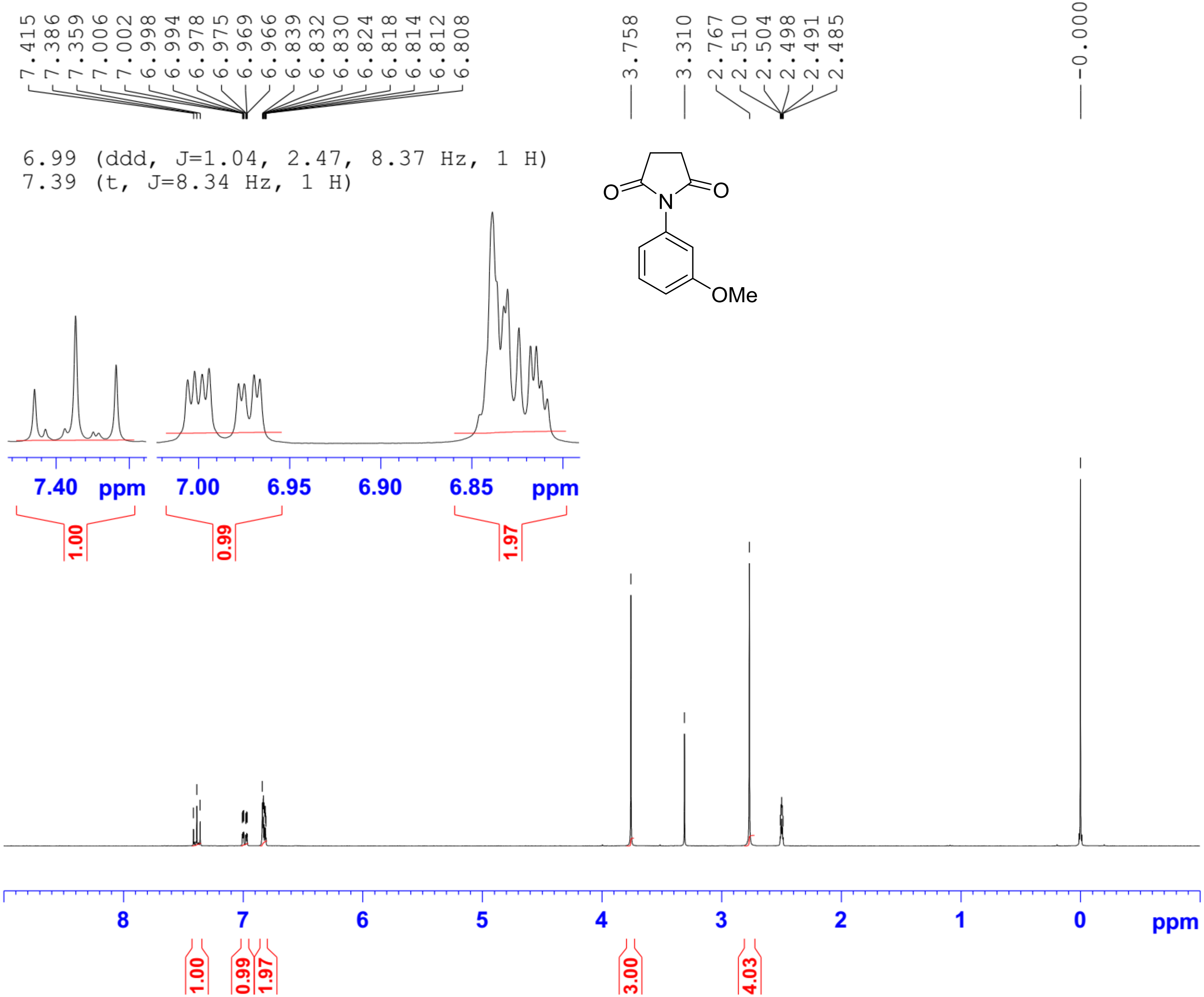

Current Data Parameters
NAME LY141
EXPNO 1
PROCNO 1

F2 - Acquisition Parameters
Date_ 20171225
Time 17.08
INSTRUM FOURIER300
PROBHD 5 mm DUL 13C-1
PULPROG zg30
TD 65536
SOLVENT DMSO
NS 16
DS 2
SWH 6103.516 Hz
FIDRES 0.093132 Hz
AQ 5.3687091 sec
RG 79.5788
DW 81.920 usec
DE 6.50 usec
TE 300.0 K
D1 1.00000000 sec
TD0 1

======== CHANNEL f1 ========
SFO1 300.1618536 MHz
NUC1 1H
P1 13.50 usec
PLW1 9.30000019 W

F2 - Processing parameters
SI 65536
SF 300.1600014 MHz
WDW EM
SSB 0
LB 0.30 Hz
GB 0
PC 1.00

N-(3-methoxyphenyl)succinimide (4i)

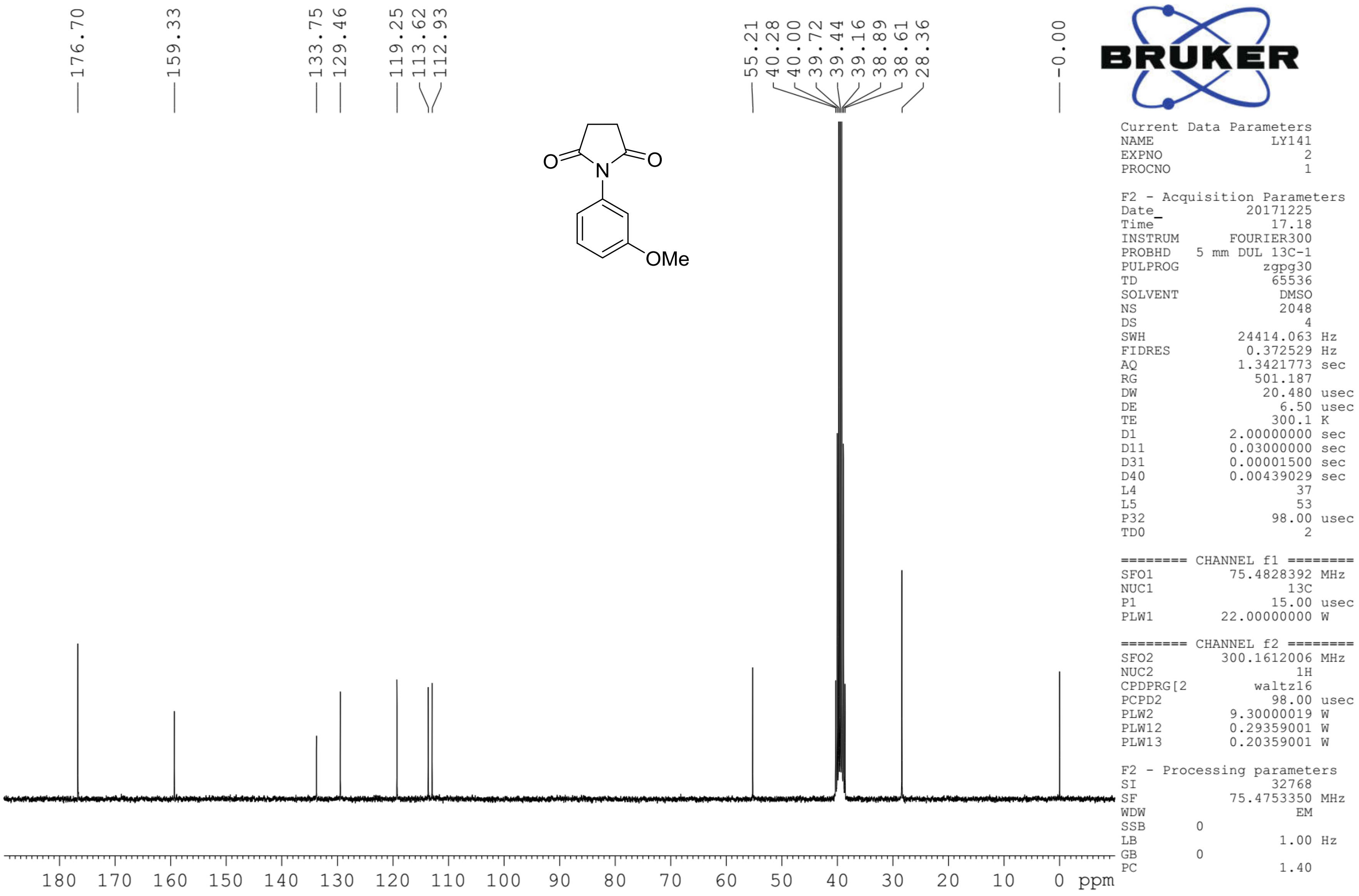

N-(4-methoxyphenyl)succinimide (4j)

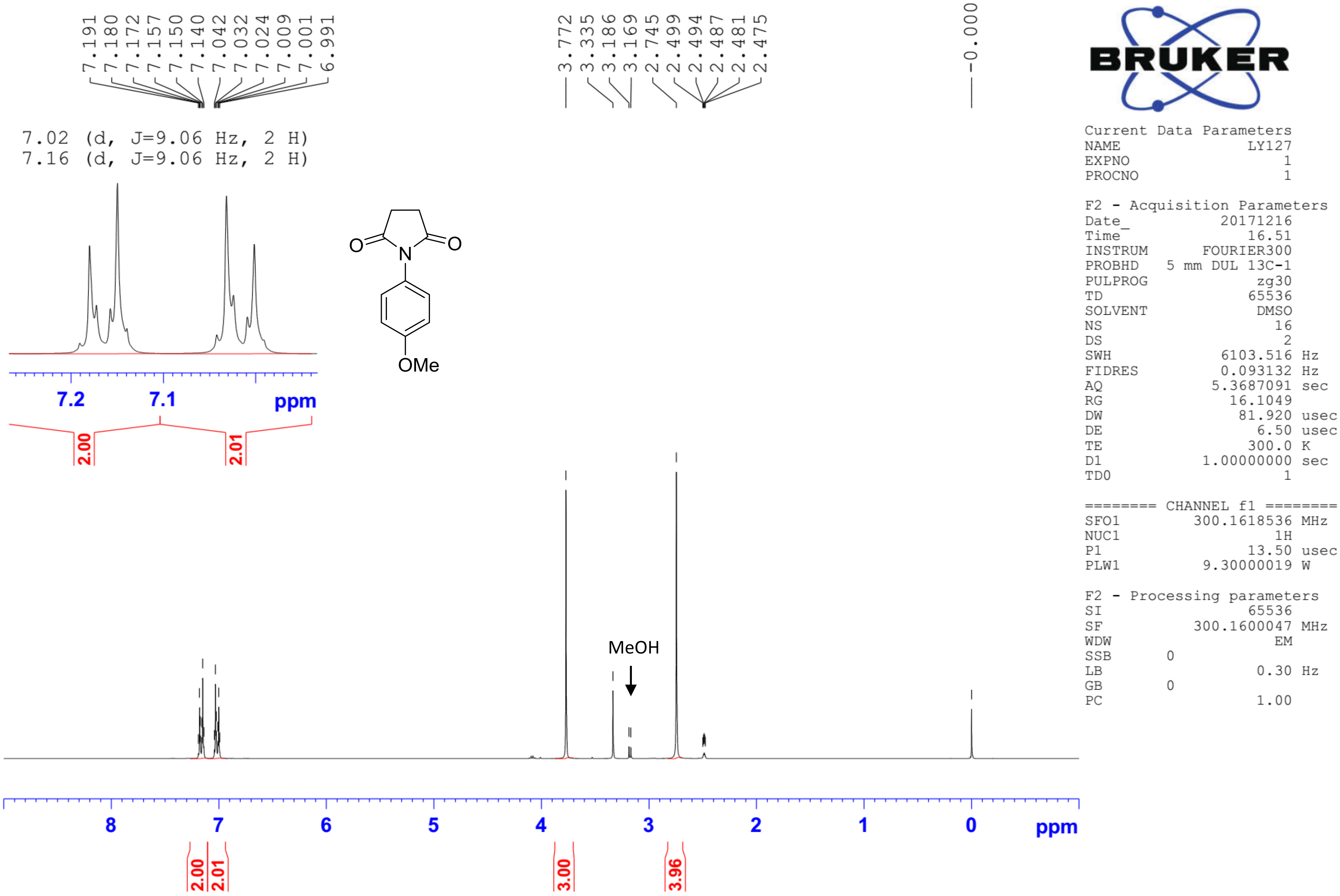

N-(4-methoxyphenyl)succinimide (4j)

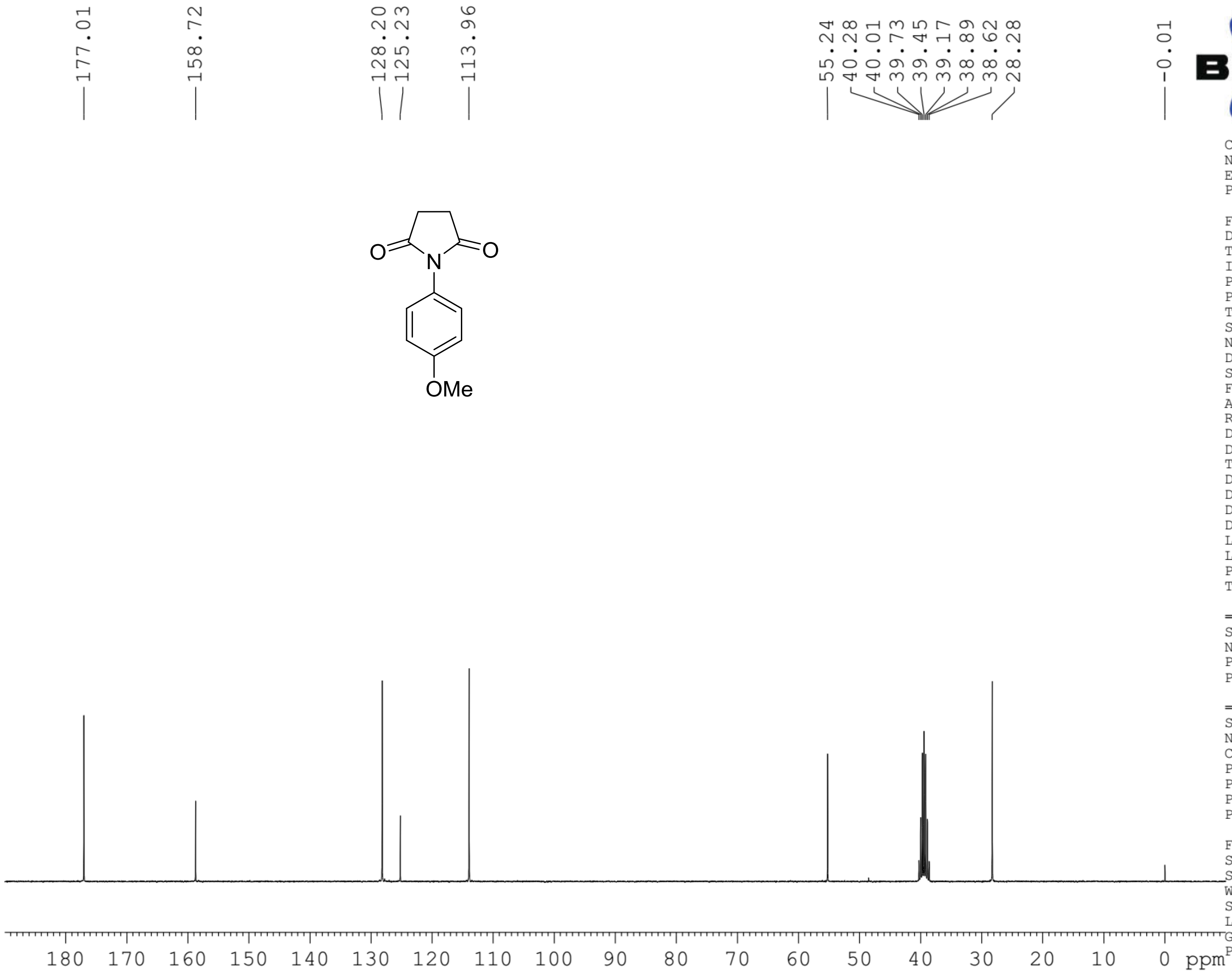

Current Data Parameters	
NAME	LY127
EXPNO	2
PROCNO	1

F2 - Acquisition Parameters	
Date_	20171211
Time	18.40
INSTRUM	FOURIER300
PROBHD	5 mm DUL 13C-1
PULPROG	zgpg30
TD	65536
SOLVENT	DMSO
NS	1024
DS	4
SWH	24414.063 Hz
FIDRES	0.372529 Hz
AQ	1.3421773 sec
RG	501.187
DW	20.480 usec
DE	6.50 usec
TE	300.1 K
D1	2.00000000 sec
D11	0.03000000 sec
D31	0.00001500 sec
D40	0.00439029 sec
L4	37
L5	53
P32	98.00 usec
TD0	1

======== CHANNEL f1 ========	
SFO1	75.4828392 MHz
NUC1	13C
P1	15.00 usec
PLW1	22.00000000 W

======== CHANNEL f2 ========	
SFO2	300.1612006 MHz
NUC2	1H
CPDPRG[2	waltz16
PCPD2	98.00 usec
PLW2	9.30000019 W
PLW12	0.29359001 W
PLW13	0.20359001 W

F2 - Processing parameters	
SI	32768
SF	75.4753336 MHz
WDW	EM
SSB	0
LB	1.00 Hz
GB	0
PC	1.40

N-(4-(N-acetamide)phenyl)succinimide (4k)

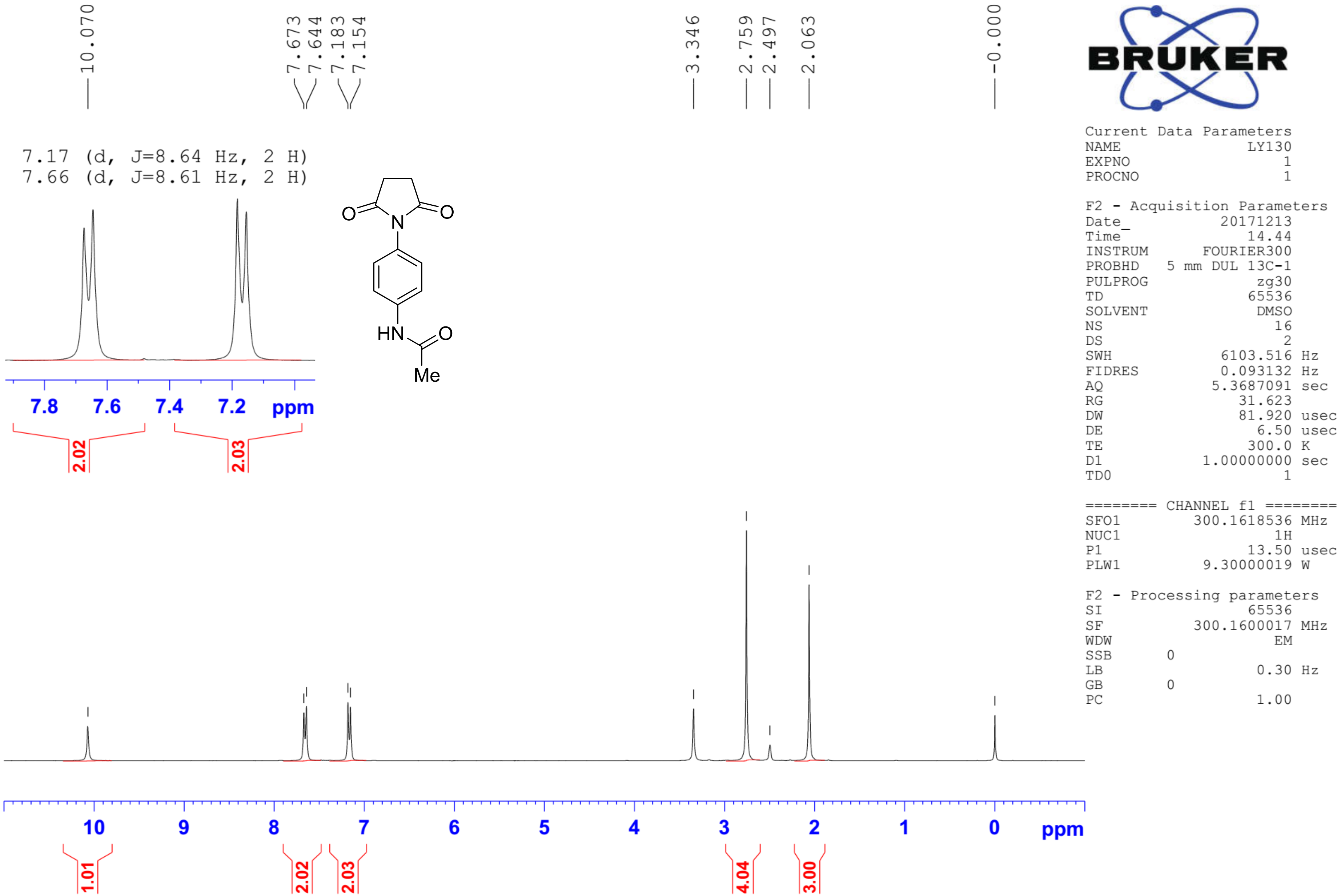

N-(4-(N-acetamide)phenyl)succinimide (4k)

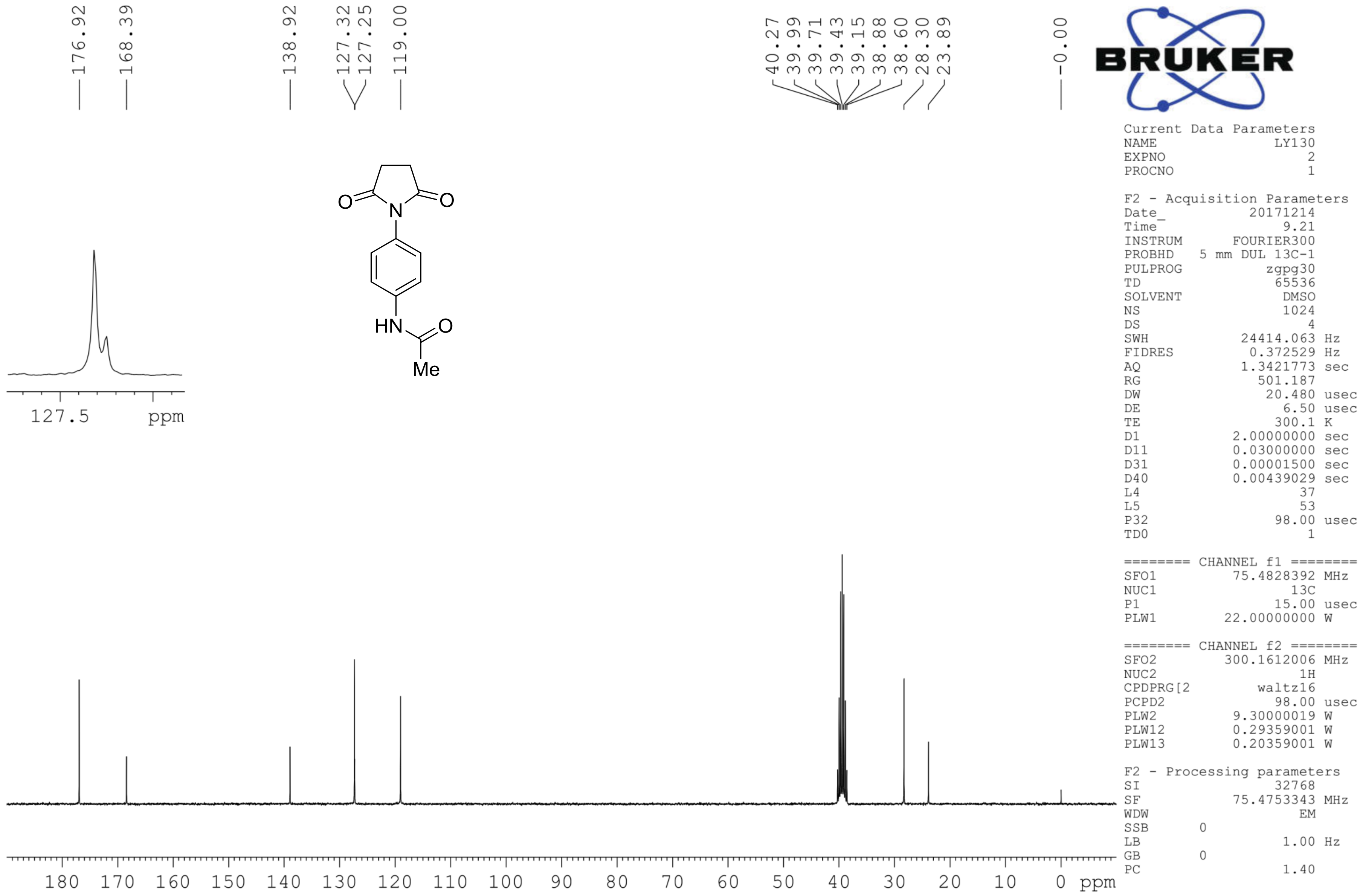

^{1}H and ^{13}C NMR spectra of 3-(5-amino-1*H*-1,2,4-triazol-3-yl)propanamides 5

3-(5-Amino-1H-1,2,4-triazol-3-yl)-1-morpholinopropan-1-one (5a)

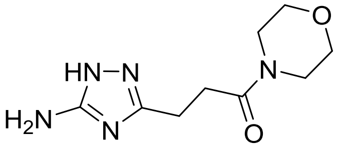

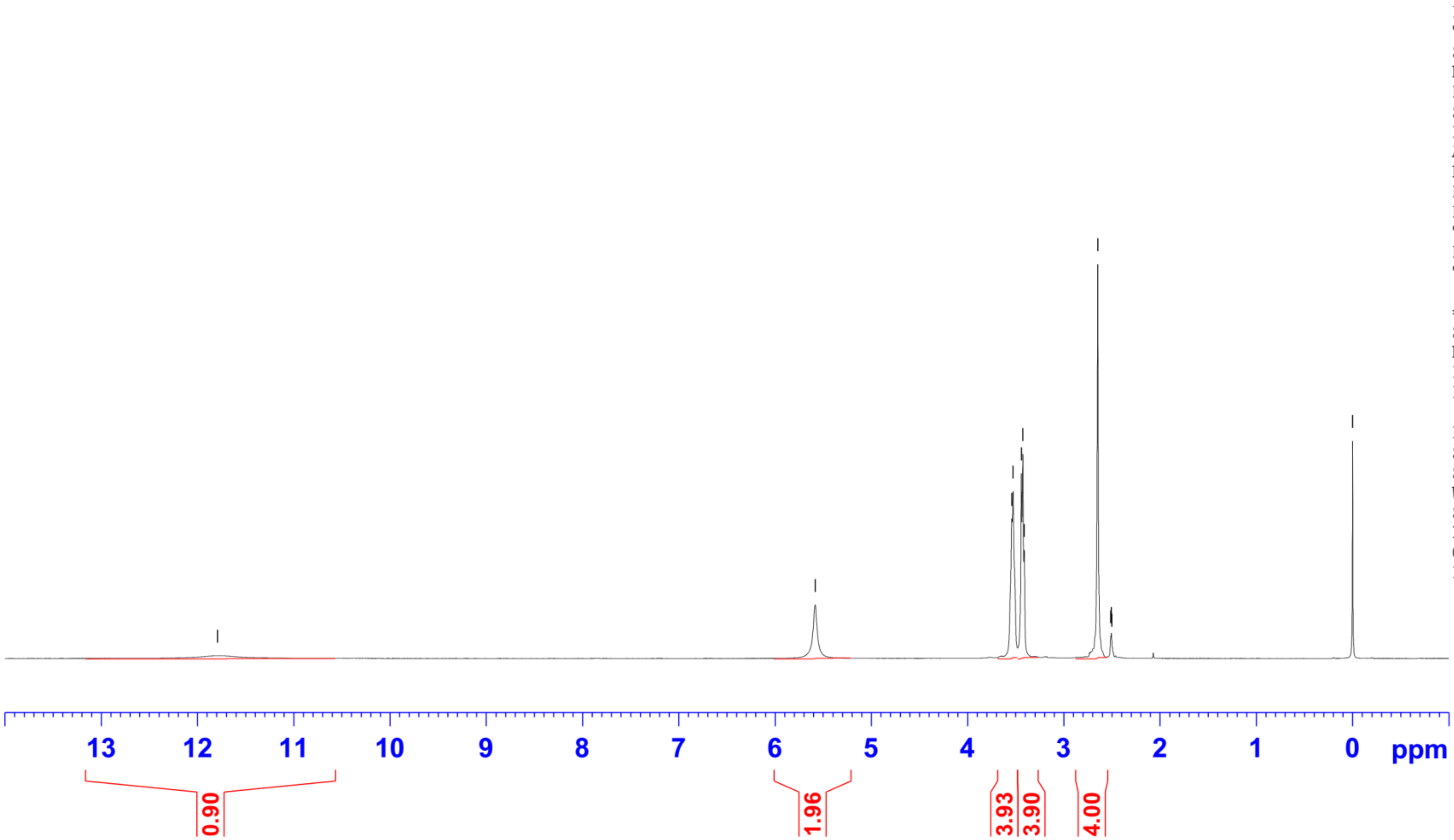

Current Data Parameters
NAME LY37
EXPNO 1
PROCNO 1

F2 - Acquisition Parameters
Date_ 20170707
Time 17.54
INSTRUM FOURIER300
PROBHD 5 mm DUL 13C-1
PULPROG zg30
TD 65536
SOLVENT DMSO
NS 16
DS 2
SWH 6103.516 Hz
FIDRES 0.093132 Hz
AQ 5.3687091 sec
RG 17.5543
DW 81.920 usec
DE 6.50 usec
TE 300.2 K
D1 1.00000000 sec
TD0 1

======== CHANNEL f1 ========
SFO1 300.1618536 MHz
NUC1 1H
P1 13.50 usec
PLW1 9.30000019 W

F2 - Processing parameters
SI 65536
SF 300.1599984 MHz
WDW EM
SSB 0
LB 0.30 Hz
GB 0
PC 1.00

3-(5-Amino-1H-1,2,4-triazol-3-yl)-1-morpholinopropan-1-one (5a)

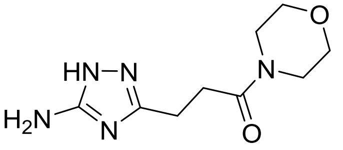

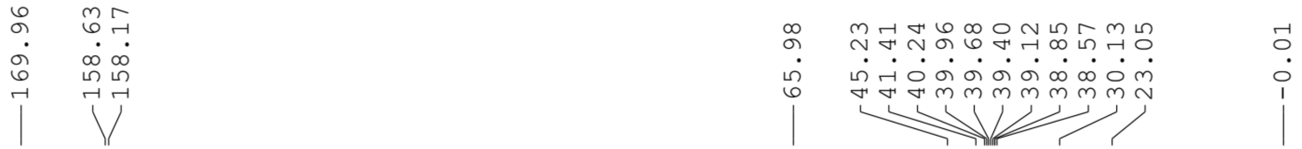

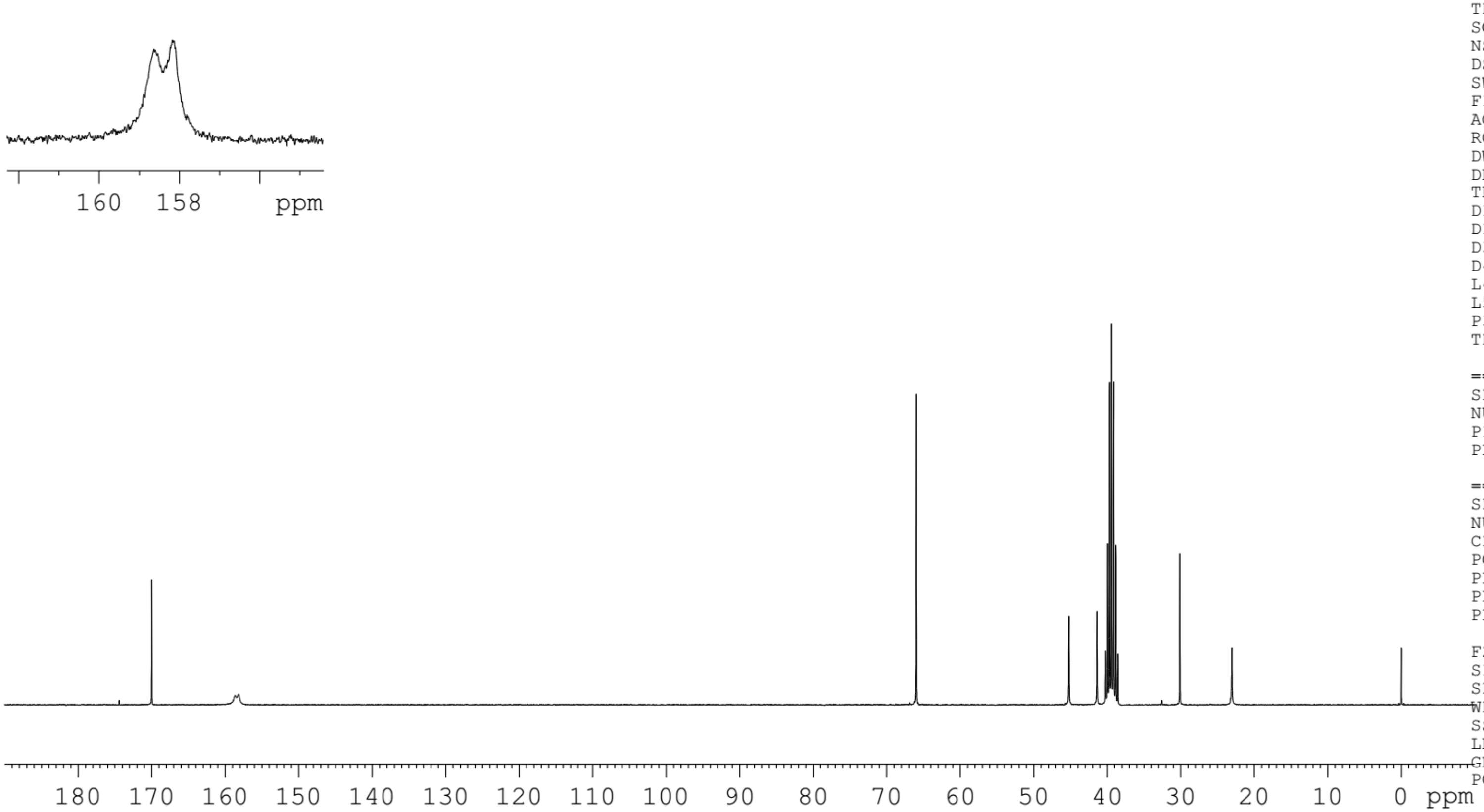

Current Data Parameters
NAME LY37
EXPNO 2
PROCNO 1

F2 - Acquisition Parameters
Date_ 20170707
Time 18.08
INSTRUM FOURIER300
PROBHD 5 mm DUL 13C-1
PULPROG zgpg30
TD 65536
SOLVENT DMSO
NS 14336
DS 4
SWH 24414.063 Hz
FIDRES 0.372529 Hz
AQ 1.3421773 sec
RG 501.187
DW 20.480 usec
DE 6.50 usec
TE 300.2 K
D1 2.00000000 sec
D11 0.03000000 sec
D31 0.00001500 sec
D40 0.00439029 sec
L4 37
L5 53
P32 98.00 usec
TD0 14

======== CHANNEL f1 ========
SFO1 75.4828392 MHz
NUC1 13C
P1 15.00 usec
PLW1 22.00000000 W

======== CHANNEL f2 ========
SFO2 300.1612006 MHz
NUC2 1H
CPDPRG[2 waltz16
PCPD2 98.00 usec
PLW2 9.30000019 W
PLW12 0.29359001 W
PLW13 0.20359001 W

F2 - Processing parameters
SI 32768
SF 75.4753336 MHz
WDW EM
SSB 0
LB 1.00 Hz
GB 0
PC 1.40

3-(5-Amino-1H-1,2,4-triazol-3-yl)-1-(piperidin-1-yl)propan-1-one (5b)

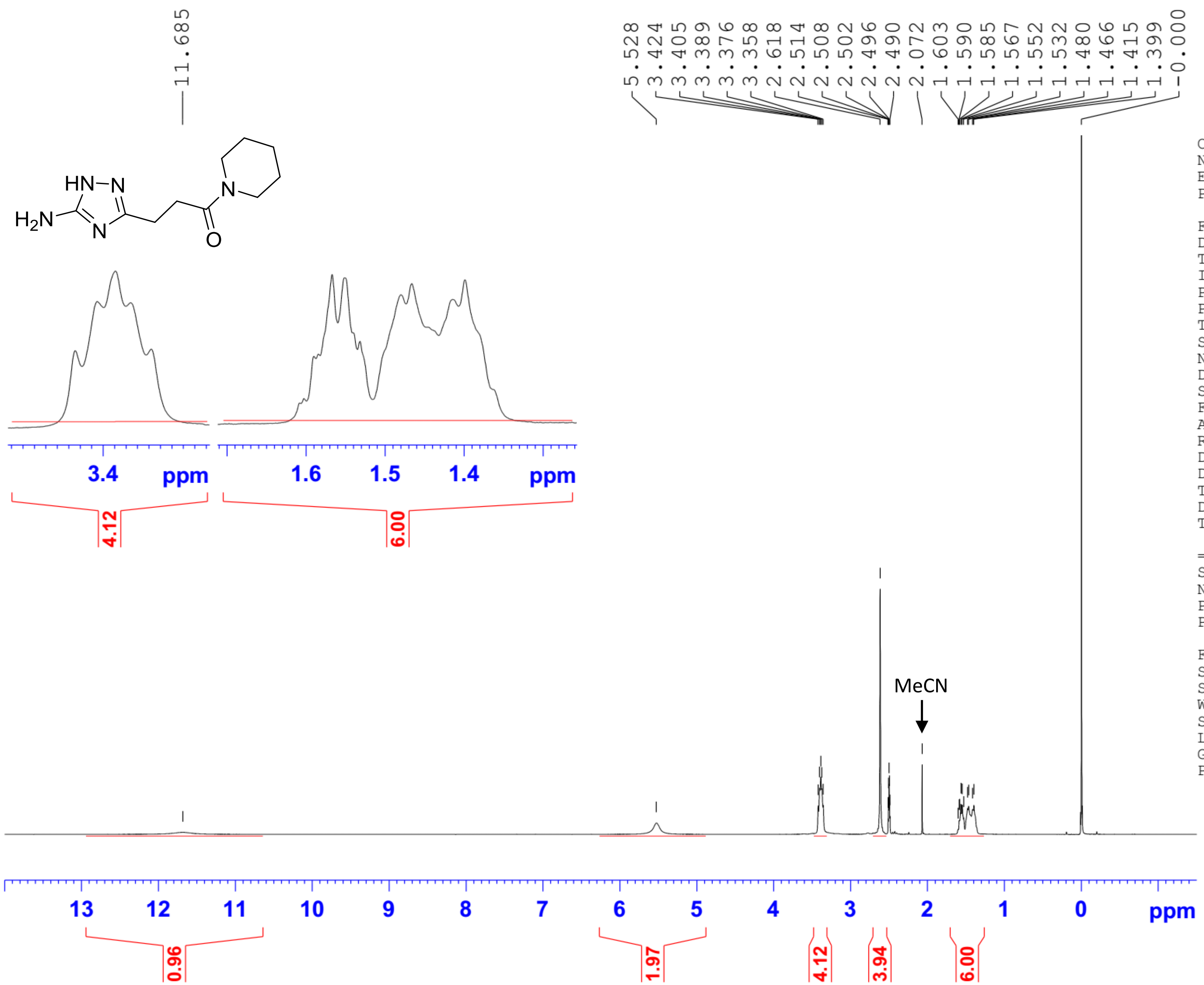

Current Data Parameters
NAME LY88
EXPNO 1
PROCNO 1

F2 - Acquisition Parameters
Date_ 20171011
Time_ 16.14
INSTRUM FOURIER300
PROBHD 5 mm DUL 13C-1
PULPROG zg30
TD 65536
SOLVENT DMSO
NS 16
DS 2
SWH 6103.516 Hz
FIDRES 0.093132 Hz
AQ 5.3687091 sec
RG 31.623
DW 81.920 usec
DE 6.50 usec
TE 300.0 K
D1 1.00000000 sec
TD0 1

======== CHANNEL f1 ========
SFO1 300.1618536 MHz
NUC1 1H
P1 13.50 usec
PLW1 9.30000019 W

F2 - Processing parameters
SI 65536
SF 300.1600001 MHz
WDW EM
SSB 0
LB 0.30 Hz
GB 0
PC 1.00

3-(5-Amino-1H-1,2,4-triazol-3-yl)-1-(piperidin-1-yl)propan-1-one (5b)

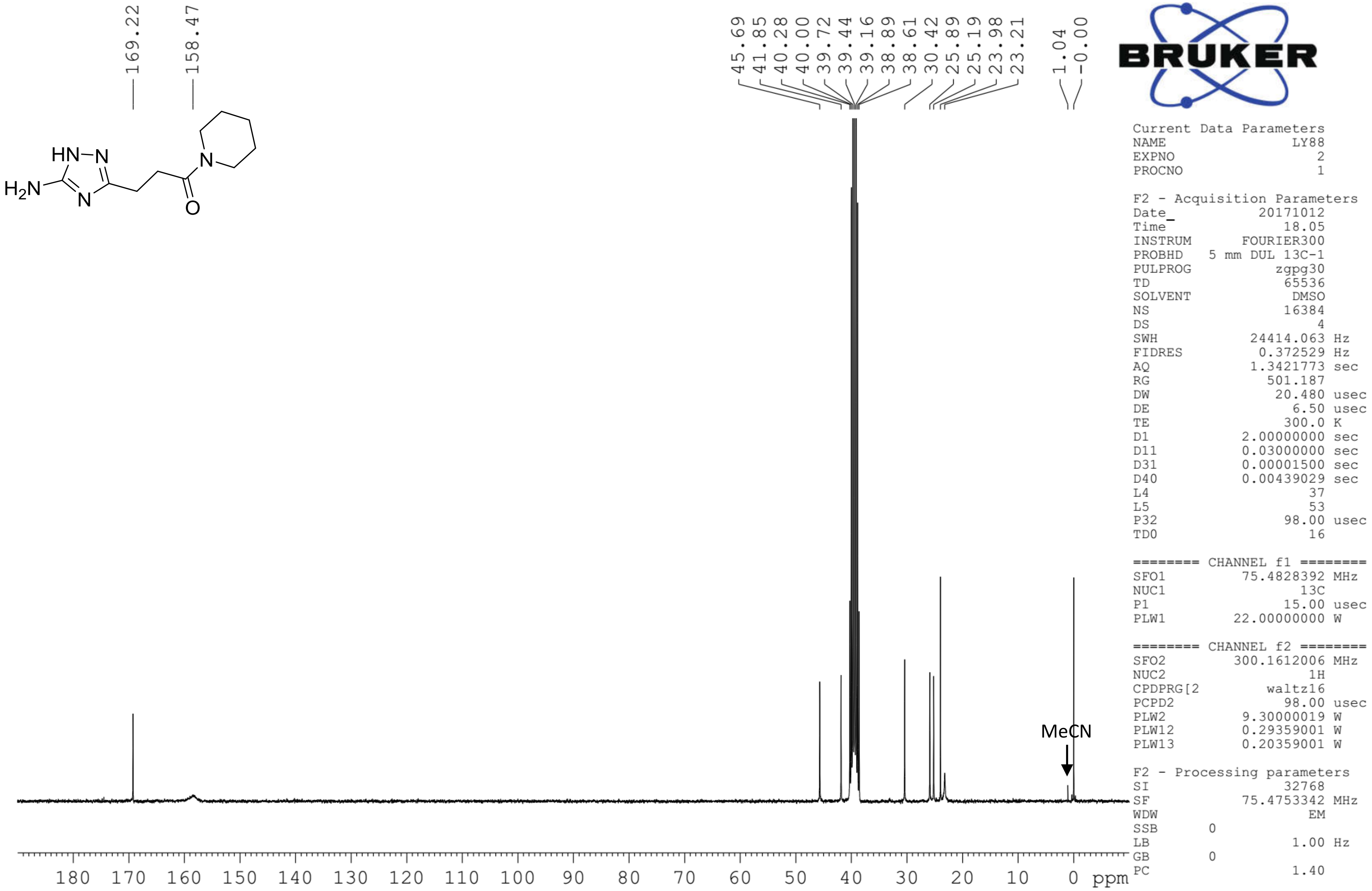

3-(5-Amino-1H-1,2,4-triazol-3-yl)-1-(pyrrolidin-1-yl)propan-1-one (5c)

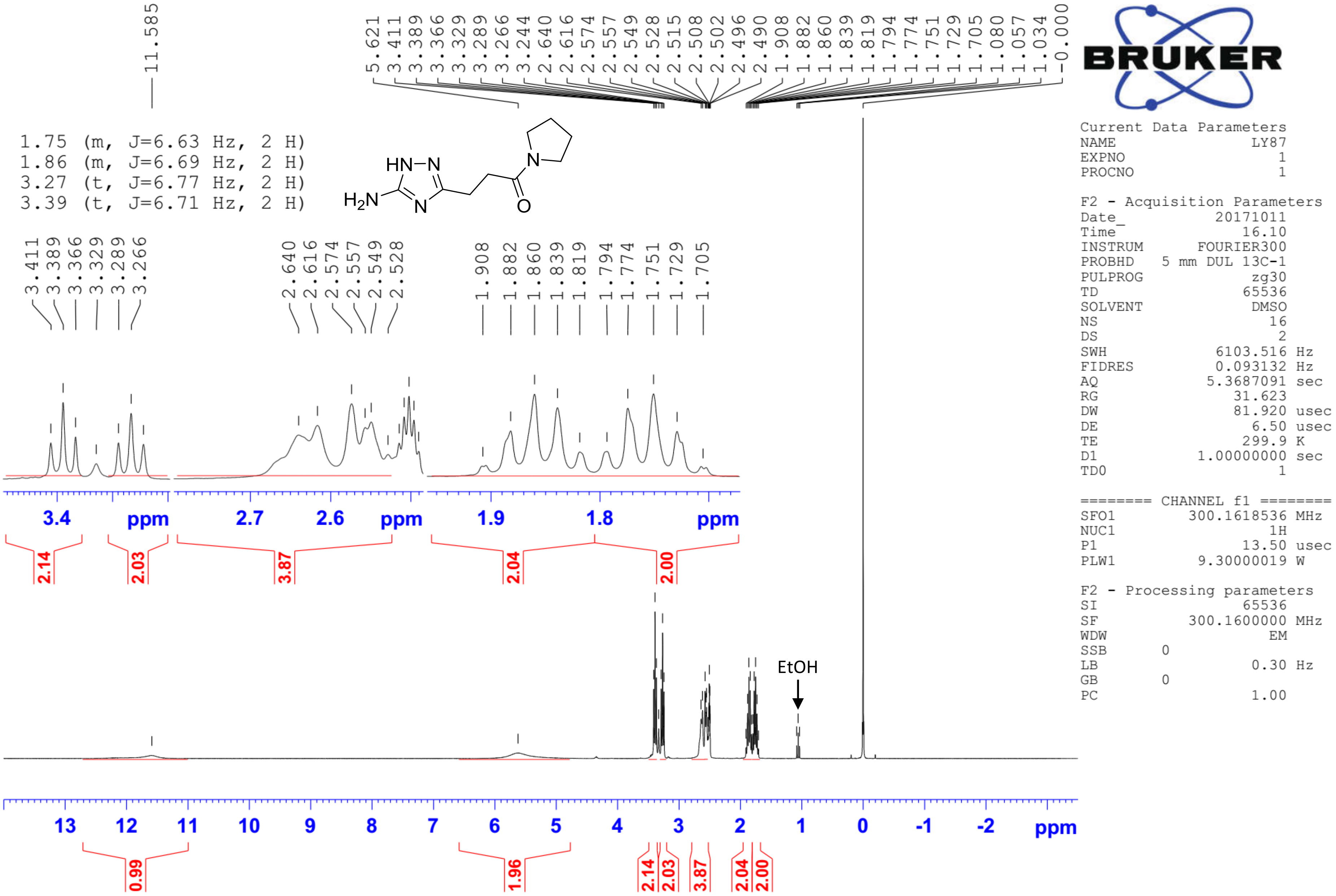

3-(5-Amino-1H-1,2,4-triazol-3-yl)-1-(pyrrolidin-1-yl)propan-1-one (5c)

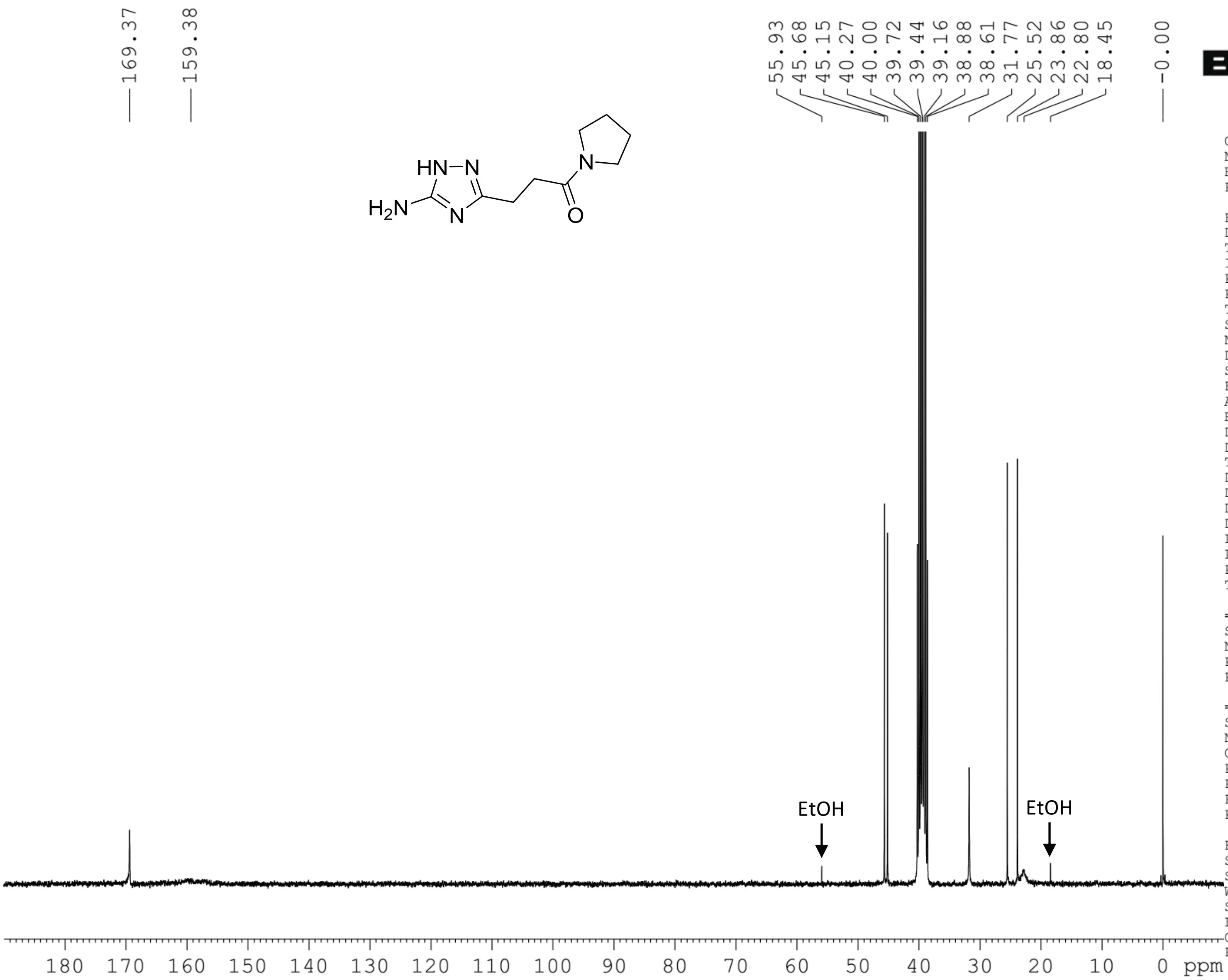

Current Data Parameters
NAME LY87
EXPNO 2
PROCNO 1

F2 - Acquisition Parameters
Date_ 20171011
Time 18.05
INSTRUM FOURIER300
PROBHD 5 mm DUL 13C-1
PULPROG zgpg30
TD 65536
SOLVENT DMSO
NS 16384
DS 4
SWH 24414.063 Hz
FIDRES 0.372529 Hz
AQ 1.3421773 sec
RG 501.187
DW 20.480 usec
DE 6.50 usec
TE 300.0 K
D1 2.00000000 sec
D11 0.03000000 sec
D31 0.00001500 sec
D40 0.00439029 sec
L4 37
L5 53
P32 98.00 usec
TD0 16

======== CHANNEL f1 ========
SFO1 75.4828392 MHz
NUC1 13C
P1 15.00 usec
PLW1 22.00000000 W

======== CHANNEL f2 ========
SFO2 300.1612006 MHz
NUC2 1H
CPDPRG[2 waltz16
PCPD2 98.00 usec
PLW2 9.30000019 W
PLW12 0.29359001 W
PLW13 0.20359001 W

F2 - Processing parameters
SI 32768
SF 75.4753342 MHz
WDW EM
SSB 0
LB 1.00 Hz
GB 0
PC 1.40

3-(5-Amino-1H-1,2,4-triazol-3-yl)-N-(4-benzyl)propanamide (5d)

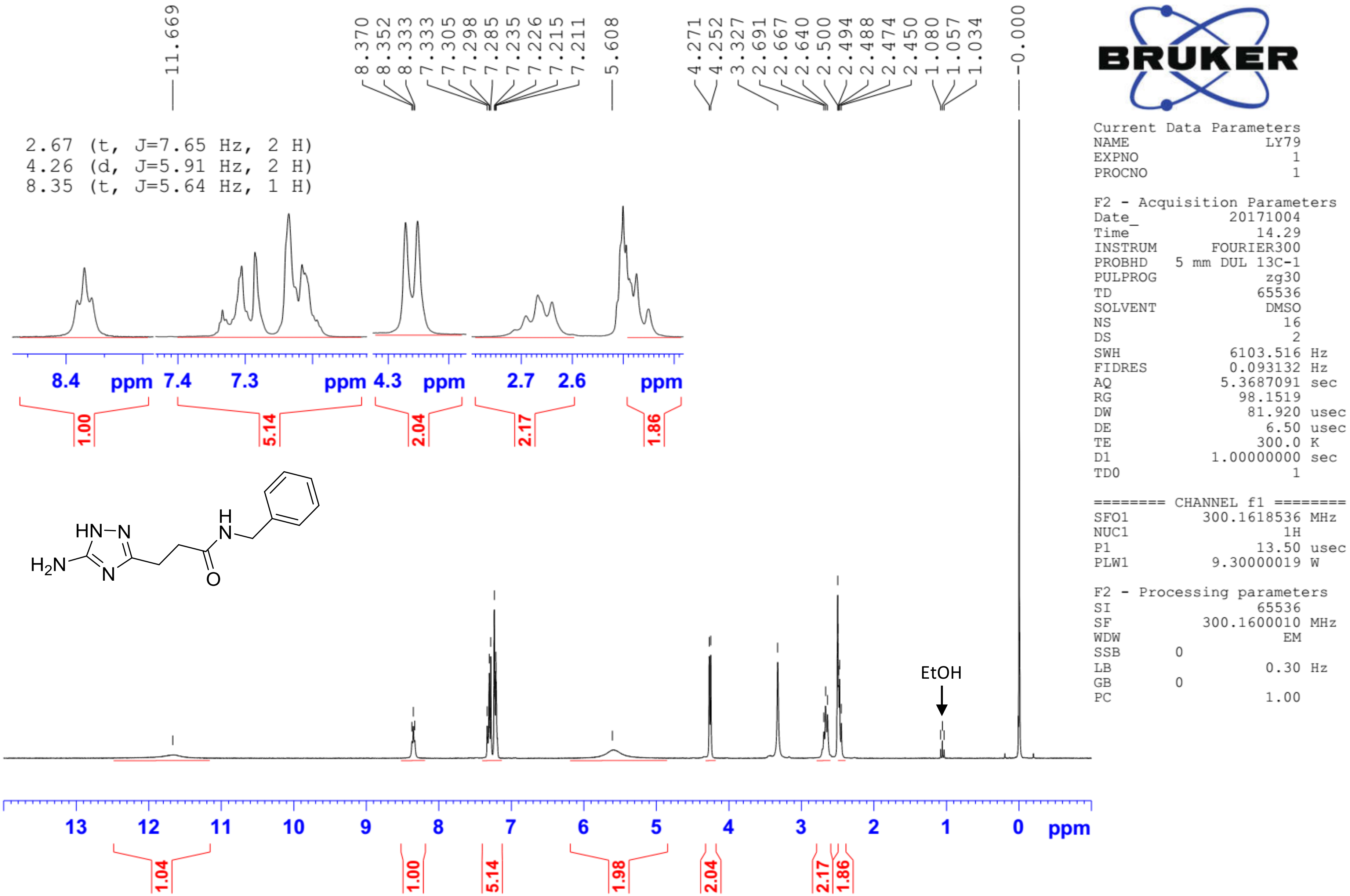

3-(5-Amino-1H-1,2,4-triazol-3-yl)-N-(4-benzyl)propanamide (5d)

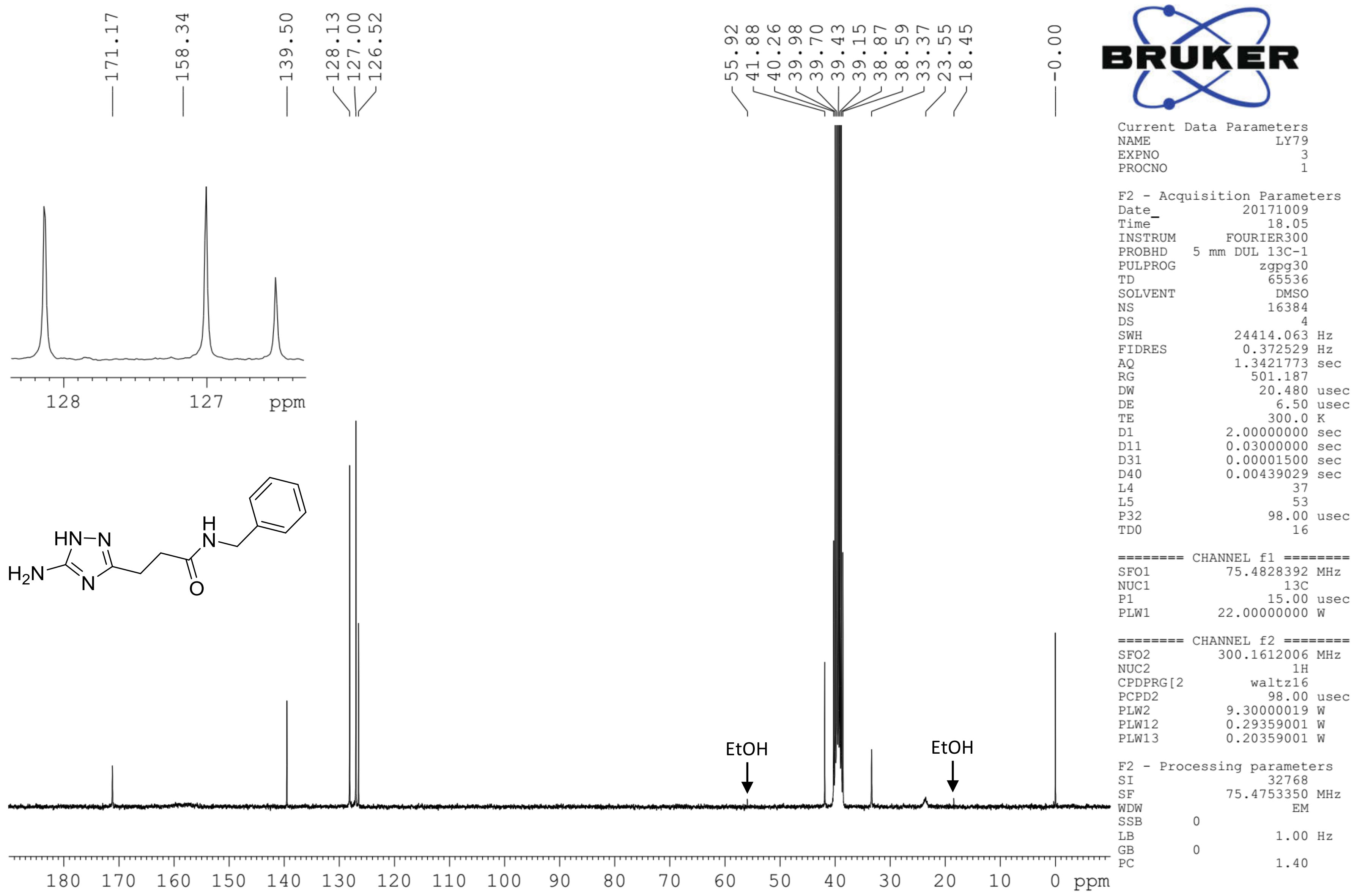

3-(5-Amino-1H-1,2,4-triazol-3-yl)-N-(4-methoxybenzyl)propanamide (5e)

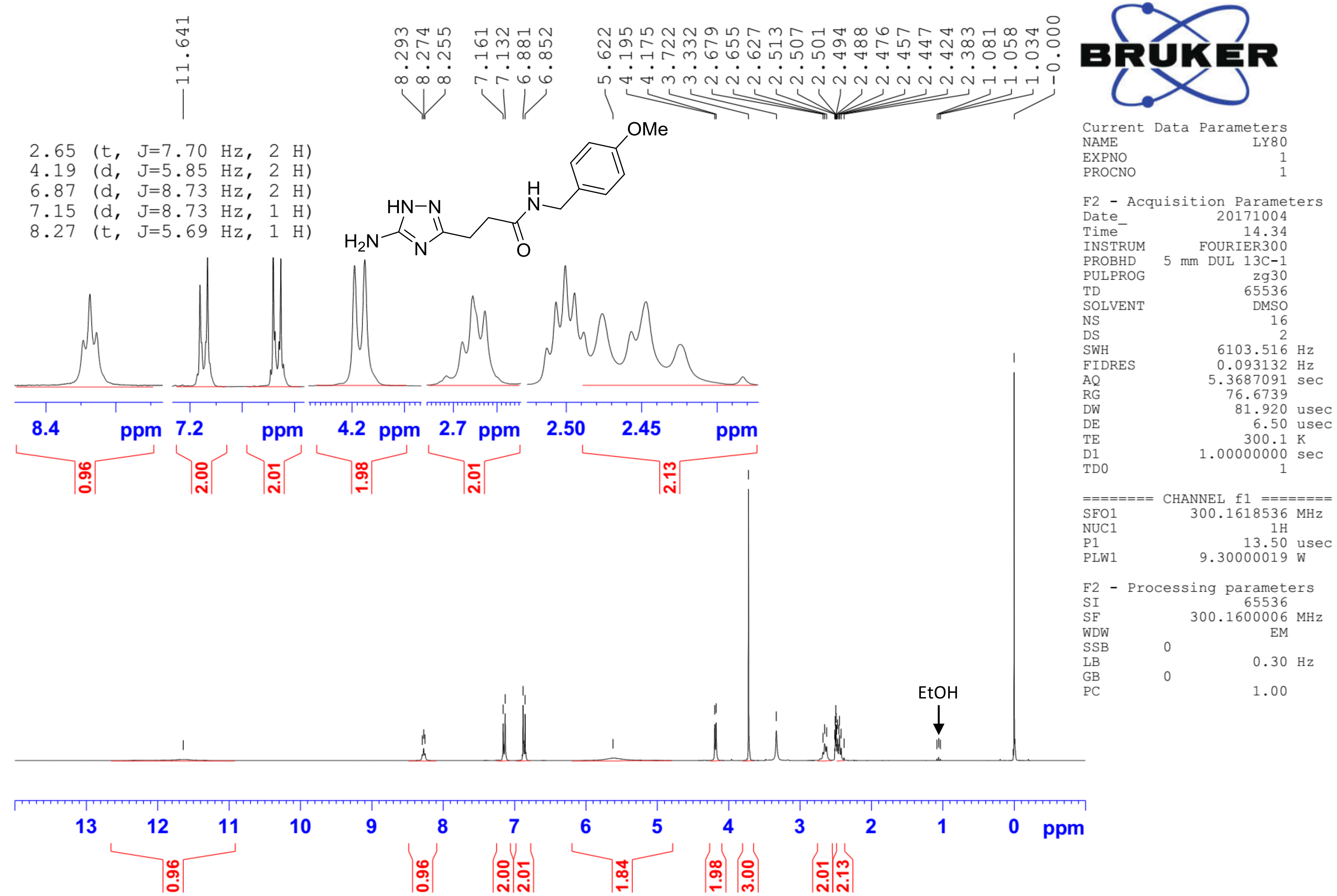

3-(5-Amino-1H-1,2,4-triazol-3-yl)-N-(4-methoxybenzyl)propanamide (5e)

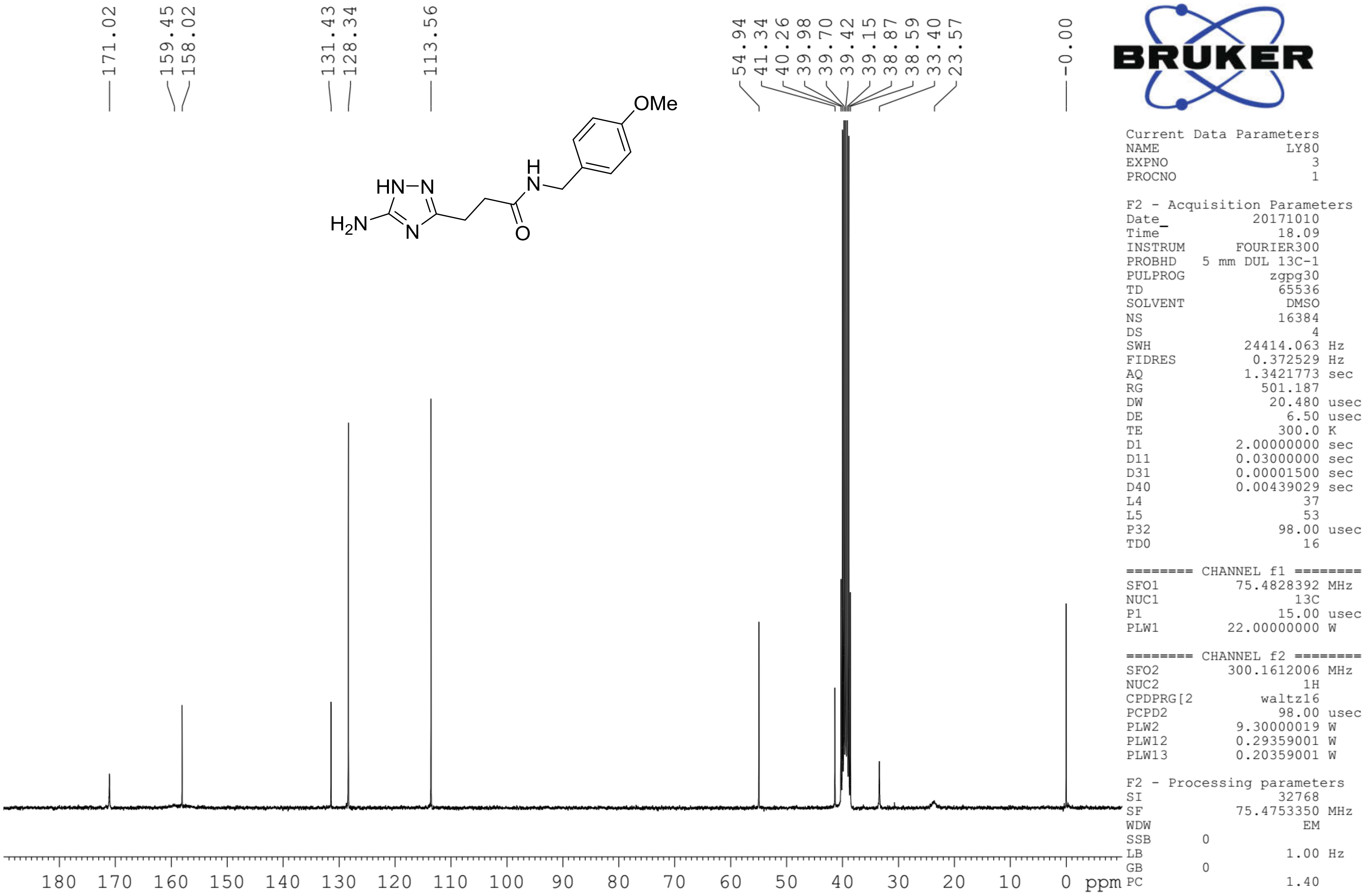

3-(5-Amino-1H-1,2,4-triazol-3-yl)-N-(4-fluorobenzyl)propanamide (5f)

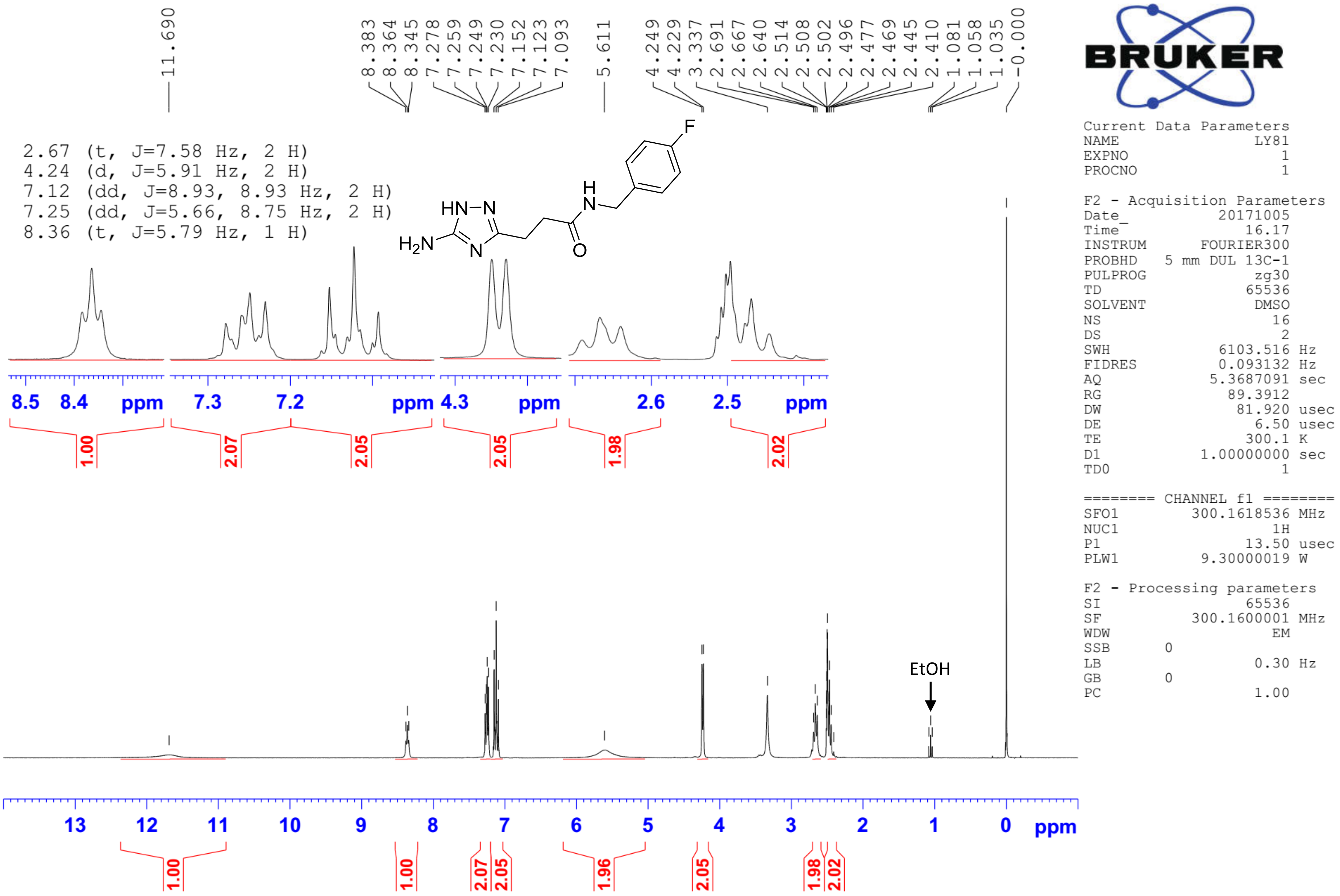

3-(5-Amino-1H-1,2,4-triazol-3-yl)-N-(4-fluorobenzyl)propanamide (5f)

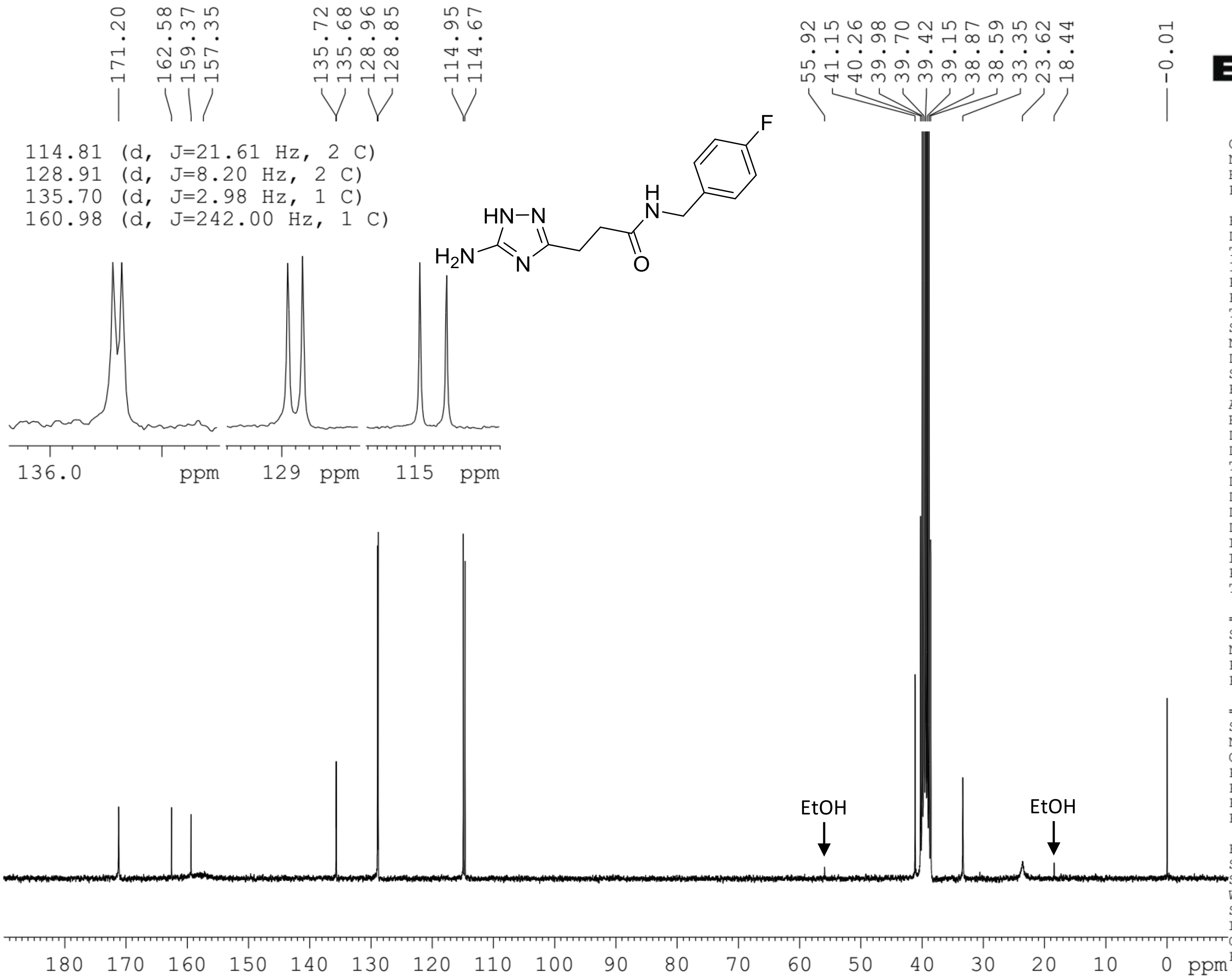

Current Data Parameters
NAME LY81
EXPNO 2
PROCNO 1

F2 - Acquisition Parameters
Date_ 20171005
Time 18.05
INSTRUM FOURIER300
PROBHD 5 mm DUL 13C-1
PULPROG zgpg30
TD 65536
SOLVENT DMSO
NS 15360
DS 4
SWH 24414.063 Hz
FIDRES 0.372529 Hz
AQ 1.3421773 sec
RG 501.187
DW 20.480 usec
DE 6.50 usec
TE 300.1 K
D1 2.00000000 sec
D11 0.03000000 sec
D31 0.00001500 sec
D40 0.00439029 sec
L4 37
L5 53
P32 98.00 usec
TD0 15

======== CHANNEL f1 ========
SFO1 75.4828392 MHz
NUC1 13C
P1 15.00 usec
PLW1 22.00000000 W

======== CHANNEL f2 ========
SFO2 300.1612006 MHz
NUC2 1H
CPDPRG[2 waltz16
PCPD2 98.00 usec
PLW2 9.30000019 W
PLW12 0.29359001 W
PLW13 0.20359001 W

F2 - Processing parameters
SI 32768
SF 75.4753350 MHz
WDW EM
SSB 0
LB 1.00 Hz
GB 0
PC 1.40

3-(5-Amino-1H-1,2,4-triazol-3-yl)-N-(3,4-difluorobenzyl)propanamide (5g)

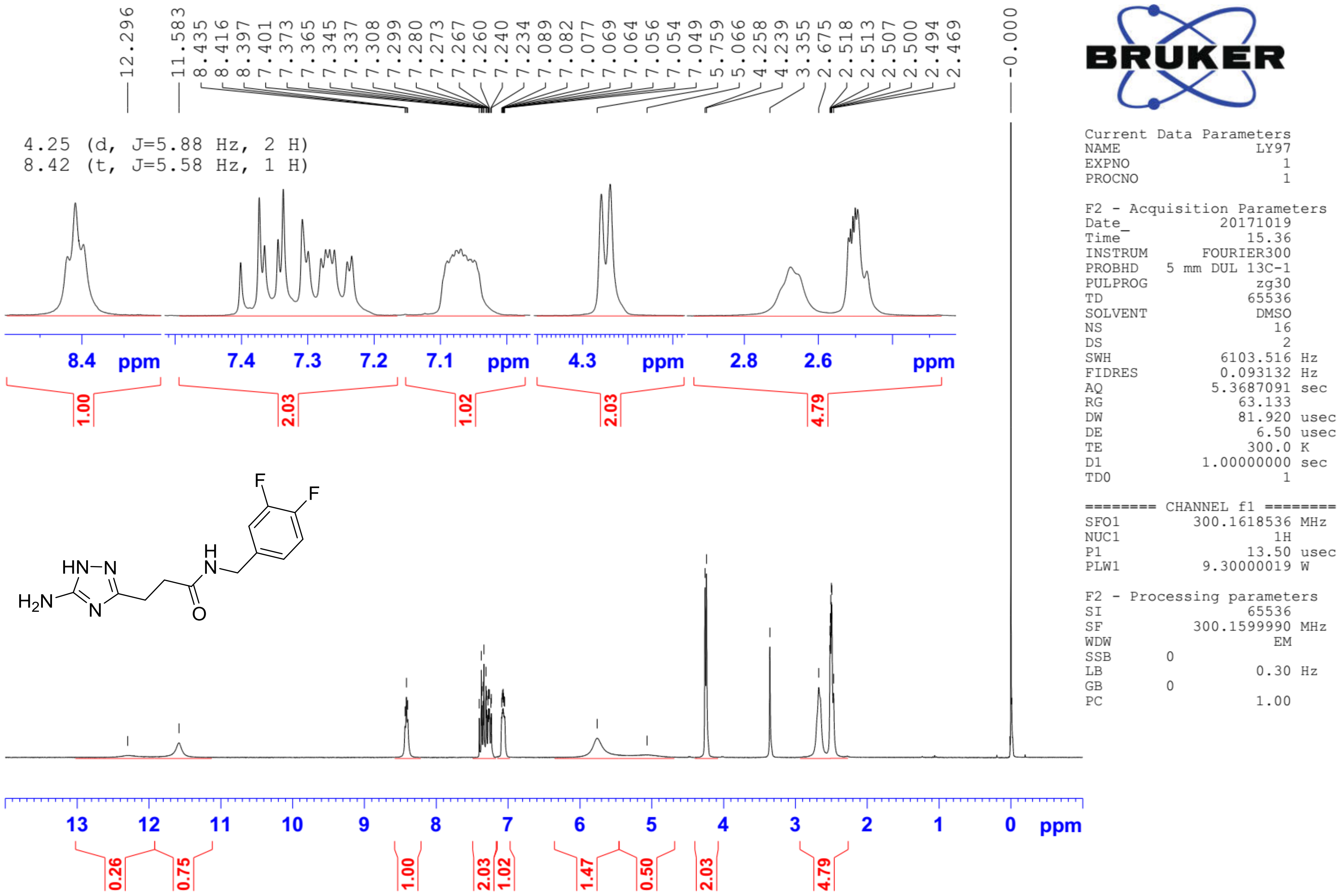

3-(5-Amino-1H-1,2,4-triazol-3-yl)-N-(3,4-difluorobenzyl)propanamide (5g)

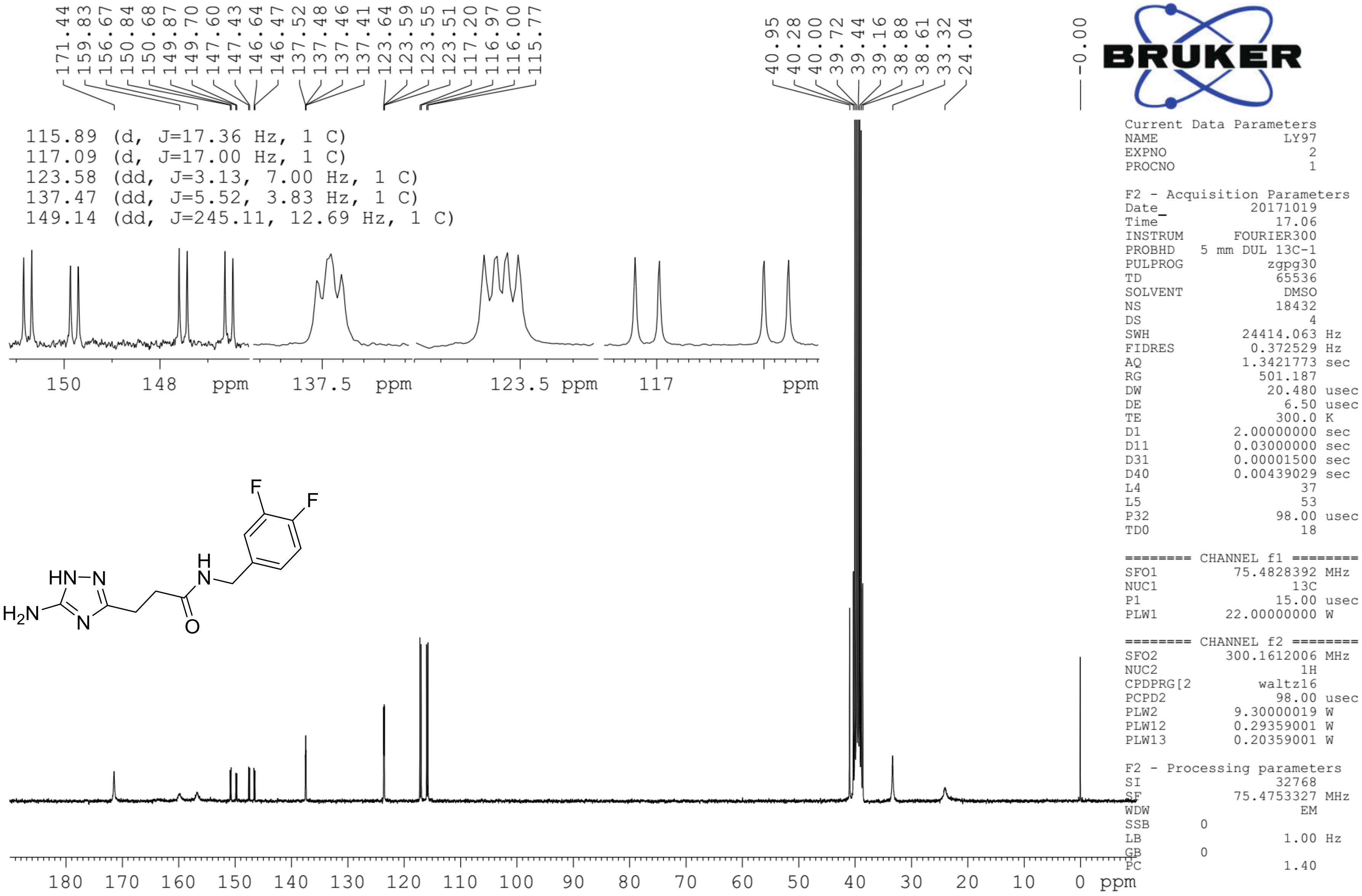

3-(5-Amino-1H-1,2,4-triazol-3-yl)-N-(3-trifluoromethylbenzyl)propanamide (5h)

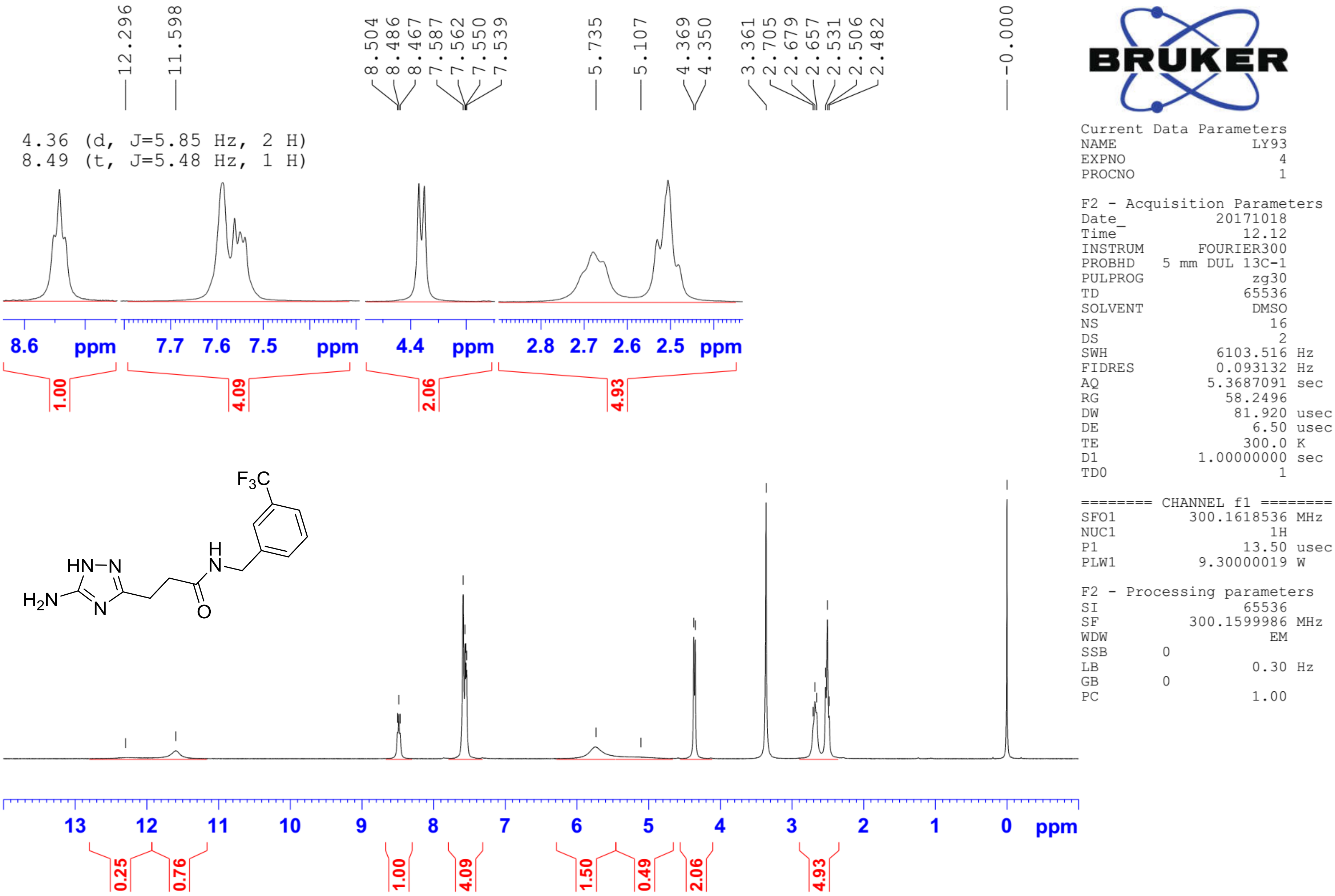

3-(5-Amino-1H-1,2,4-triazol-3-yl)-N-(3-trifluoromethylbenzyl)propanamide (5h)

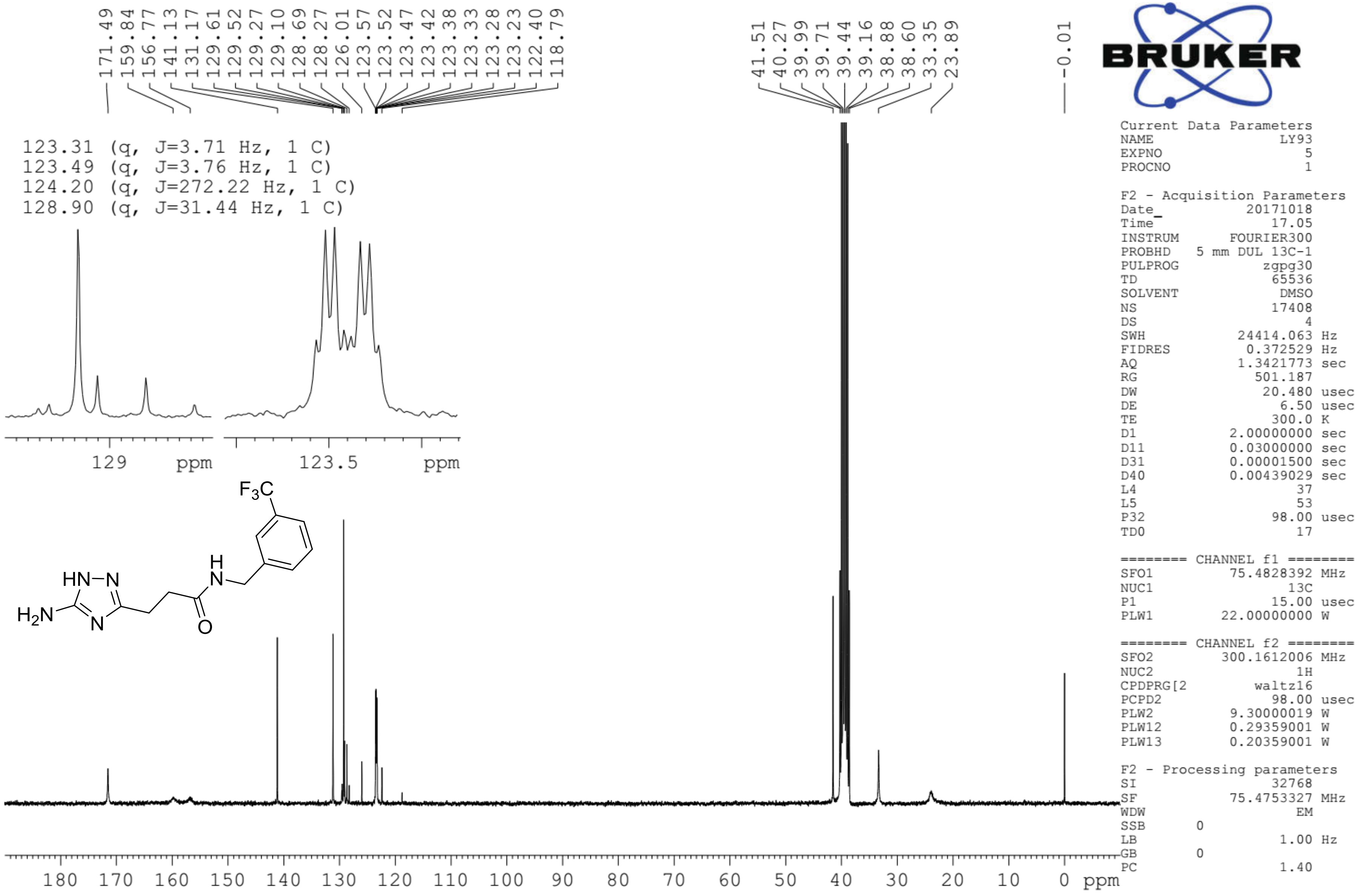

3-(5-Amino-1H-1,2,4-triazol-3-yl)-N-(phenylethyl)propanamide (5i)

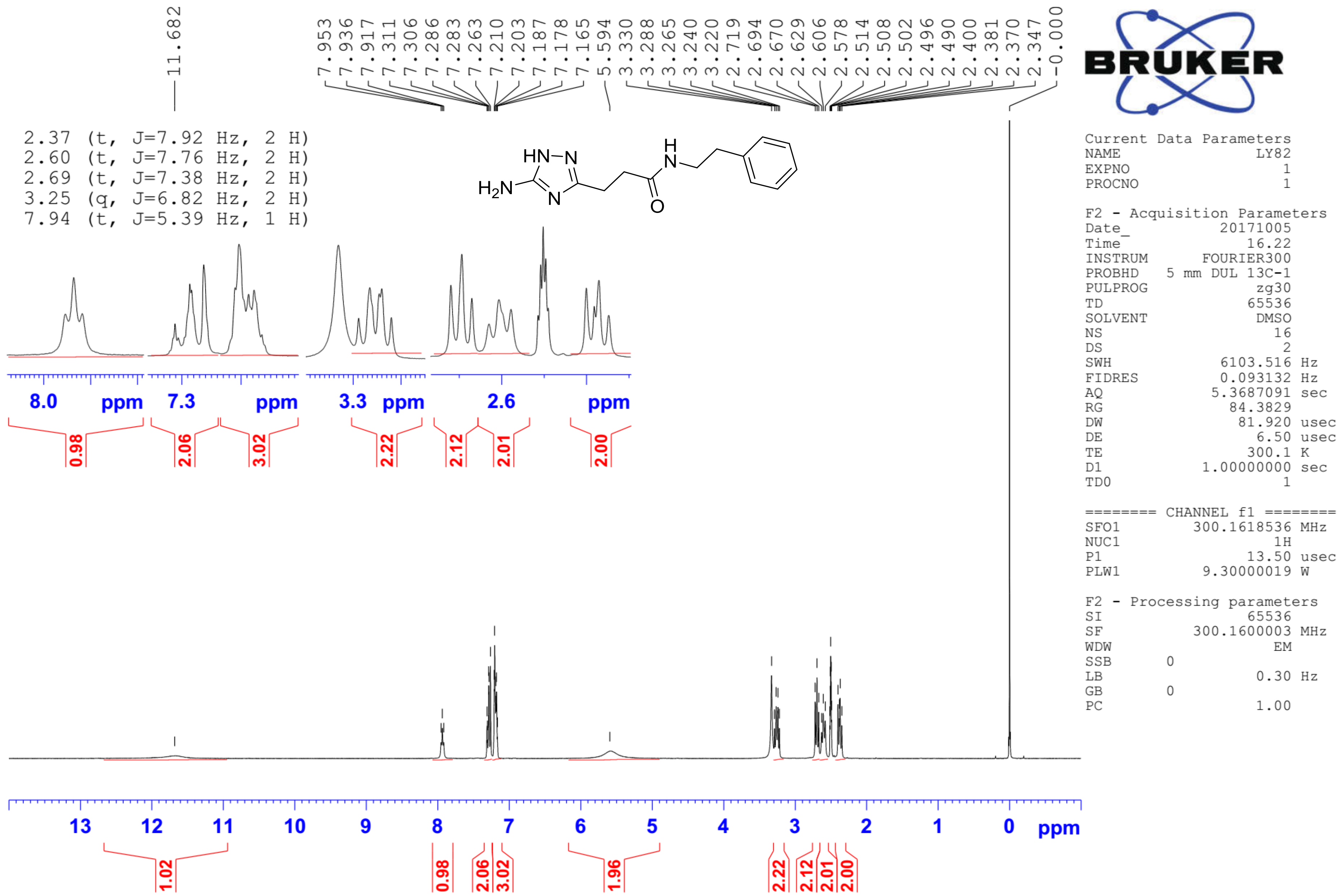

3-(5-Amino-1H-1,2,4-triazol-3-yl)-N-(phenylethyl)propanamide (5i)

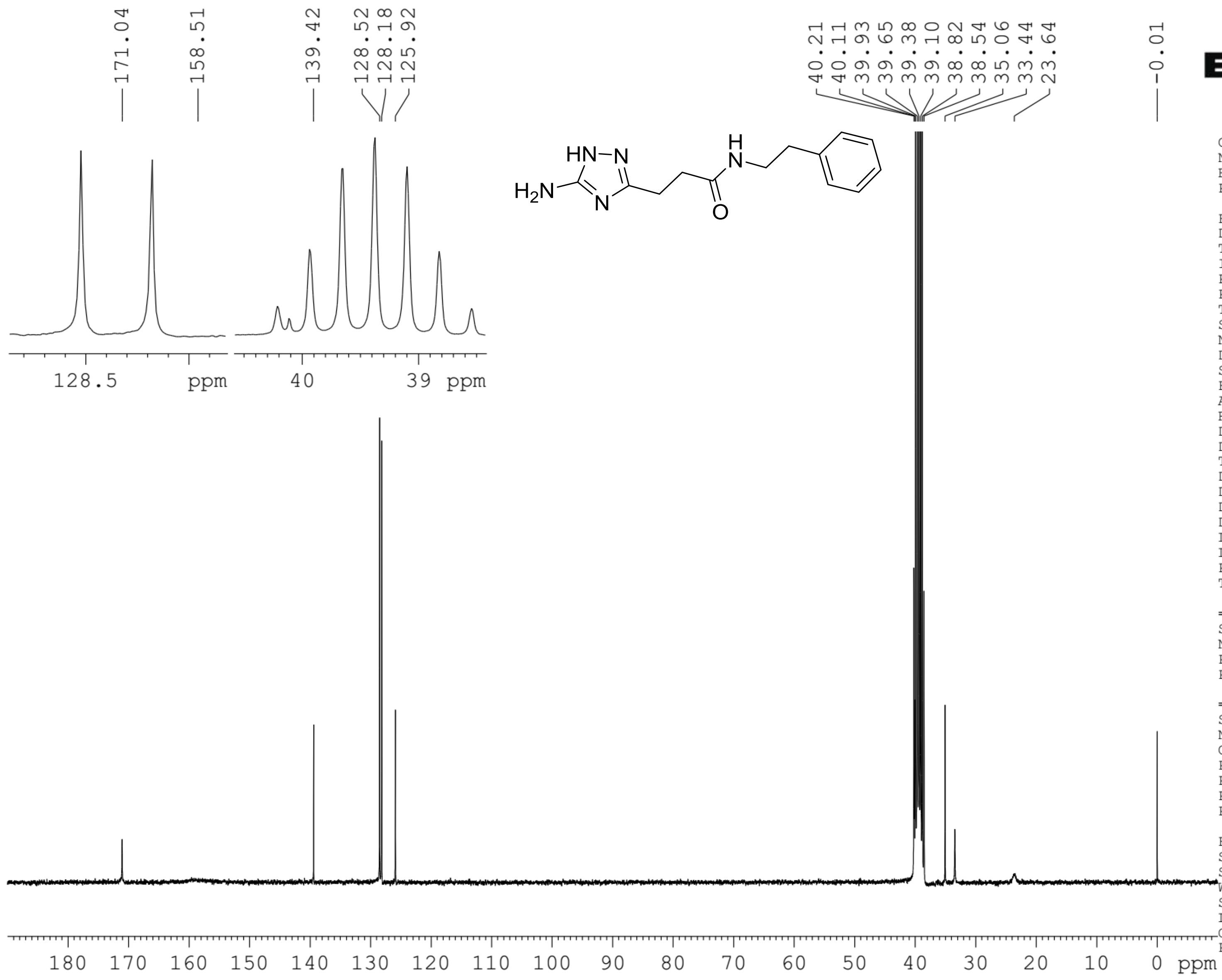

Current Data Parameters
NAME LY82
EXPNO 2
PROCNO 1

F2 - Acquisition Parameters
Date_ 20171007
Time 19.28
INSTRUM FOURIER300
PROBHD 5 mm DUL 13C-1
PULPROG zgpg30
TD 65536
SOLVENT DMSO
NS 15360
DS 4
SWH 24414.063 Hz
FIDRES 0.372529 Hz
AQ 1.3421773 sec
RG 501.187
DW 20.480 usec
DE 6.50 usec
TE 295.9 K
D1 2.00000000 sec
D11 0.03000000 sec
D31 0.00001500 sec
D40 0.00439029 sec
L4 37
L5 53
P32 98.00 usec
TD0 15

======== CHANNEL f1 ========
SFO1 75.4828392 MHz
NUC1 13C
P1 15.00 usec
PLW1 22.00000000 W

======== CHANNEL f2 ========
SFO2 300.1612006 MHz
NUC2 1H
CPDPRG[2 waltz16
PCPD2 98.00 usec
PLW2 9.30000019 W
PLW12 0.29359001 W
PLW13 0.20359001 W

F2 - Processing parameters
SI 32768
SF 75.4753357 MHz
WDW EM
SSB 0
LB 1.00 Hz
GB 0
PC 1.40

3-(5-Amino-1H-1,2,4-triazol-3-yl)-N-(phenyl)propanamide (5j)

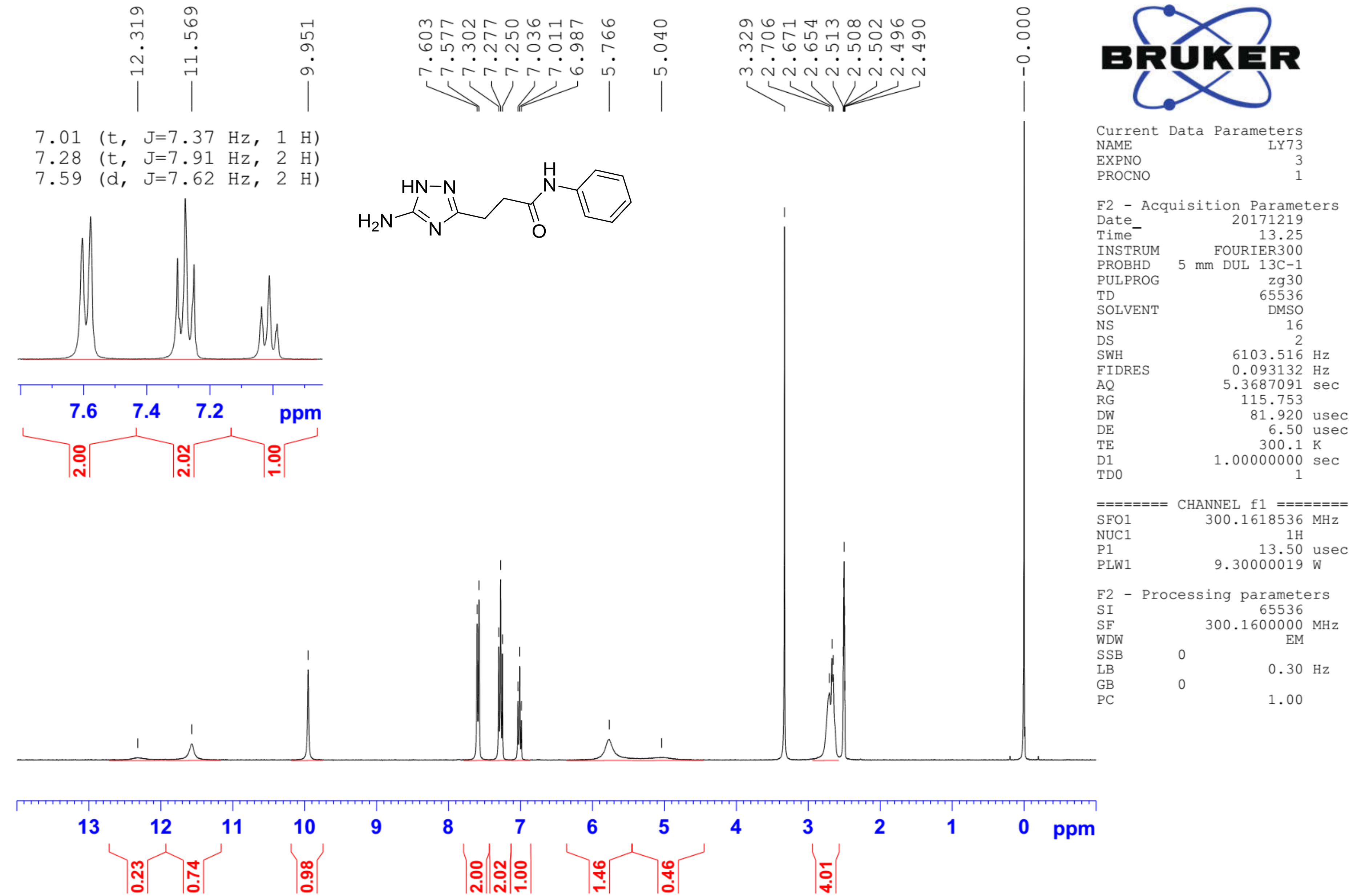

3-(5-Amino-1H-1,2,4-triazol-3-yl)-N-(phenyl)propanamide (5j)

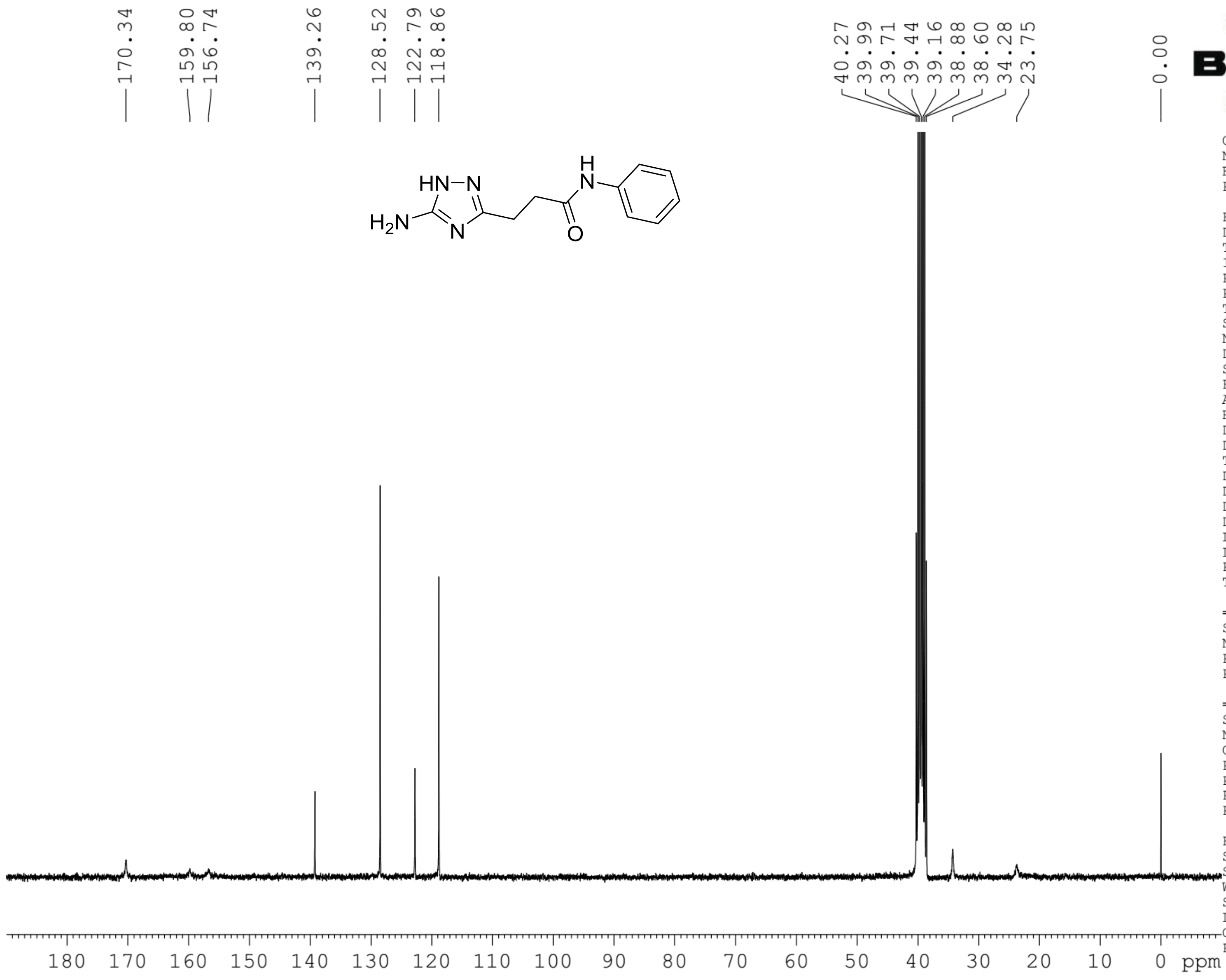


```
Current Data Parameters
NAME                LY73
EXPNO                  4
PROCNO                 1

F2 - Acquisition Parameters
Date_           20171219
Time               17.05
INSTRUM       FOURIER300
PROBHD   5 mm DUL 13C-1
PULPROG          zgpg30
TD                 65536
SOLVENT             DMSO
NS                 14336
DS                     4
SWH           24414.063 Hz
FIDRES         0.372529 Hz
AQ            1.3421773 sec
RG              501.187
DW               20.480 usec
DE                 6.50 usec
TE                300.0 K
D1           2.00000000 sec
D11          0.03000000 sec
D31          0.00001500 sec
D40          0.00439029 sec
L4                    37
L5                    53
P32                98.00 usec
TD0                   14

======== CHANNEL f1 ========
SFO1         75.4828392 MHz
NUC1                 13C
P1                 15.00 usec
PLW1         22.00000000 W

======== CHANNEL f2 ========
SFO2        300.1612006 MHz
NUC2                  1H
CPDPRG[2          waltz16
PCPD2              98.00 usec
PLW2          9.30000019 W
PLW12         0.29359001 W
PLW13         0.20359001 W

F2 - Processing parameters
SI                 32768
SF            75.4753349 MHz
WDW                   EM
SSB       0
LB                  1.00 Hz
GB        0
PC                  1.40
```

3-(5-Amino-1H-1,2,4-triazol-3-yl)-N-(phenyl)propanamide (5j)

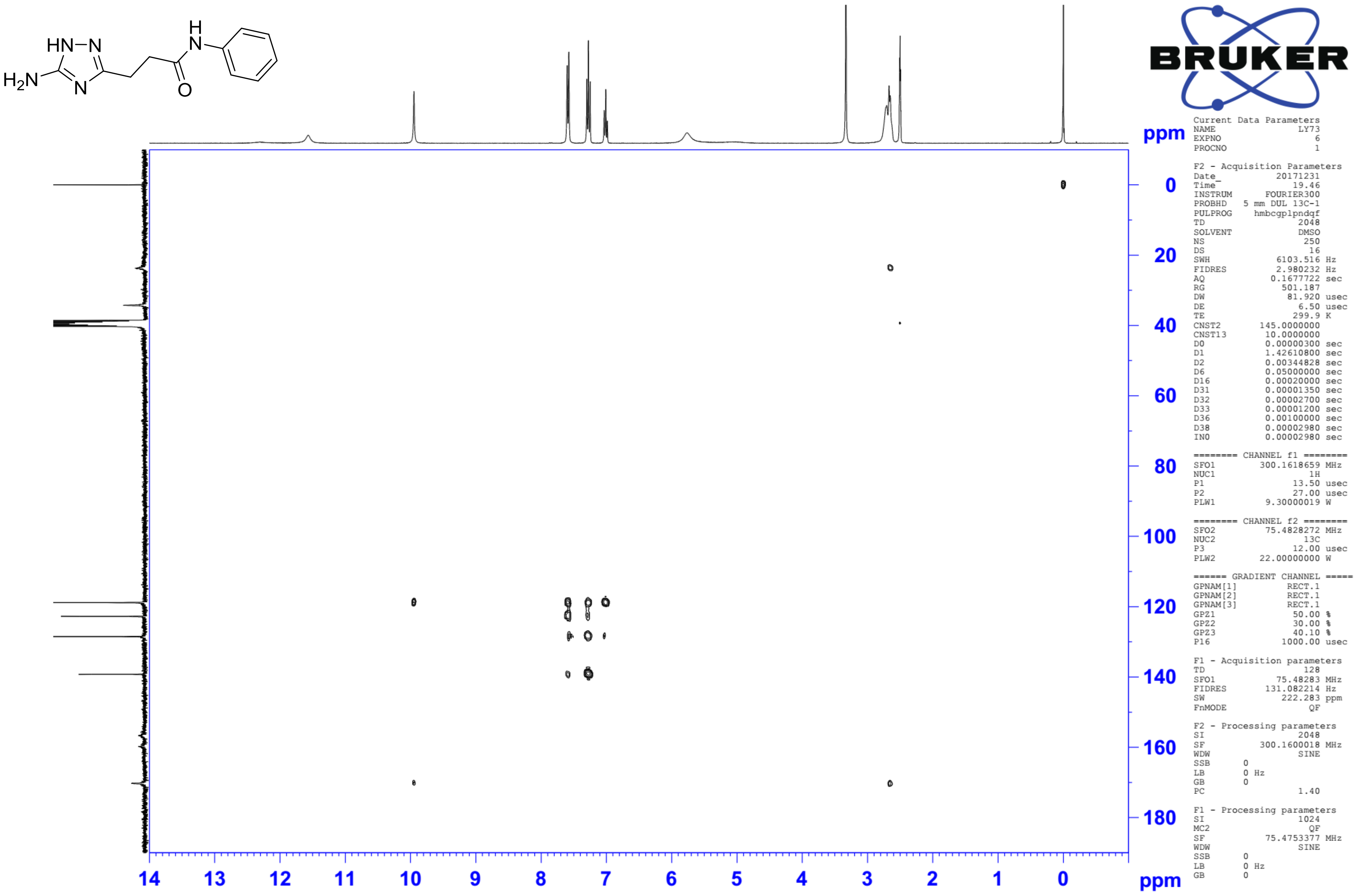

3-(5-Amino-1H-1,2,4-triazol-3-yl)-N-(4-fluorophenyl)propanamide (5k)

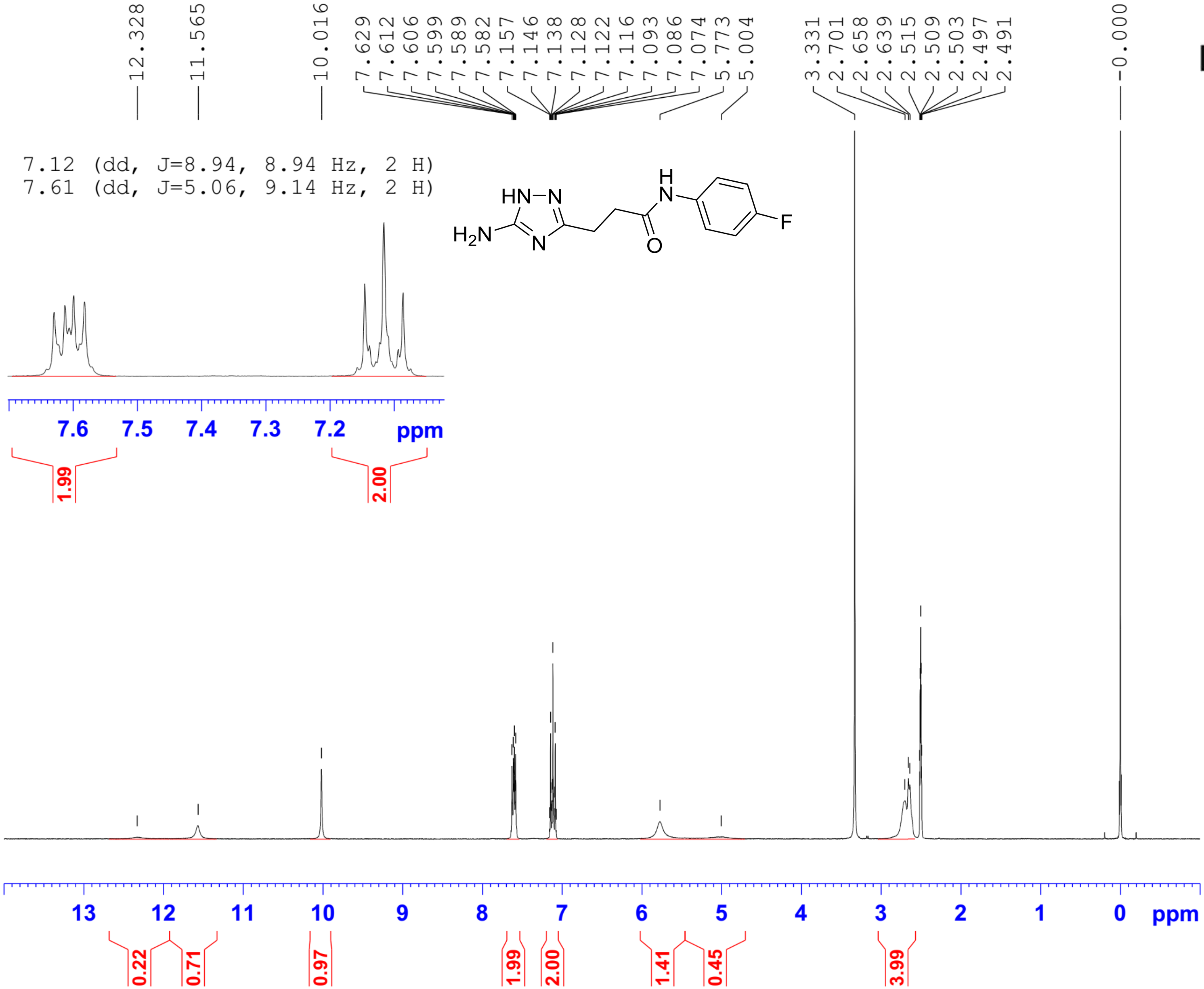

Current Data Parameters	
NAME	LY110
EXPNO	4
PROCNO	1
F2 - Acquisition Parameters	
Date_	20171229
Time	12.36
INSTRUM	FOURIER300
PROBHD	5 mm DUL 13C-1
PULPROG	zg30
TD	65536
SOLVENT	DMSO
NS	16
DS	2
SWH	6103.516 Hz
FIDRES	0.093132 Hz
AQ	5.3687091 sec
RG	117.114
DW	81.920 usec
DE	6.50 usec
TE	300.0 K
D1	1.00000000 sec
TD0	1
======== CHANNEL f1 ========	
SFO1	300.1618536 MHz
NUC1	1H
P1	13.50 usec
PLW1	9.30000019 W
F2 - Processing parameters	
SI	65536
SF	300.1599999 MHz
WDW	EM
SSB	0
LB	0.30 Hz
GB	0
PC	1.00

3-(5-Amino-1H-1,2,4-triazol-3-yl)-N-(4-fluorophenyl)propanamide (5k)

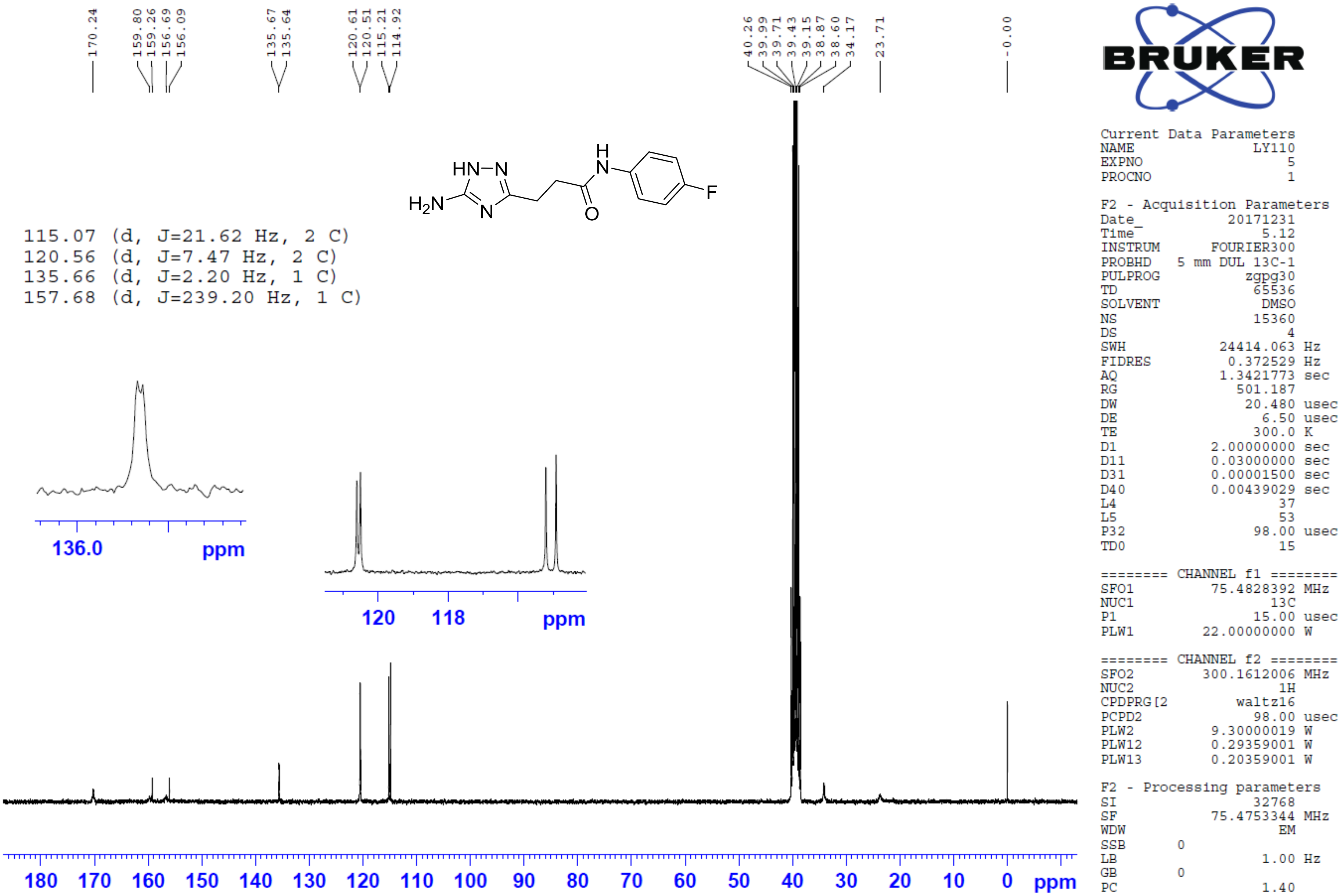

3-(5-Amino-1H-1,2,4-triazol-3-yl)-N-(2-chlorophenyl)propanamide (5l)

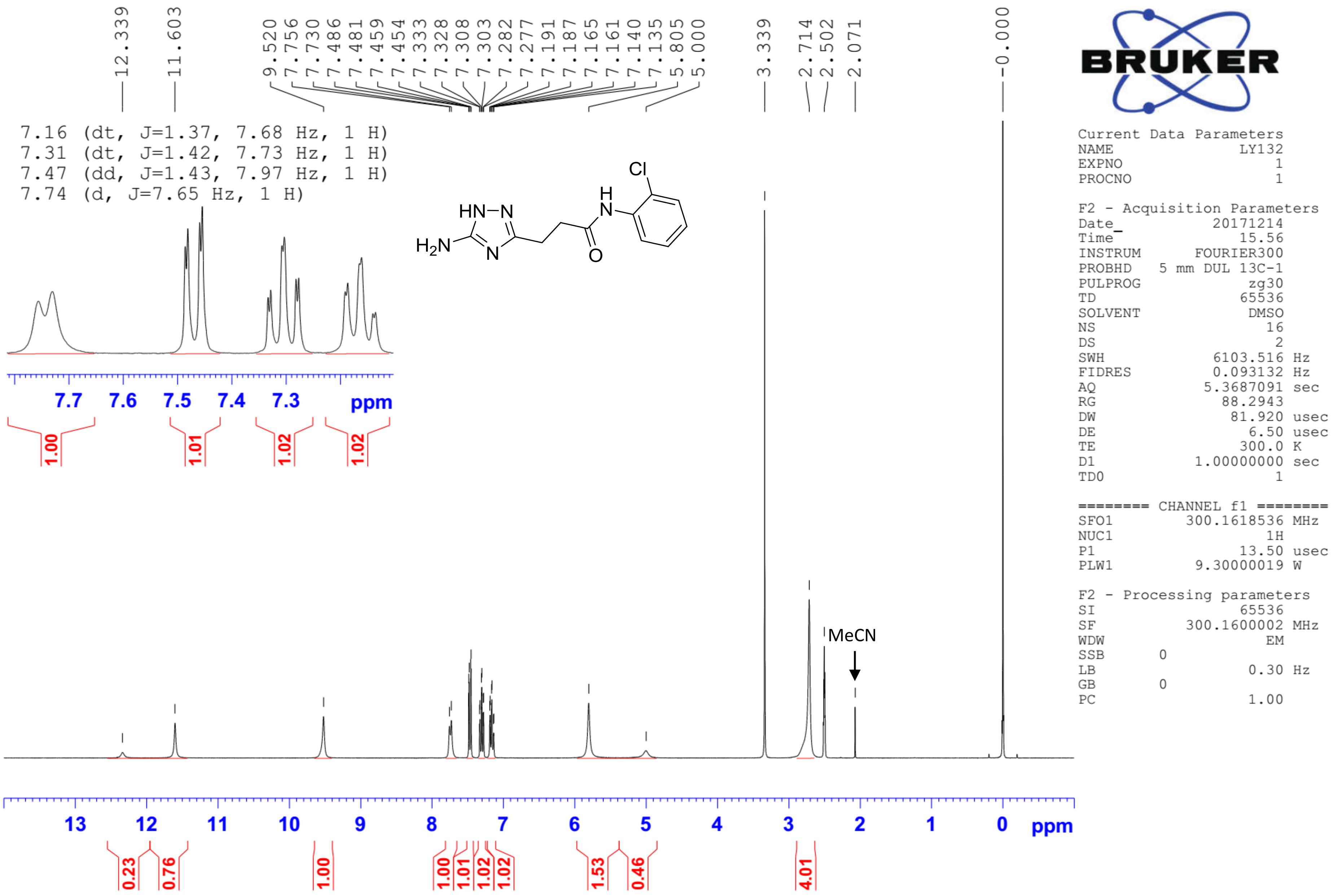

3-(5-Amino-1H-1,2,4-triazol-3-yl)-N-(2-chlorophenyl)propanamide (5l)

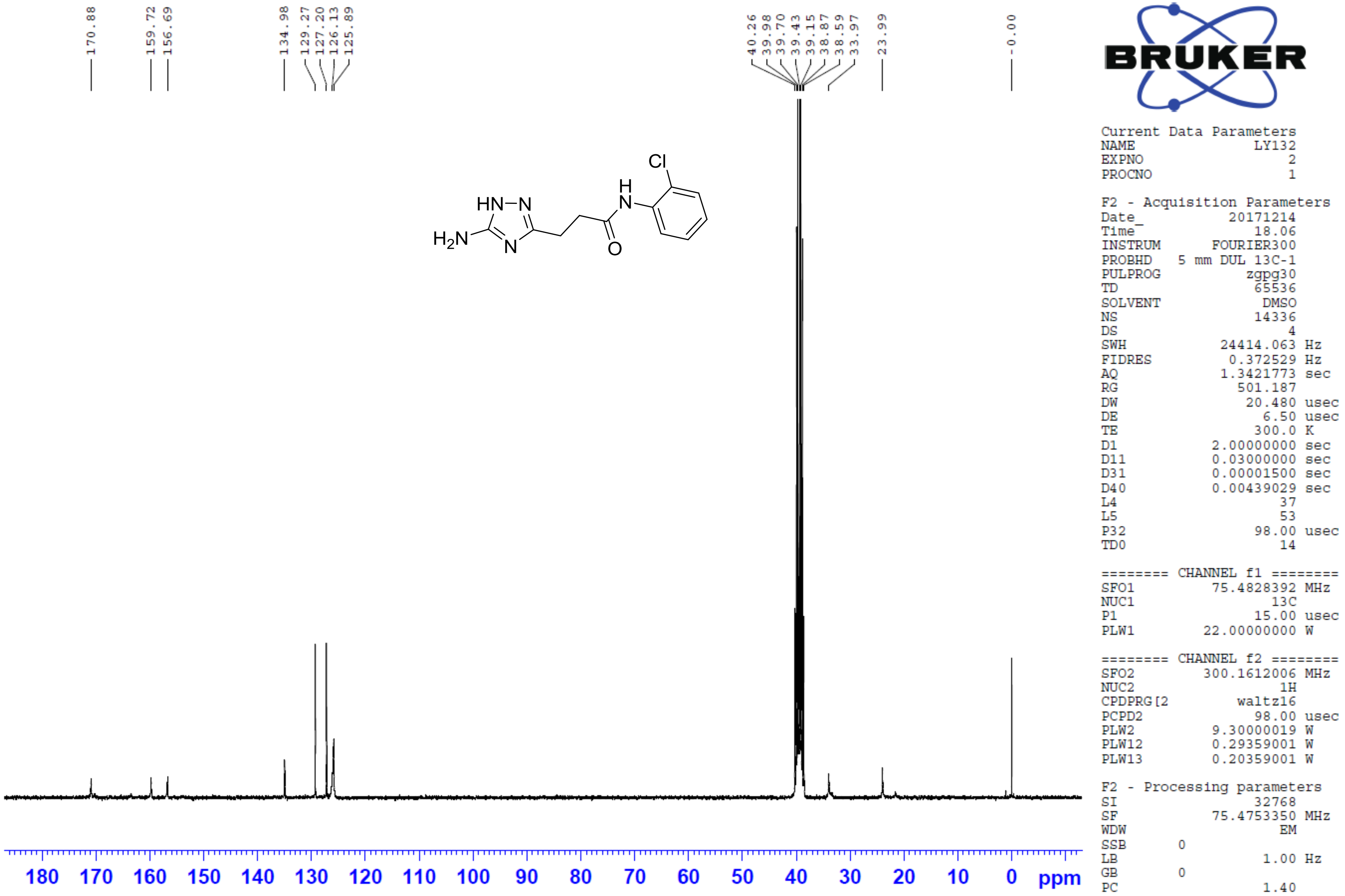

3-(5-Amino-1H-1,2,4-triazol-3-yl)-N-(3-chlorophenyl)propanamide (5m)

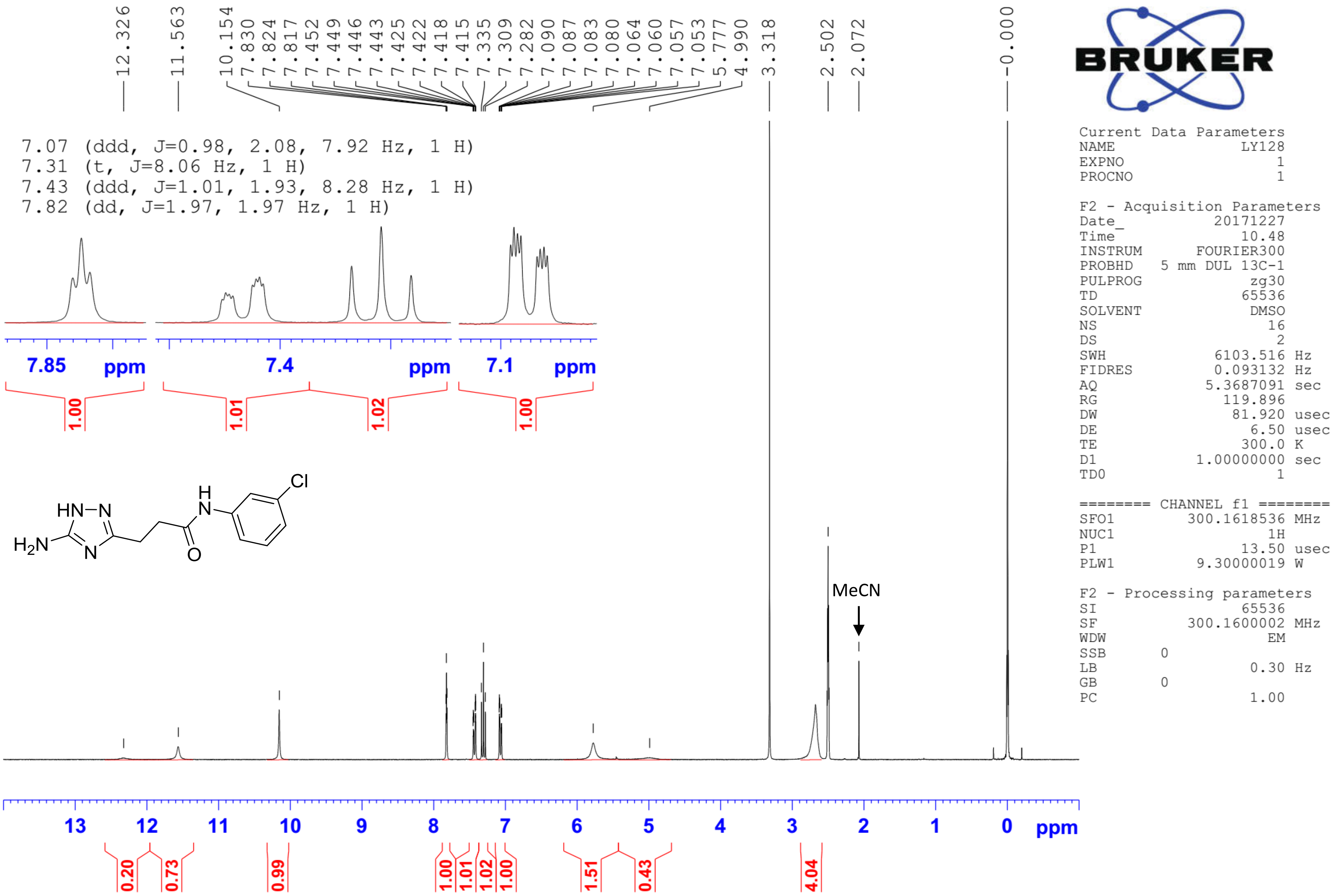

3-(5-Amino-1H-1,2,4-triazol-3-yl)-N-(3-chlorophenyl)propanamide (5m)

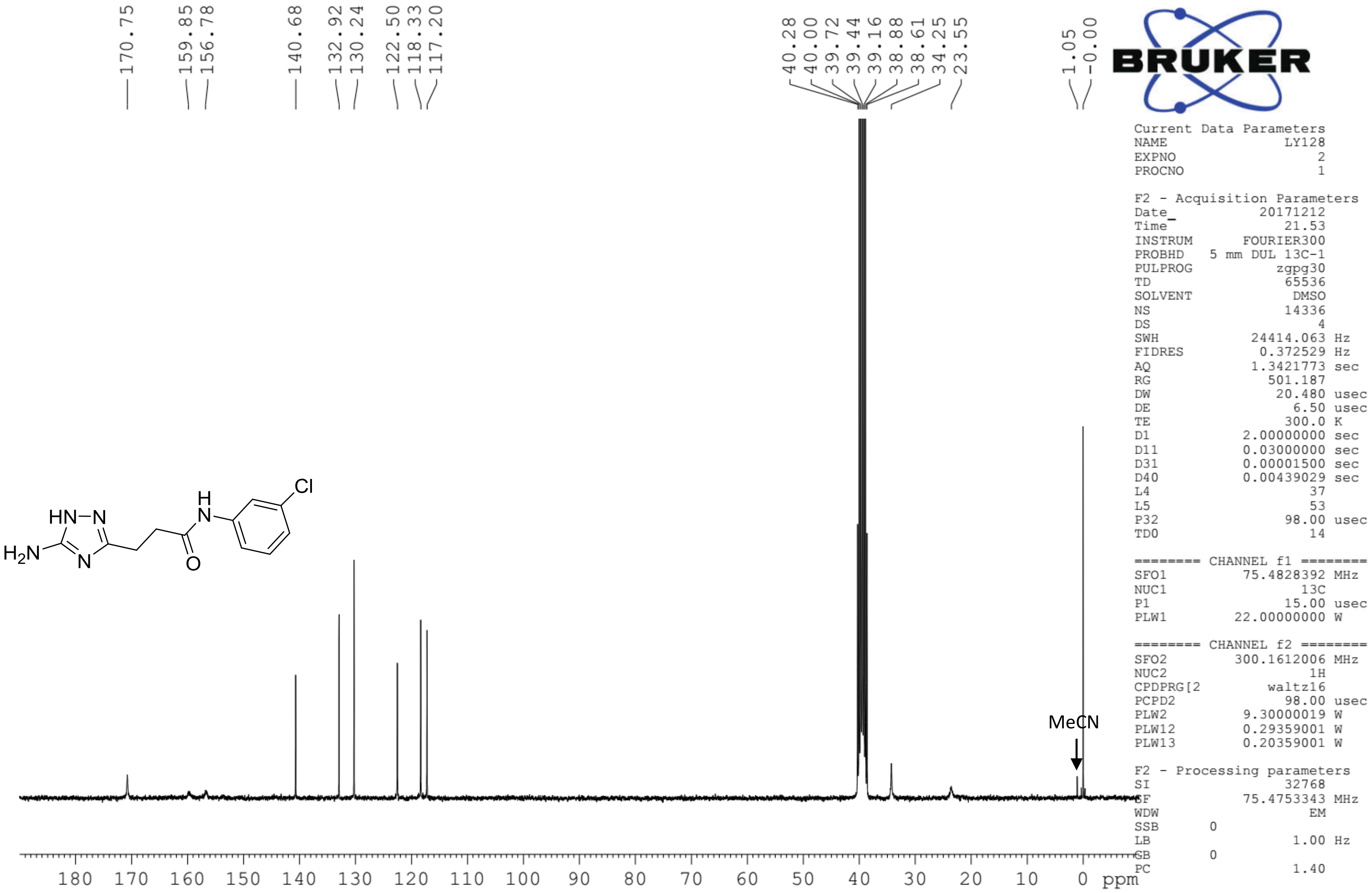

3-(5-Amino-1H-1,2,4-triazol-3-yl)-N-(4-chlorophenyl)propanamide (5n)

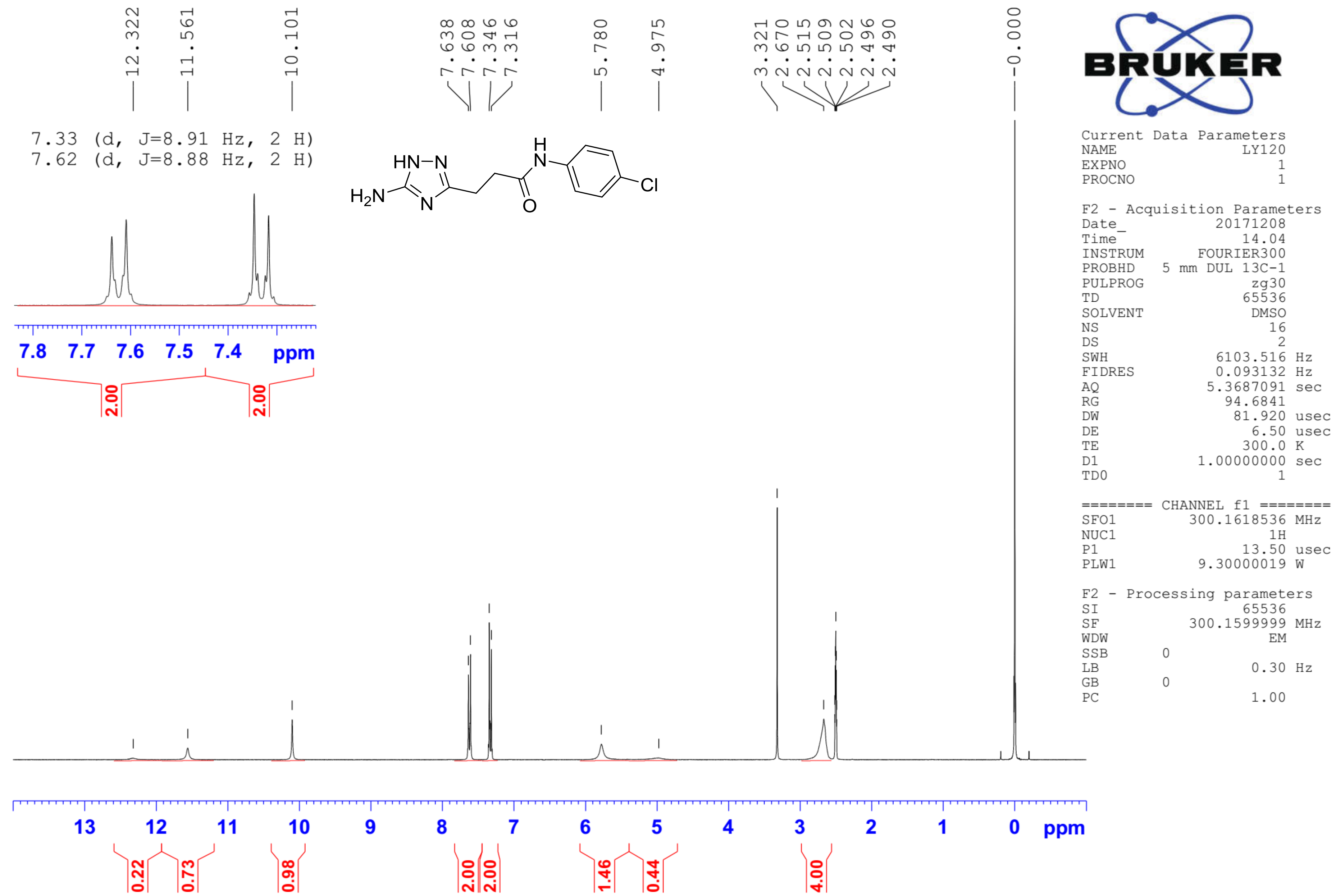

3-(5-Amino-1H-1,2,4-triazol-3-yl)-N-(4-chlorophenyl)propanamide (5n)

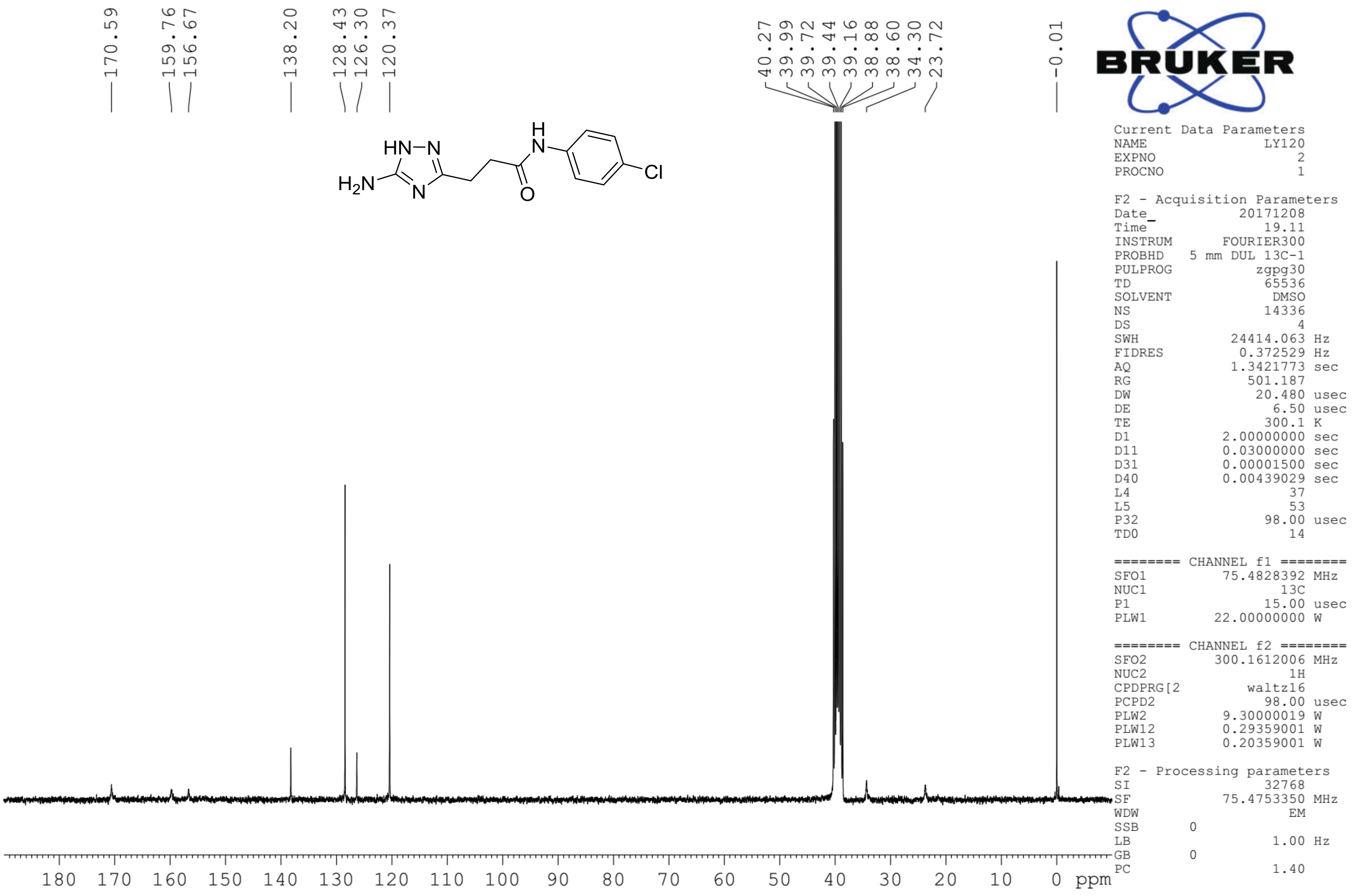

3-(5-Amino-1H-1,2,4-triazol-3-yl)-N-(3-methylphenyl)propanamide (5o)

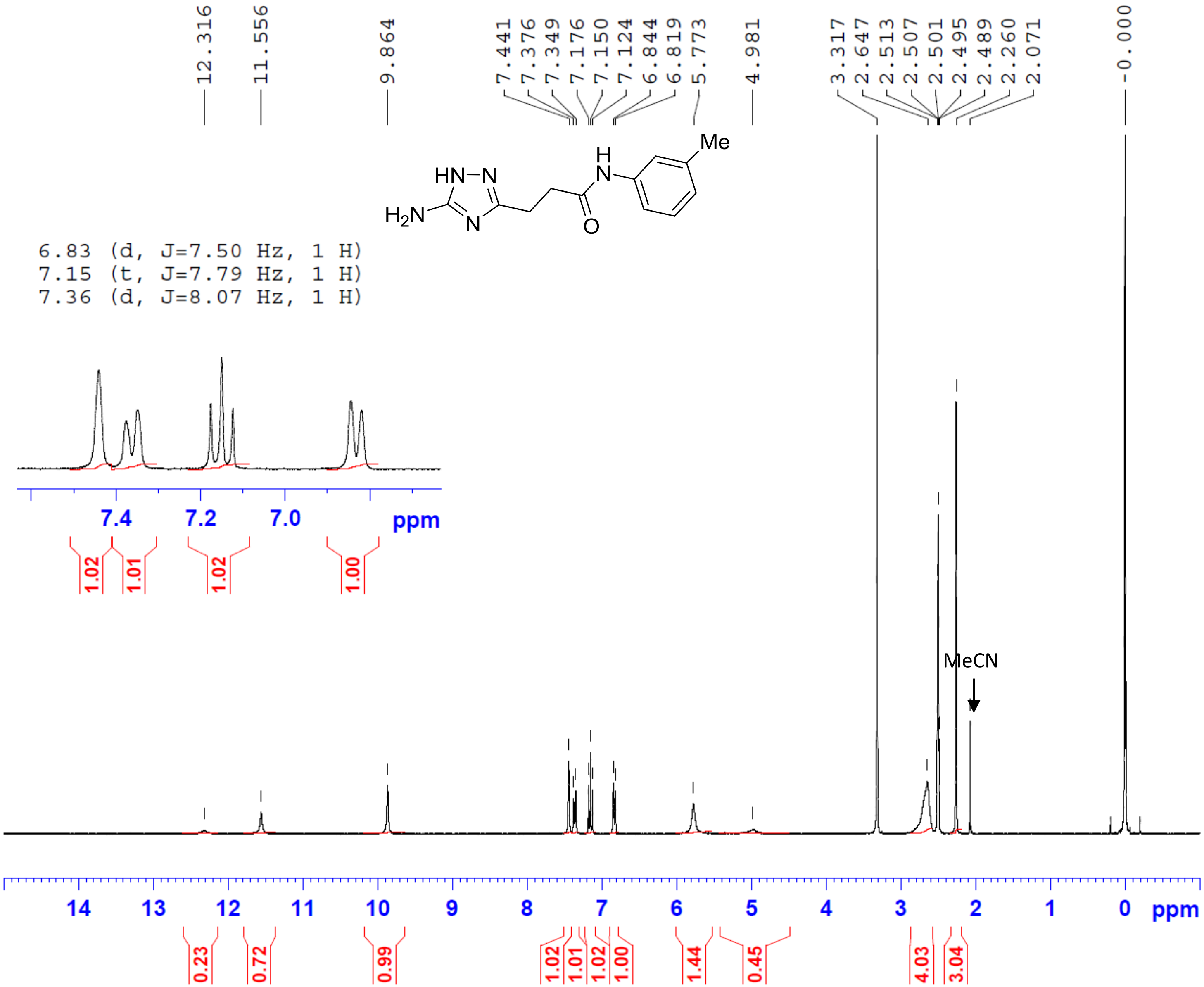

Current Data Parameters
NAME LY123
EXPNO 1
PROCNO 1

F2 - Acquisition Parameters
Date_ 20171228
Time 16.24
INSTRUM FOURIER300
PROBHD 5 mm DUL 13C-1
PULPROG zg30
TD 65536
SOLVENT DMSO
NS 16
DS 2
SWH 6103.516 Hz
FIDRES 0.093132 Hz
AQ 5.3687091 sec
RG 116.552
DW 81.920 usec
DE 6.50 usec
TE 300.0 K
D1 1.00000000 sec
TD0 1

======== CHANNEL f1 ========
SFO1 300.1618536 MHz
NUC1 1H
P1 13.50 usec
PLW1 9.30000019 W

F2 - Processing parameters
SI 65536
SF 300.1600003 MHz
WDW EM
SSB 0
LB 0.30 Hz
GB 0
PC 1.00

3-(5-Amino-1H-1,2,4-triazol-3-yl)-N-(3-methylphenyl)propanamide (5o)

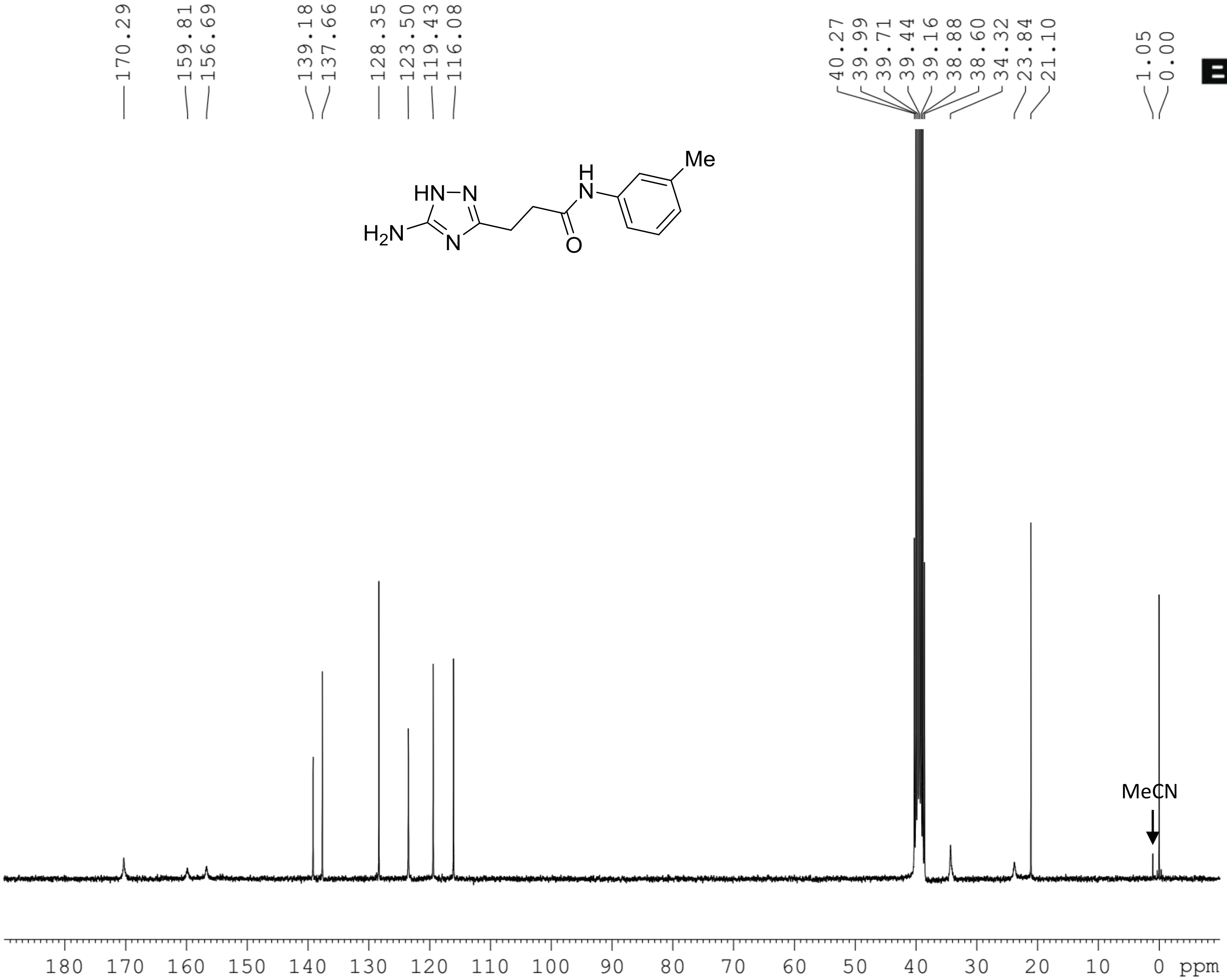


```
Current Data Parameters
NAME                 LY123
EXPNO                    2
PROCNO                   1

F2 - Acquisition Parameters
Date_             20171211
Time                 19.43
INSTRUM         FOURIER300
PROBHD   5 mm DUL 13C-1
PULPROG            zgpg30
TD                  65536
SOLVENT              DMSO
NS                  14336
DS                      4
SWH             24414.063 Hz
FIDRES           0.372529 Hz
AQ              1.3421773 sec
RG                501.187
DW                 20.480 usec
DE                   6.50 usec
TE                  300.1 K
D1             2.00000000 sec
D11            0.03000000 sec
D31            0.00001500 sec
D40            0.00439029 sec
L4                     37
L5                     53
P32                 98.00 usec
TD0                    14

======== CHANNEL f1 ========
SFO1           75.4828392 MHz
NUC1                  13C
P1                  15.00 usec
PLW1          22.00000000 W

======== CHANNEL f2 ========
SFO2          300.1612006 MHz
NUC2                   1H
CPDPRG[2          waltz16
PCPD2               98.00 usec
PLW2           9.30000019 W
PLW12          0.29359001 W
PLW13          0.20359001 W

F2 - Processing parameters
SI                  32768
SF             75.4753344 MHz
WDW                    EM
SSB        0
LB                   1.00 Hz
GB         0
PC                   1.40
```

3-(5-Amino-1H-1,2,4-triazol-3-yl)-N-(4-methylphenyl)propanamide (5p)

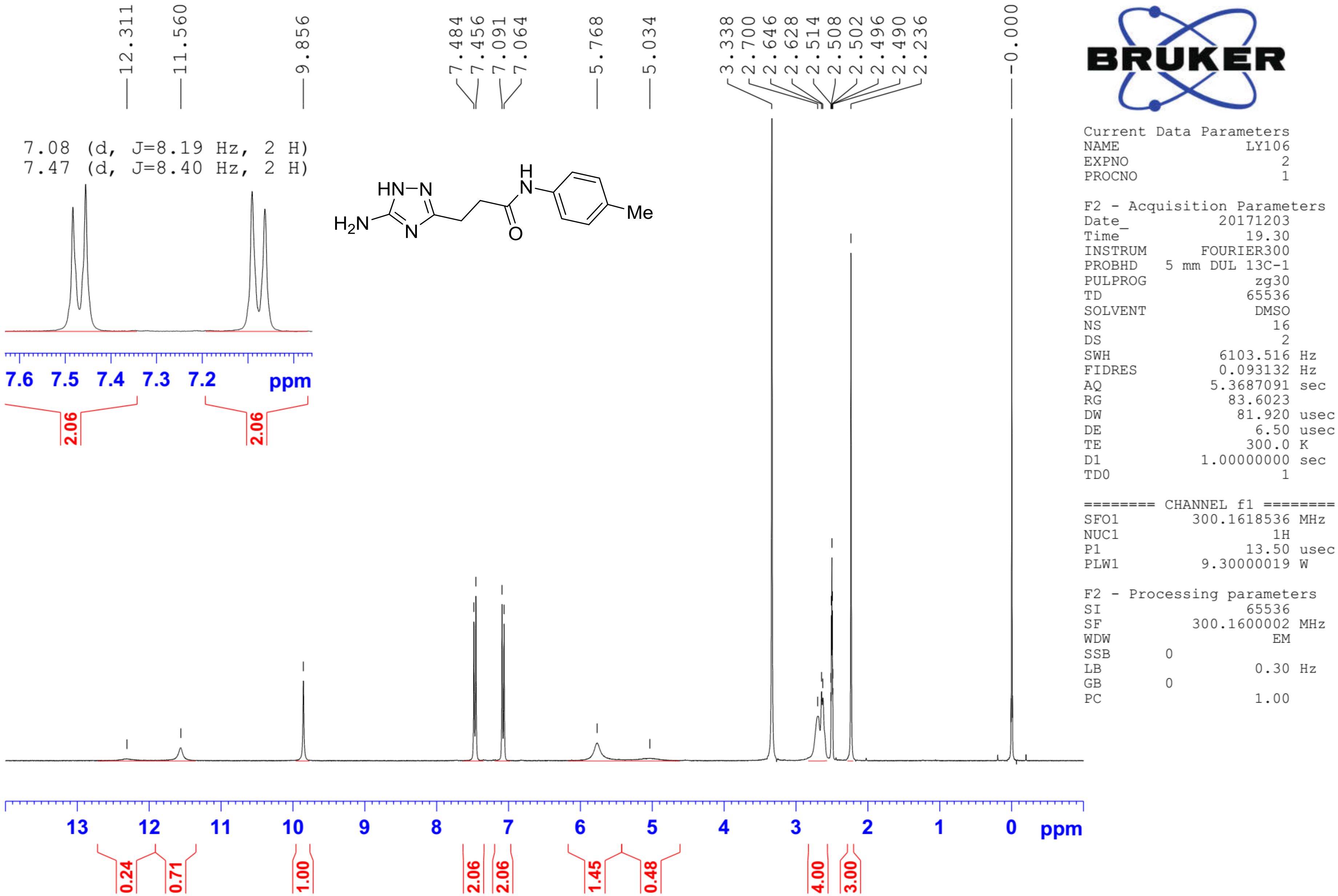

3-(5-Amino-1H-1,2,4-triazol-3-yl)-N-(4-methylphenyl)propanamide (5p)

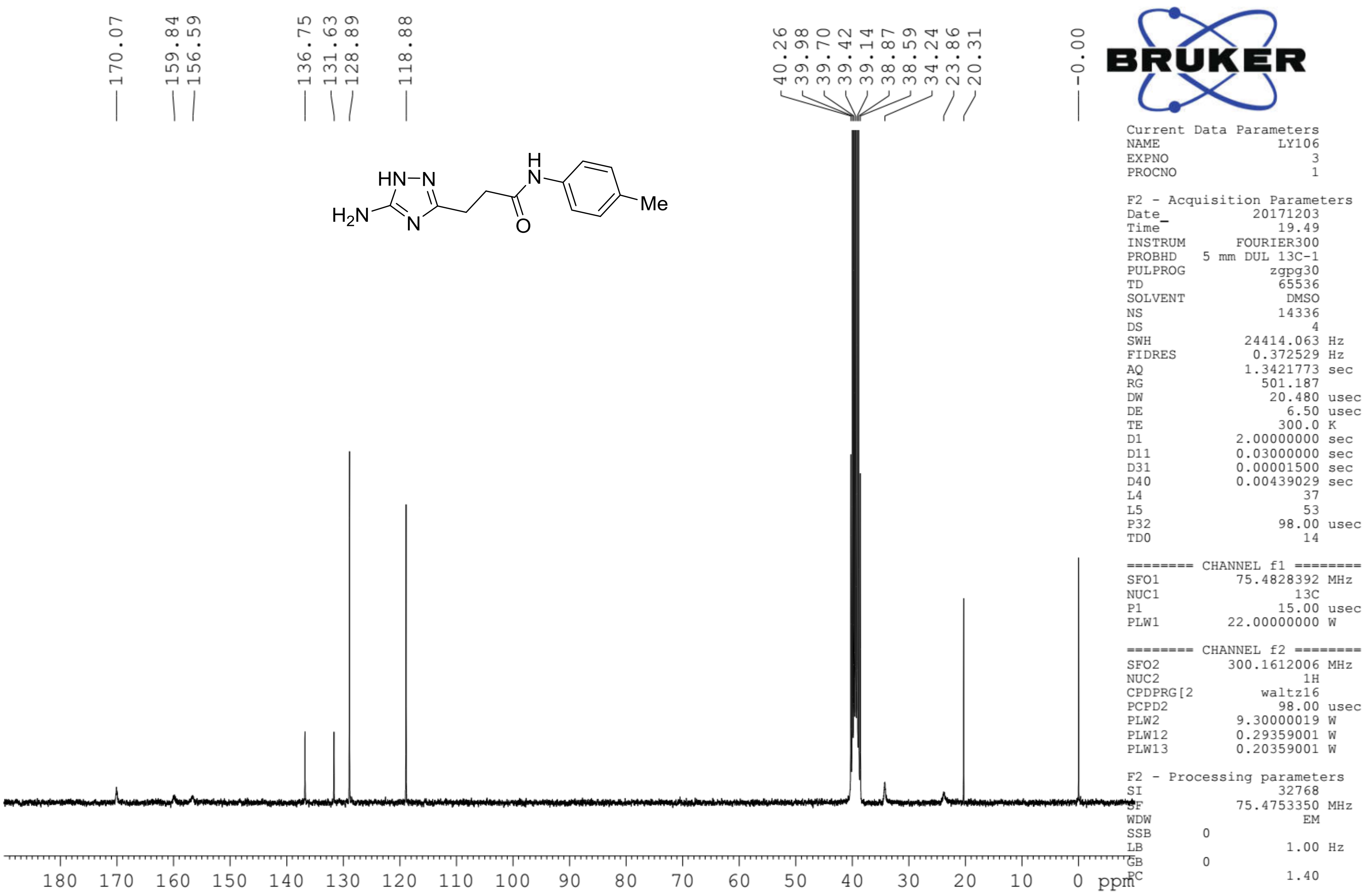

3-(5-Amino-1H-1,2,4-triazol-3-yl)-N-(4-isopropylphenyl)propanamide (5q)

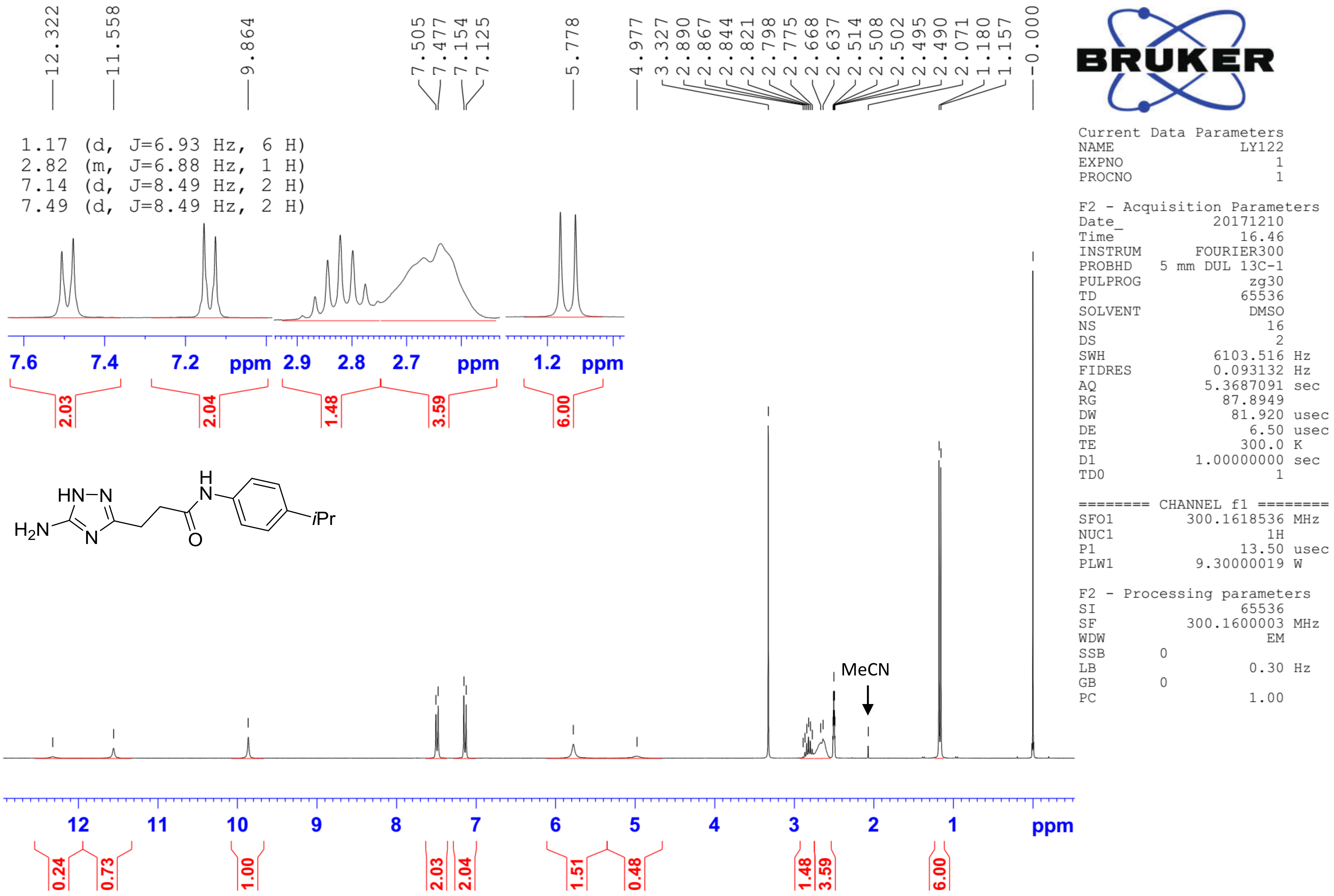

3-(5-Amino-1H-1,2,4-triazol-3-yl)-N-(4-isopropylphenyl)propanamide (5q)

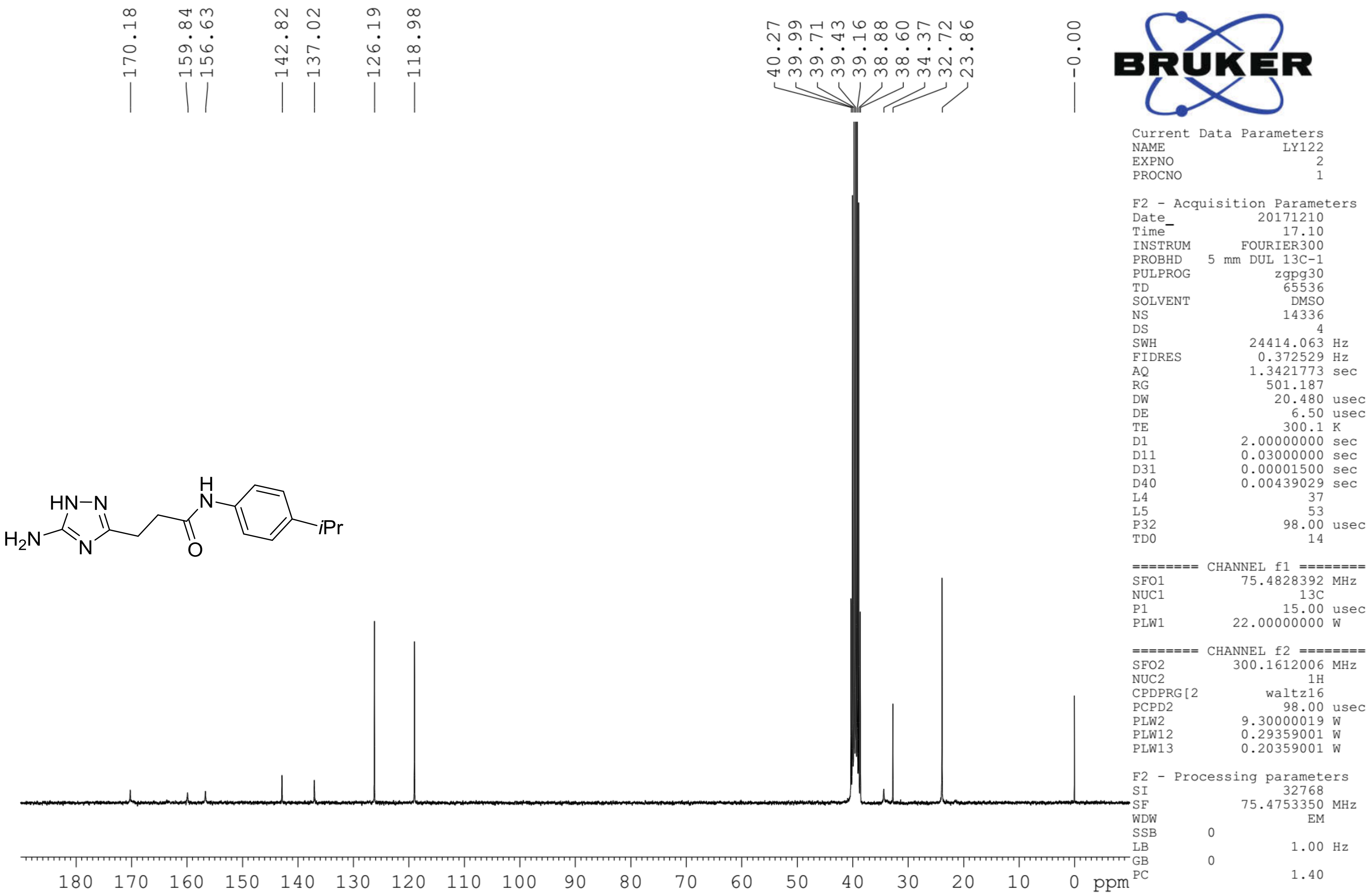

3-(5-Amino-1H-1,2,4-triazol-3-yl)-N-(3-methoxyphenyl)propanamide (5r)

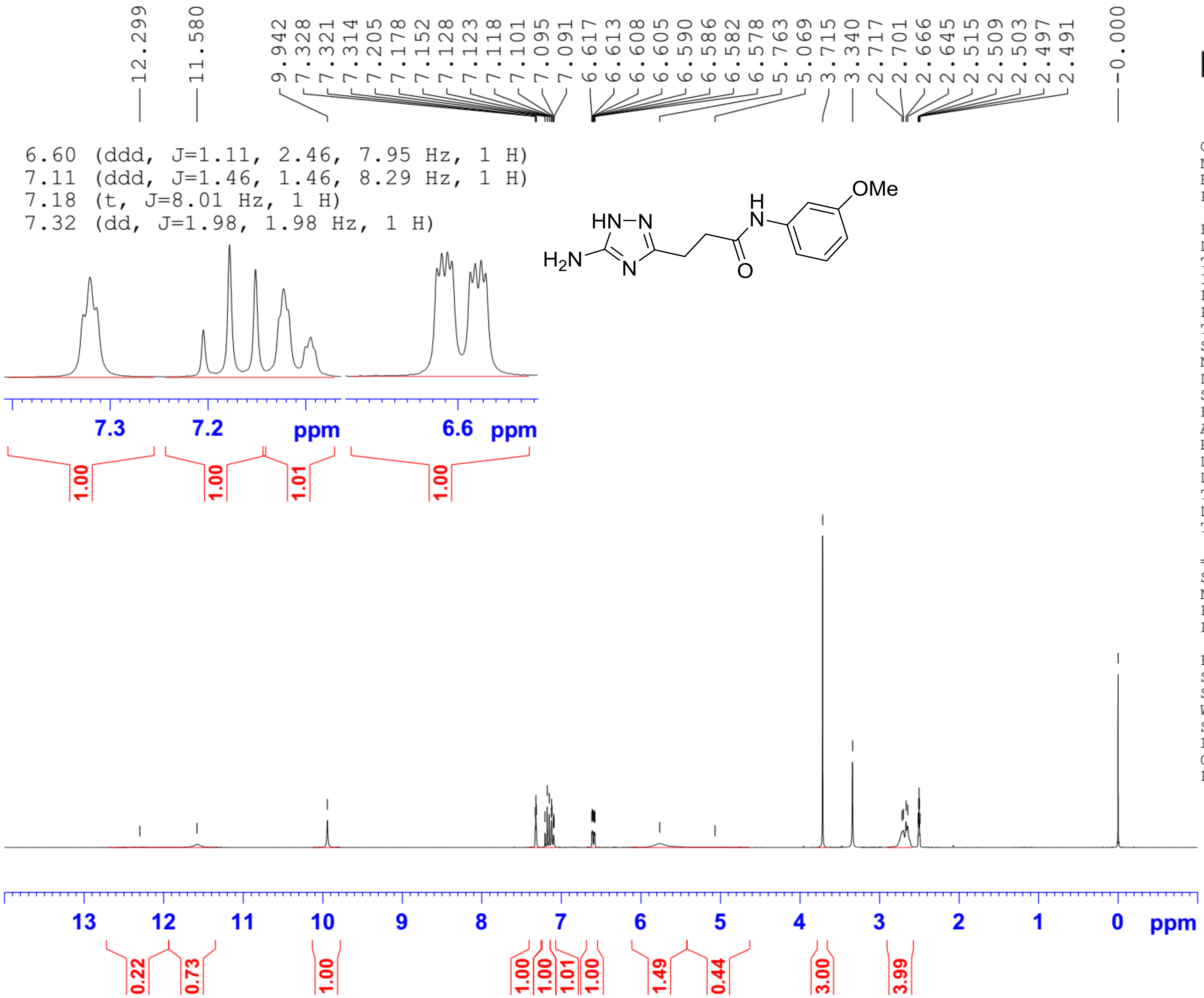

Current Data Parameters
NAME LY140
EXPNO 1
PROCNO 1

F2 - Acquisition Parameters
Date_ 20171219
Time 13.29
INSTRUM FOURIER300
PROBHD 5 mm DUL 13C-1
PULPROG zg30
TD 65536
SOLVENT DMSO
NS 16
DS 2
SWH 6103.702 Hz
FIDRES 0.093135 Hz
AQ 5.3685451 sec
RG 141.254
DW 81.918 usec
DE 6.50 usec
TE 300.0 K
D1 1.00000000 sec
TD0 1

======== CHANNEL f1 ========
SFO1 300.1618536 MHz
NUC1 1H
P1 13.50 usec
PLW1 9.30000019 W

F2 - Processing parameters
SI 65536
SF 300.1599999 MHz
WDW EM
SSB 0
LB 0.30 Hz
GB 0
PC 1.00

3-(5-Amino-1H-1,2,4-triazol-3-yl)-N-(3-methoxyphenyl)propanamide (5r)

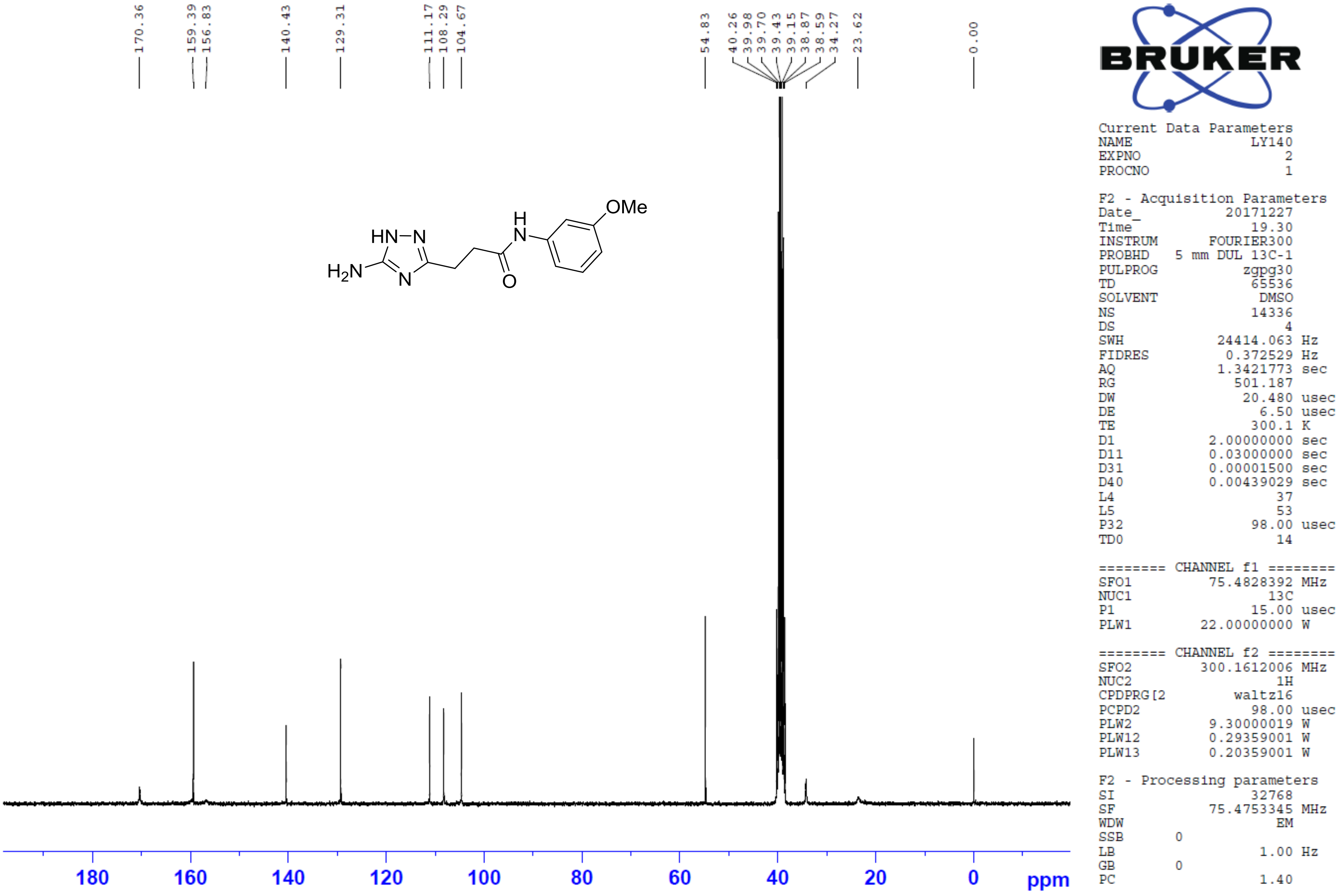

3-(5-Amino-1H-1,2,4-triazol-3-yl)-N-(4-methoxyphenyl)propanamide (5s)

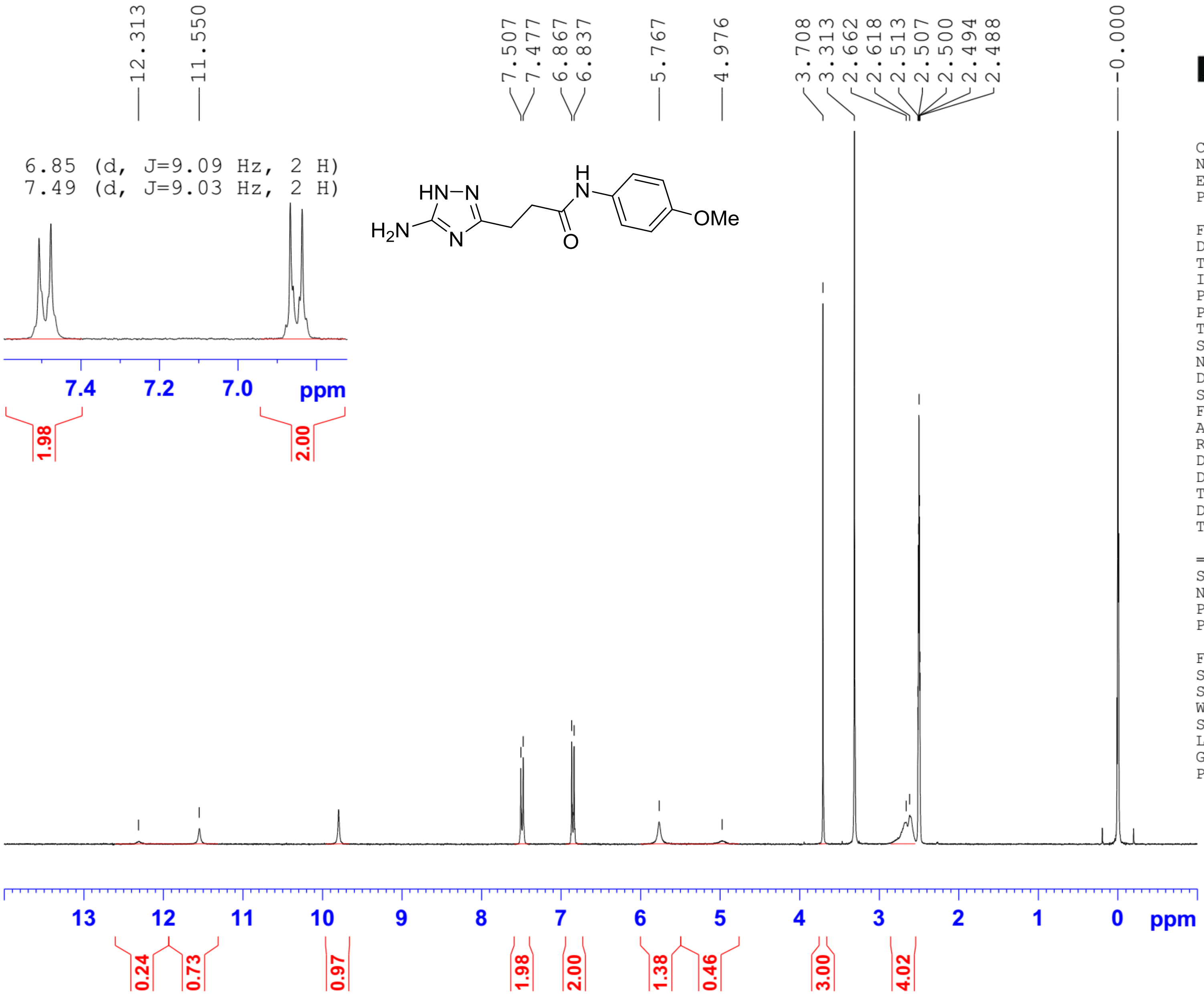

Current Data Parameters	
NAME	LY109
EXPNO	3
PROCNO	1
F2 - Acquisition Parameters	
Date_	20171226
Time	16.14
INSTRUM	FOURIER300
PROBHD	5 mm DUL 13C-1
PULPROG	zg30
TD	65536
SOLVENT	DMSO
NS	16
DS	2
SWH	6103.516 Hz
FIDRES	0.093132 Hz
AQ	5.3687091 sec
RG	158.089
DW	81.920 usec
DE	6.50 usec
TE	300.0 K
D1	1.00000000 sec
TD0	1
======== CHANNEL f1 ========	
SFO1	300.1618536 MHz
NUC1	1H
P1	13.50 usec
PLW1	9.30000019 W
F2 - Processing parameters	
SI	65536
SF	300.1600008 MHz
WDW	EM
SSB	0
LB	0.30 Hz
GB	0
PC	1.00

3-(5-Amino-1H-1,2,4-triazol-3-yl)-N-(4-methoxyphenyl)propanamide (5s)

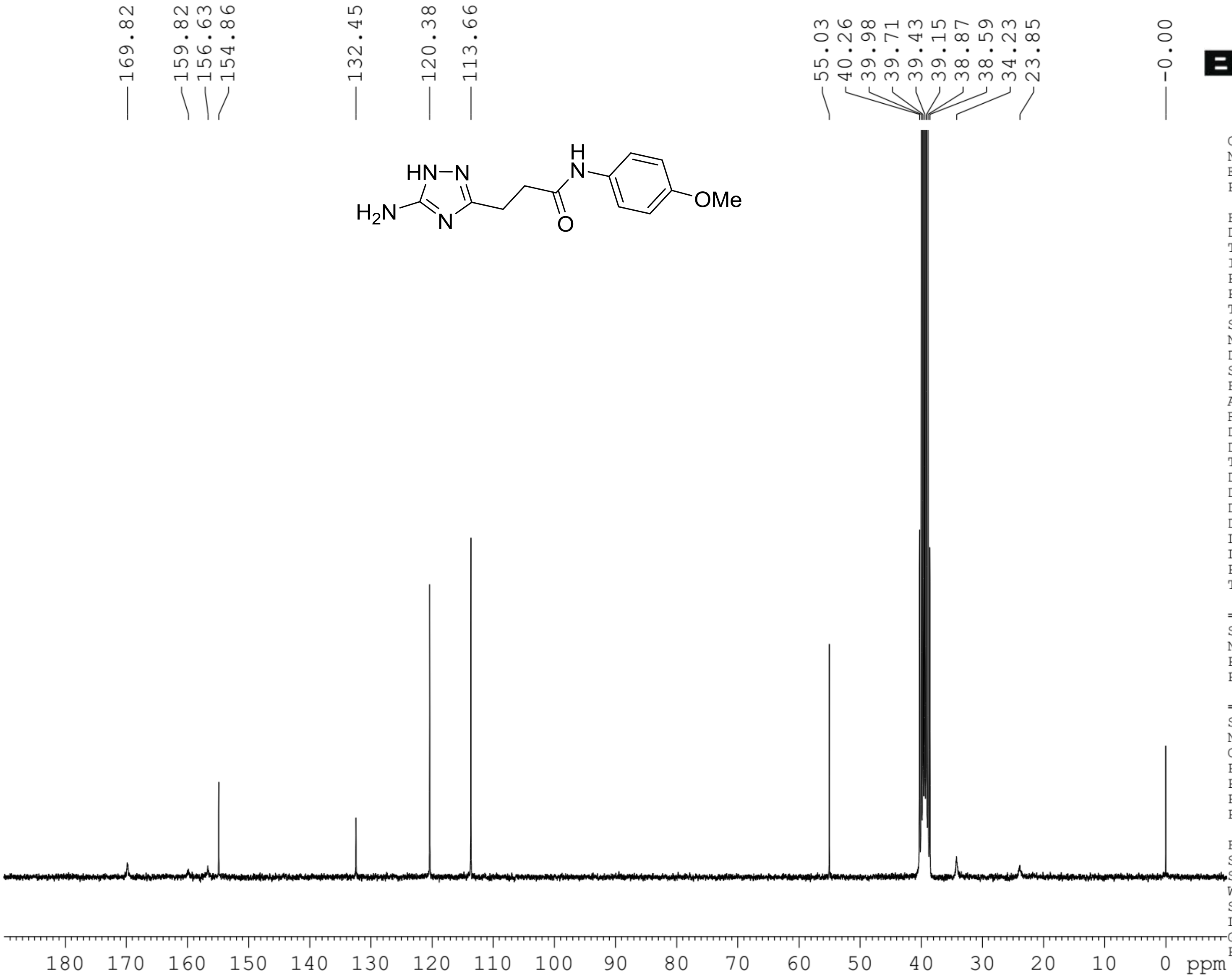


```
Current Data Parameters
NAME                    LY109
EXPNO                       2
PROCNO                      1

F2 - Acquisition Parameters
Date_                20171201
Time                    20.18
INSTRUM            FOURIER300
PROBHD     5 mm DUL 13C-1
PULPROG               zgpg30
TD                      65536
SOLVENT                  DMSO
NS                      14336
DS                          4
SWH               24414.063 Hz
FIDRES             0.372529 Hz
AQ                1.3421773 sec
RG                  501.187
DW                   20.480 usec
DE                     6.50 usec
TE                    300.0 K
D1               2.00000000 sec
D11              0.03000000 sec
D31              0.00001500 sec
D40              0.00439029 sec
L4                         37
L5                         53
P32                     98.00 usec
TD0                        14

======== CHANNEL f1 ========
SFO1             75.4828392 MHz
NUC1                      13C
P1                      15.00 usec
PLW1          22.00000000 W

======== CHANNEL f2 ========
SFO2            300.1612006 MHz
NUC2                       1H
CPDPRG[2              waltz16
PCPD2                   98.00 usec
PLW2             9.30000019 W
PLW12            0.29359001 W
PLW13            0.20359001 W

F2 - Processing parameters
SI                      32768
SF               75.4753350 MHz
WDW                        EM
SSB      0
LB                       1.00 Hz
GB       0
PC                       1.40
```

3-(5-Amino-1H-1,2,4-triazol-3-yl)-N-(4-acetamidophenyl)propanamide (5t)

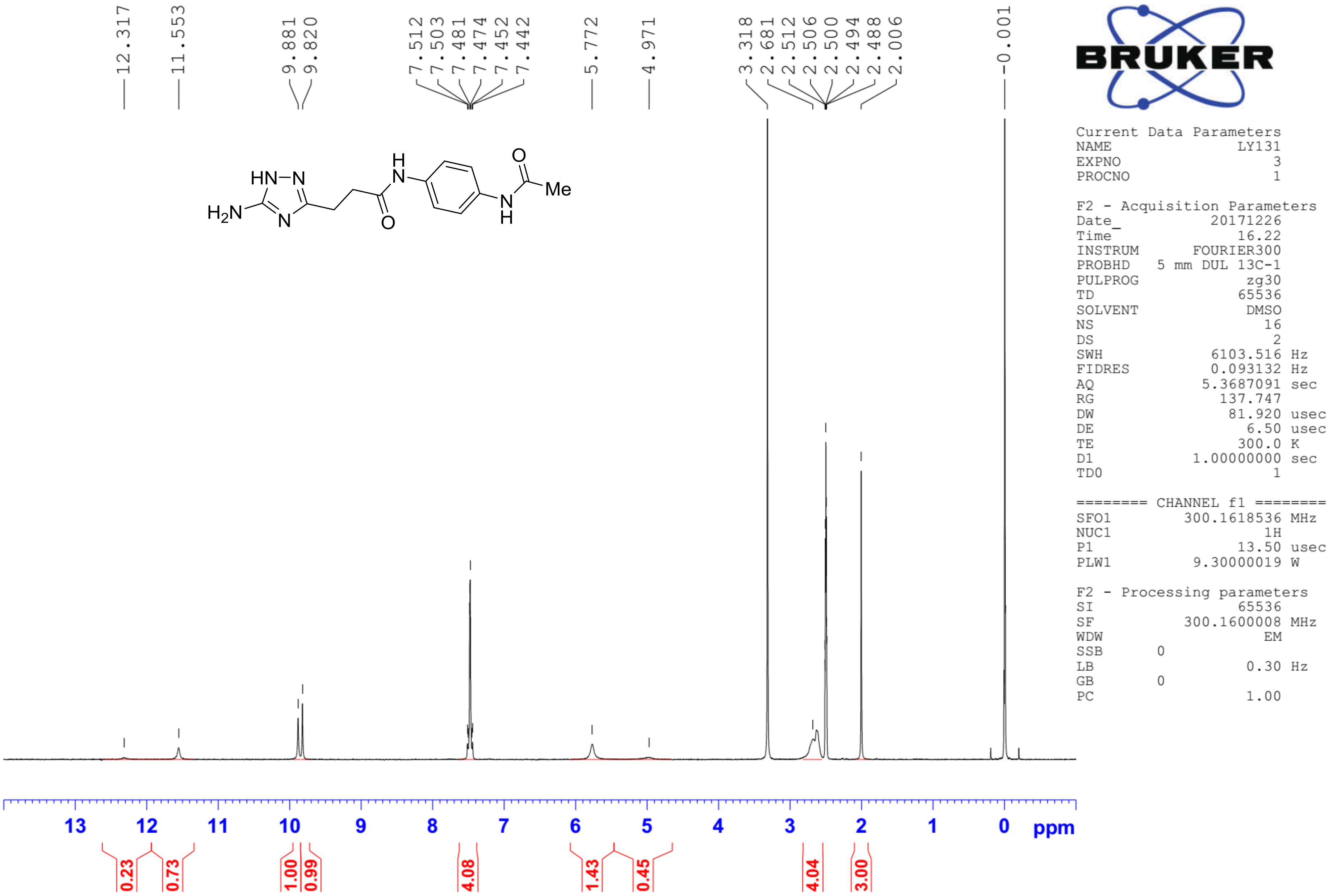

3-(5-Amino-1H-1,2,4-triazol-3-yl)-N-(4-acetamidophenyl)propanamide (5t)

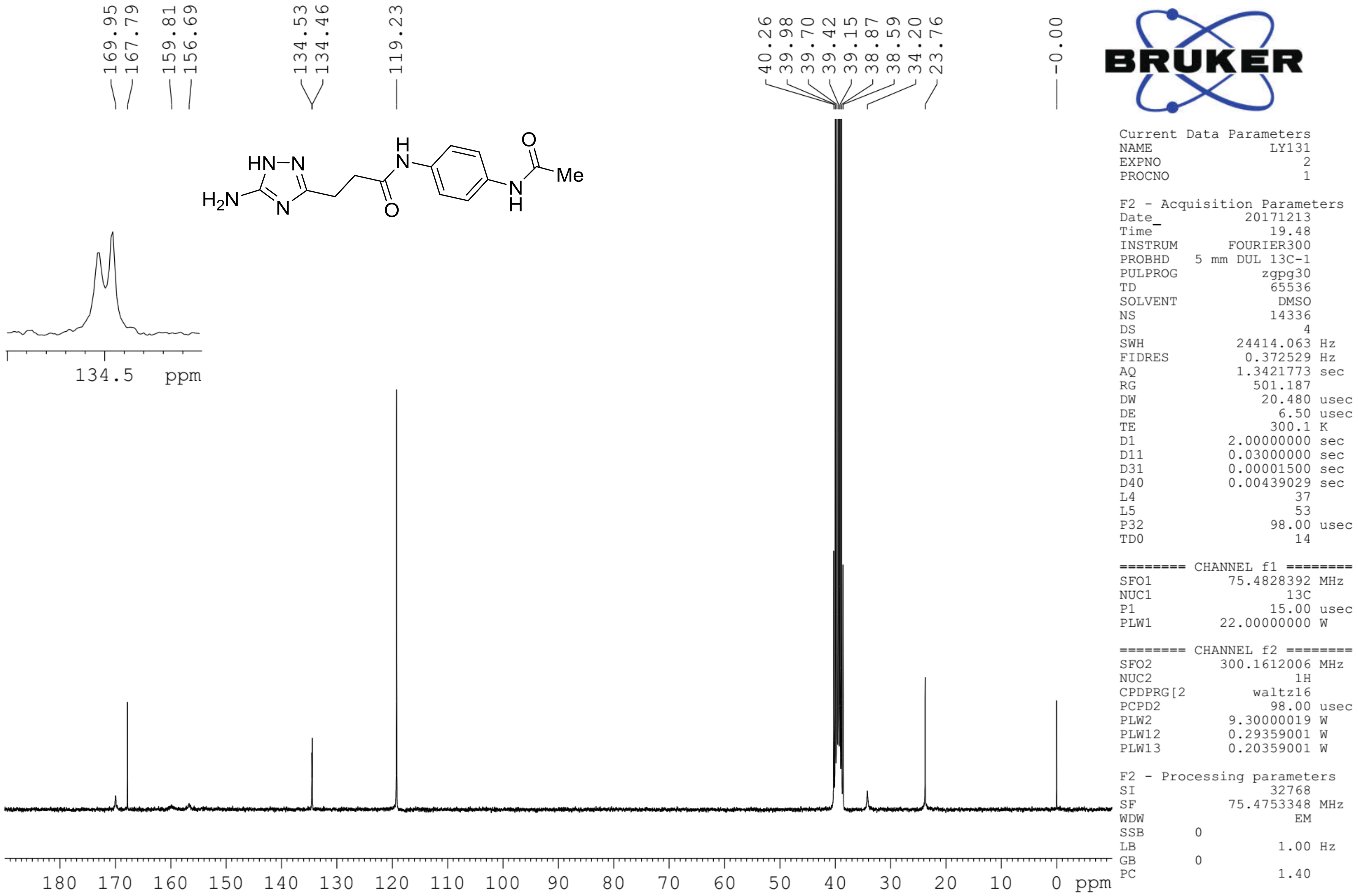

X-ray crystallography: packing and interactions in the crystals of 5j

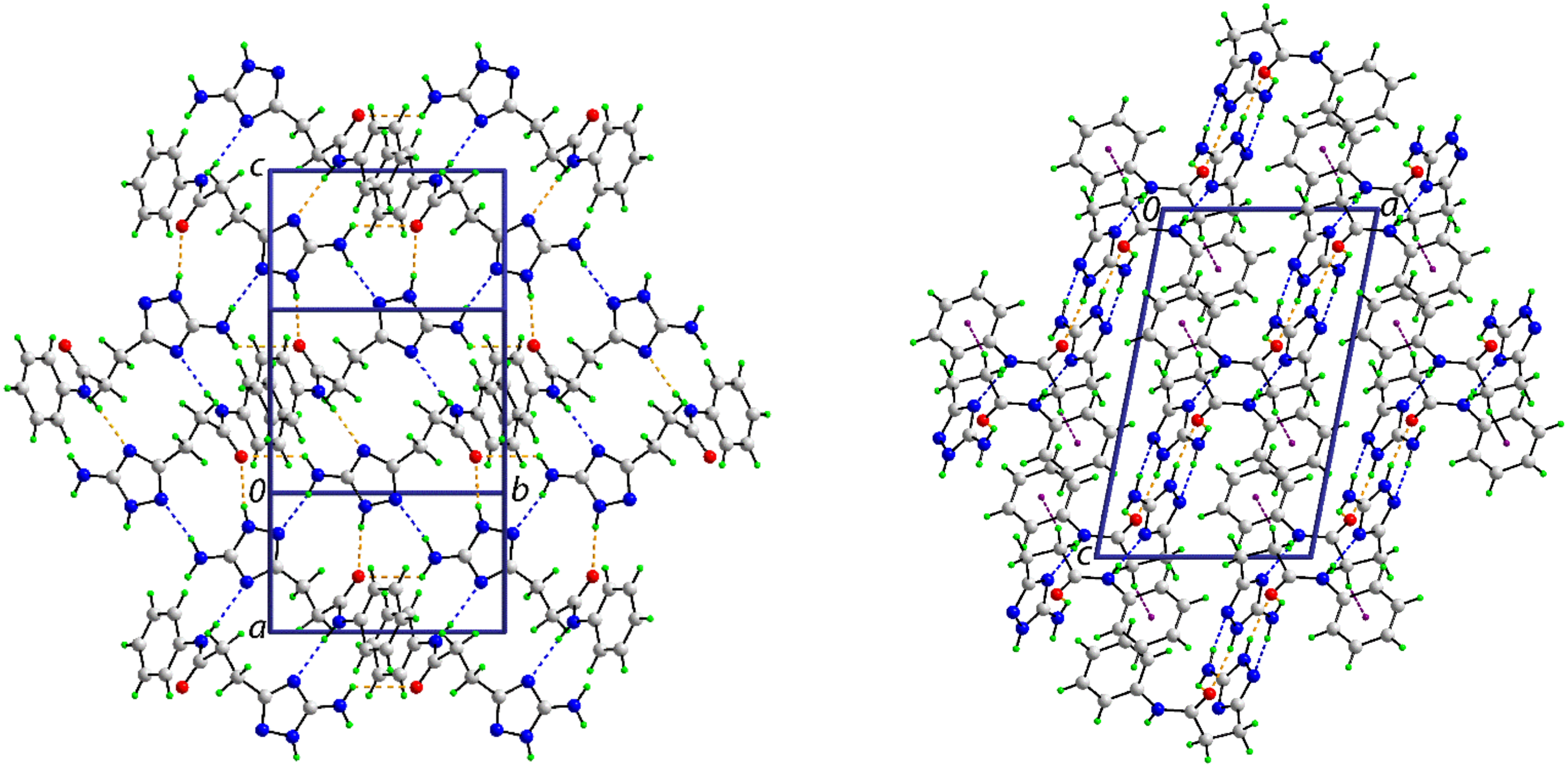

(a) (b)

Figure S1. Molecular packing in **5j**: (a) a view of the supramolecular layer parallel to (1 0 1) sustained by N–H···O and N–H···N hydrogen bonding shown as orange and blue dashed lines, respectively, and (b) a view in projection down the *b*-axis of the unit cell contents. The C–H···π interactions are shown as purple dashed lines.

Table S1. Geometric parameters (Å, °) characterising the identified intermolecular interactions in the crystal of **5j**.

A	H	B	A–H	H···B	A···B	A–H···B	symm. operation
N1	H1n	O8	0.874(10)	1.994(11)	2.8568(13)	169.0(14)	½-x, ½+y, 1½-z
N5	H2n	N2	0.906(13)	1.998(14)	2.8866(15)	166.5(14)	½-x, ½+y, 1½-z
N5	H3n	O8	0.891(15)	2.535(15)	3.1243(14)	124.2(12)	x, 1+y, z
N8	H4n	N4	0.875(13)	2.038(13)	2.9106(14)	174.5(12)	1-x, 1-y, 1-z
C7	H7a	Cg(1)*	0.99	2.77	3.6701(12)	151	1-x, -y, 1-z

Cg(1) is the ring centroid of the C9-C14 ring.
